# Crystal structure of (*S*)-1-*O*-*tert*-butyl­diphenyl­silylglycerol: eight chiral mol­ecules in a triclinic cell

**DOI:** 10.1107/S2056989018012021

**Published:** 2018-08-31

**Authors:** Bogdan Doboszewski, Alexander Y. Nazarenko, Victor N. Nemykin, Maria Joselice e Silva

**Affiliations:** a Departamento de Química, Universidade Federal Rural de Pernambuco, 52171-900 Recife, PE, Brazil; b Chemistry Department, State University of New York, College at Buffalo, 1300 Elmwood Ave, Buffalo, NY 14222-1095, USA; c Department of Chemistry, University of Manitoba, Winnipeg, MB R3T 2N2, Canada; d Departamento de Farmácia, Universidade Federal do Rio Grande do Norte, 59010-180, Natal, RN, Brazil

**Keywords:** crystal structure, (*S*)-1-*O*-*t*-butyl­diphenyl­silylglycerol, chiral, high *Z*′ structure, disorder

## Abstract

The asymmetric unit of the title compound {systematic name: 3-[(*tert*-butyl­diphenyl­sil­yl)­oxy]propane-1,2-diol, C_19_H_26_O_3_Si}, contains eight chiral mol­ecules (*Z*′ = 8). These mol­ecules are connected *via* a complex system of hydrogen bonds into an infinite assembly along the [100] axis; hydro­phobic *tert*-butyl and phenyl groups form an external coating of the assembly. These assemblies are packed by weak inter­molecular inter­actions in a peculiar formation resembling a ‘header bond’ masonry brick wall. Disorder of flexible fragments increases with temperature but the same crystal structure exists from 120 to 220 K (and most probably to the melting point at 334 K).

## Abstract 2

In the triclinic crystal form, the mol­ecules are linked by hydrogen bonds into an infinite assembly propagating along the *a* axis; hydro­phobic *tert*-butyl and phenyl groups form an external coating of the assembly.

## Chemical context   

Glycerol nucleic acids (or glycol nucleic acids, GNA) and flexible nucleic acids (FNA) are two groups of unnatural polymers that have received attention as possible precursors of the present DNA/RNA-based life (Zhang *et al.*, 2010 ▸). A common characteristic feature of both GNA and FNA is the presence of an acyclic three-carbon unit containing a stereogenic center, which gives rise to both possible configurations (*R*) and (*S*). Some nucleoside derivatives containing an acyclic appendix (instead of a ribose or 2-de­oxy­ribose moiety) are very active anti­viral agents: Acyclo­vir, Adefovir, Ganciclovir, Penciclovir, Tenofovir or Cidofovir. For the latter two compounds, this appendix has three carbon atoms and can be built up from chiral glycerol. Guaifenesin [3-(2-meth­oxy­phen­oxy)propane-1,2-diol], a common expectorant medication, is another example of a substituted chiral glycerol. A simple and stereospecific method to obtain 1-*O*-substituted glycerols with a predetermined configuration based on 5-*O*-substituted d- and l-arabinose was realized (Doboszewski & Herdewijn, 2011 ▸) and further expanded by application of 6-*O*-substituted d-gluco­pyran­ose and d-galacto­pyran­ose as presented in this paper.
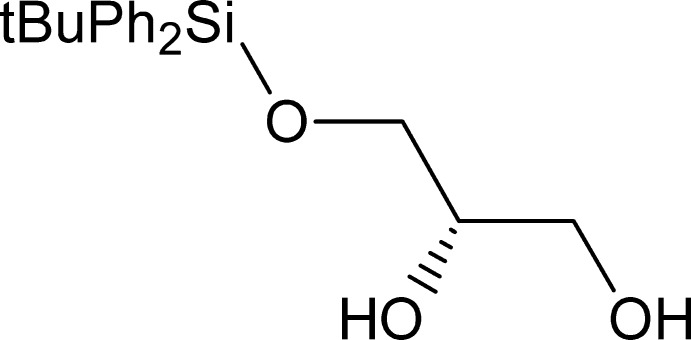


A scattered 1-*O*-substituted glycerol, (*S*)-1-*O*-*tert*-butyl­diphenyl­silylglycerol, the title compound of this paper, together with its enanti­omer, has been used for the research in the field of glycerol nucleic acids (Doboszewski *et al.*, 2013 ▸), in the field of iso-glycerol nucleic acids (Kim *et al.*, 2014 ▸), and to obtain the derivatives displaying β-adrenergic activity (Leftheris & Goodman, 1990 ▸). Here we report the X-ray structure of (*S*)-1-*O*-*tert*-butyl­diphenyl­silylglycerol obtained from 5-*O-tert*-butyl­diphenyl­silyl-d-gluco­pyran­ose (Tsutsui *et al.* 2014 ▸) or 5-*O-*tert-butyl­diphenyl­silyl-d-galacto­pyran­ose (Doboszewski & Herdewijn, 2012 ▸).

## Structural commentary   

The initial crystal structure determination had been performed at 220 K and revealed a complex chiral triclinic structure with *Z* = 8 and multiple disordered fragments. Similar structures often have an inappropriate space-group designation (Marsh, 1999 ▸, 2005 ▸). There is also the possibility of multiple polymorphs existing at different temperatures (Desiraju, 2007 ▸). To reduce the possibility of an erroneous determination of unit-cell parameters or overlooked higher symmetry and to look at this structure at lower temperatures, the experiment was repeated at 123 K and at 173 K using different diffractometer types. Preliminary, low-quality data were also obtained at 220 K (full data not included here). The results were consistent for all three measurements.

Data obtained for 123 K (data set 1) show the lowest degree of disorder and a lower uncertainty of all parameters of the crystal structure; further discussion thus deals mainly with this dataset.

**Figure 1:**
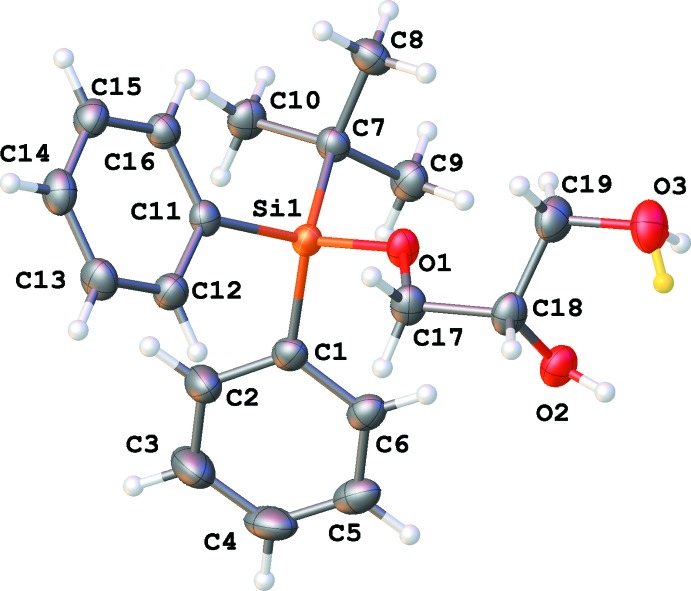
Numbering scheme for mol­ecule 1 of the title compound (50% probability displacement ellipsoids). The disordered H atom (occupancy 0.22 at 123 K) is shown in yellow.

**Figure 6:**
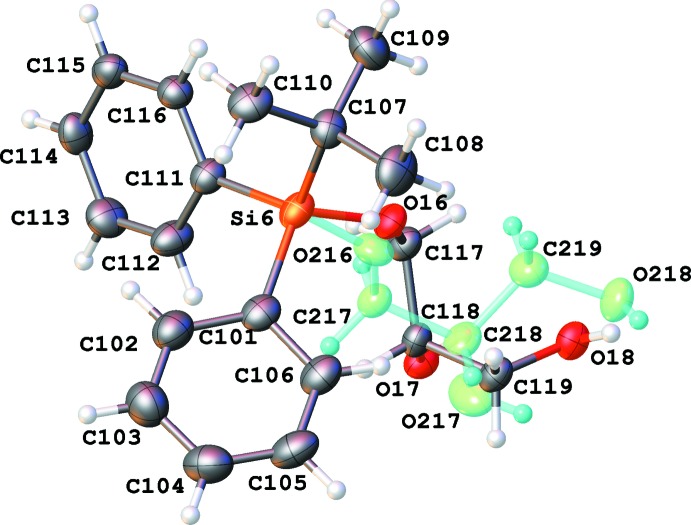
Numbering scheme for mol­ecule 6 of the title compound (50% probability displacement ellipsoids). The second glycerol chain (occupancy 0.22 at 123 K) is shown in green.

**Figure 7:**
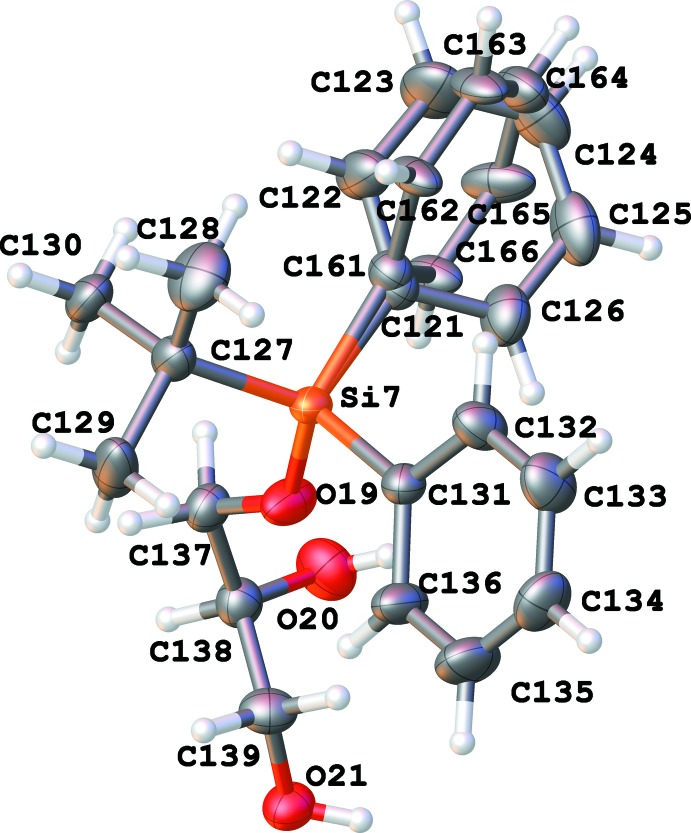
Numbering scheme for mol­ecule 7 of the title compound (50% probability displacement ellipsoids).

There are eight independent mol­ecules of the title compound in a unit cell in space group *P*1 (Figs. 1 ▸–8 ▸ ▸ ▸ ▸ ▸ ▸ ▸). Both the bond distances and angles of all moieties are unexceptional and consistent with standard values. As a result of the relatively long Si—C bond, there is little hindrance for rotation of the phenyl and *tert*-butyl groups in the *tert*-butyl­diphenyl­silyl fragments. This results in higher vibrational ellipsoids for the methyl groups and for some of the phenyl groups; in mol­ecule 7, there are two visibly disordered phenyl rings (Fig. 7 ▸). Flexible glycerol fragments can occupy different positions. In two cases, this disorder was substantial and the fragments were refined as disordered (Figs. 6 ▸ and 8 ▸). Disorder of all groups visibly increases with temperature: for example, the occupancy of the minor component of the glycerol moiety in mol­ecule 8 is 0.12 at 123 K, 0.29 at 173 K and 0.37 at 220 K; for mol­ecule 6 it is 0.22 at 123 K, 0.36 at 173 K and 0.46 at 220 K. Several *tert*-butyl and phenyl groups are also becoming disordered at 220 K.

**Figure 9:**
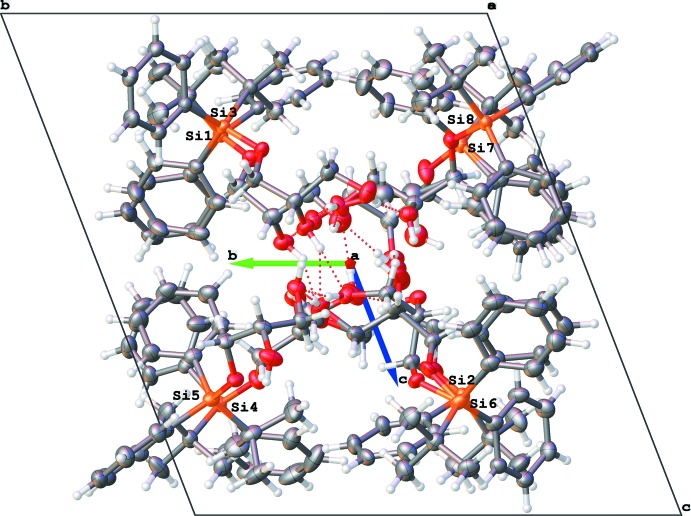
View of the unit-cell contents: the hydro­philic fragments are pointing inwards and the hydro­phobic substituents form an outer ‘coat’.

**Figure 10:**
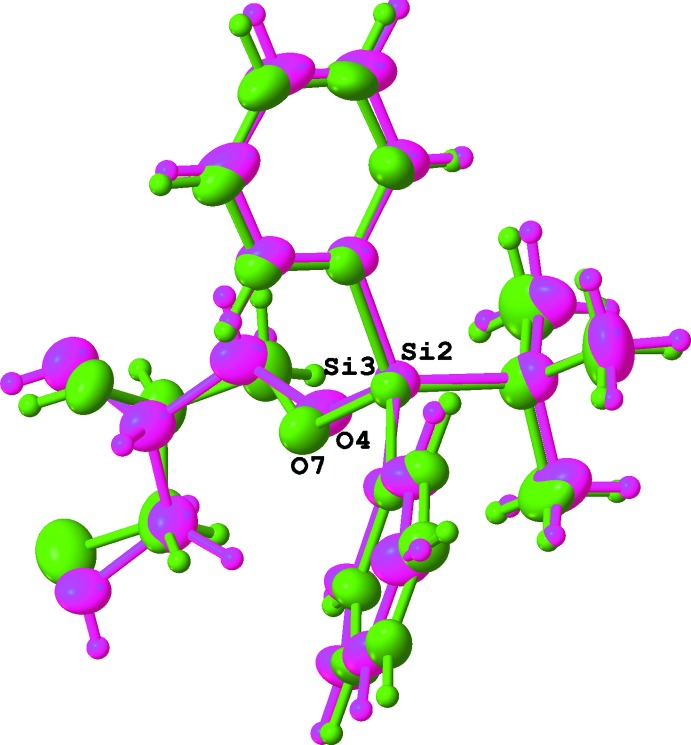
Overlay of two mol­ecules 2 and 3 after inversion of fragment 3.

**Figure 11:**
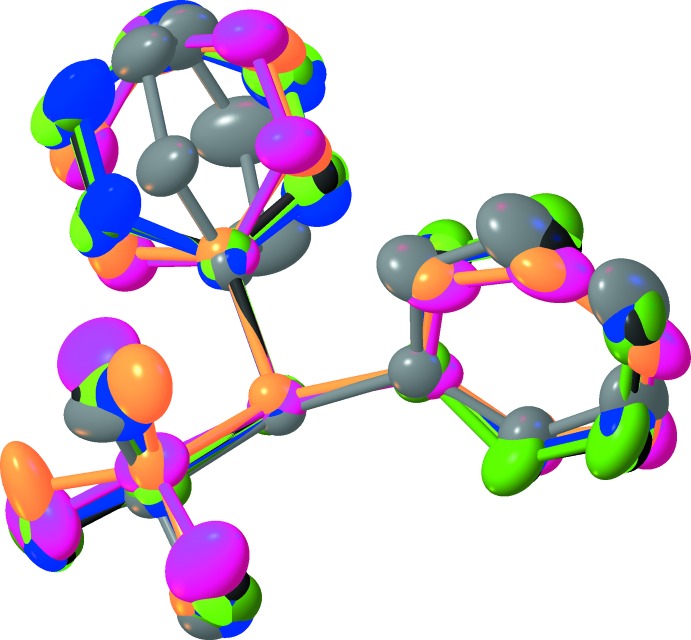
Overlay of the *tert*-butyl­diphenyl­silanyl fragments. Colour key: 1 – black, 2 – light green, 3 – red, 4 – grey, 5 – purple, 6 – green, 7 – blue, 8 – orange.

The eight mol­ecules form an assembly with the hydro­phobic phenyl and butyl groups making an external ‘coat’ and the hydro­philic chiral glycerol moieties forming the inner core of the unit cell (Fig. 9 ▸). There is visible pseudosymmetry [see Zorky (1996 ▸) for the definition and discussion of this phenomenon] in this assembly. The triclinic system allows only an inversion centre as a symmetry element; however, it is prohibited by chirality in this case. Nevertheless, when the chiral glycerol groups are excluded from consideration, the whole structure becomes close to centrosymmetric: an overlay of two paired mol­ecules is shown in Fig. 10 ▸. An attempt to overlay all *tert-*butyl­diphenyl­silyl moieties (Fig. 11 ▸) reveals a relatively good fit for the *tert*-butyl groups and one of the phenyl groups; inter­estingly, almost free rotation is observed for the second phenyl group.

## Supra­molecular features   

**Figure 12:**
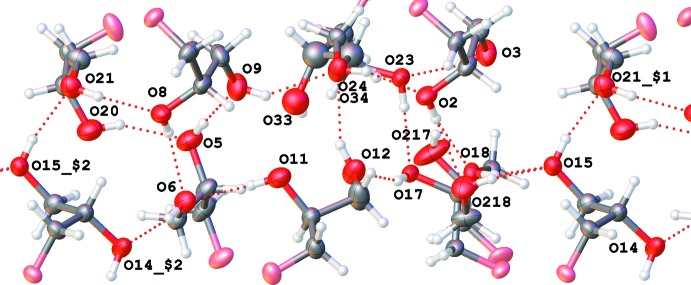
Sequence of O—H⋯O hydrogen bonds (dashed lines) connecting glycerol moieties. View along the [010] axis. Symmetry codes: (1) *x* + 1, *y*, *z*; (2) *x* − 1, *y*, *z.* Silanol oxygen atoms are pink.

**Figure 13:**
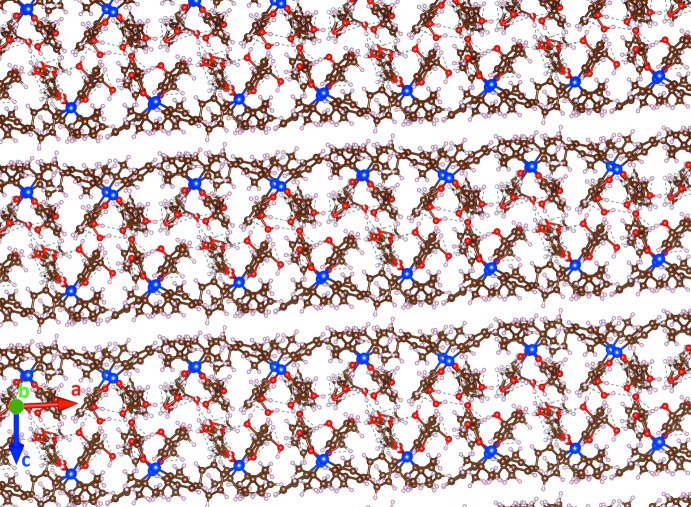
Packing diagram: view along the [010] axis.

**Figure 14:**
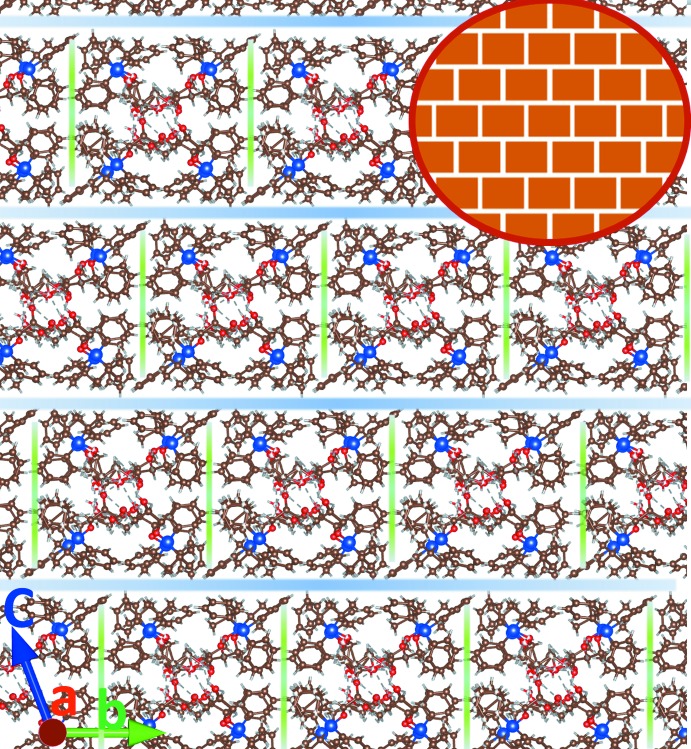
Packing diagram: view along the [100] axis. The ‘header bond’ brick wall motif is highlighted.

Each of the eight mol­ecules has two hydroxyl groups; each of them can be both a donor and an acceptor of a strong O—H⋯O hydrogen bond (Gilli & Gilli, 2013 ▸). Indeed, fifteen such bonds are observed (Tables 1 ▸ and 2 ▸, Fig. 12 ▸). One weaker hydrogen bond (O14—H14*G*⋯O13) connects a hydroxyl group to a neighbouring ether oxygen atom. The whole system is additionally stabilized by weaker C—H⋯O inter­actions (two of them are shown in Tables 1 and 2 ▸ ▸). The hydroxyl groups of the disordered fragments mostly follow the same direction of hydrogen bonding. The independent mol­ecules are bound by this complex system of hydrogen bonds, forming an infinite assembly (a ‘beam’) along the [100] axis (analogous to a reinforced concrete beam in construction, with hydrogen bonding serving as the reinforcement, see Fig. 13 ▸). These beams have an almost rectangular cross-section (Figs. 9 ▸ and 14 ▸). They are packed in layers parallel to the (001) plane by weak inter­molecular inter­actions with no hydrogen bonds or stacking. These layers are assembled in a peculiar fashion, resembling a ‘header bond’ brick wall in masonry (Fig. 14 ▸). An ideal ‘brick wall’ tiling belongs to the rectangular plane symmetry group **c2mm** (No. 9; see Hahn, 2006 ▸). In our case, it is distorted to an oblique **p1.** There are no strong contacts between the layers; more careful examination even shows some small voids between them. However, again following the masonry analogy, such packing should be relatively stable simply for mechanical reasons (similar to a ‘dry wall’ with no mortar). The resulting three-dimensional crystal is stable despite multiple disorder inside the crystal cell.

The self-assembly of the relatively simple title mol­ecule into a complex infinite entity can serve as an illustration of the feasibility of glycerol-based assemblies in biochemical systems.

## Database survey   

There are 55 structures of substituted glycerol compounds deposited in the Cambridge Structural Database (CSD Version 5.39; Groom *et al.*, 2016 ▸). Of these structures, two are glycerolphosphates; all others are organic compounds with a carbon atom connected to the terminal oxygen of the glycerol. Therefore, the current structure is the first silyl derivative of glycerol and the first non-carbon substituent neutral organic compound of that type. Two of the substituted glycerol structures [refcodes OKOXIW (Bredikhin *et al.* (2010 ▸) and WASHIJ (Bredikhin *et al.* (2008 ▸)] report the space group *P*1 and high Z′ (8 and 4, respectively). However, analysis of the corresponding CIF files using the ADDSYM procedures of *PLATON* (Spek, 2009 ▸) suggests much higher symmetry and a smaller Z′: for OKOXIW the space group is *I*2 and *Z*′ = 4; for WASHIJ it is *Iba*2 and *Z*′ = 1. These examples demonstrate importance of additional caution while working with high *Z*′ numbers (Marsh, 1999 ▸). Most of these substituted glycerols are chiral compounds. Spontaneous resolution of such compounds (guaifenesin and several similar mol­ecules) was investigated by Bredikhin *et al.* (2009 ▸). Curiously, glycerol itself spontaneously crystallizes (Kusukawa *et al.*, 2013 ▸) in a chiral space group, *P*2_1_2_1_2_1_.

The exact number of all known structures that crystallize in space group *P*1 with *Z* = 8 is ambiguous. Structures with large *Z*′ have been reviewed in detail by Steed & Steed (2015 ▸) and Brock (2016 ▸); databases of high-*Z*′ structures were created based on CSD data. A direct search of the CSD (CSD Version 5.39; Groom *et al.*, 2016 ▸) yields 41 entries for *P*1, *Z* = 8. However, some of them are obvious typographical errors and several are unambiguously convertible to higher symmetry and consequently lower *Z*′. Most of the remaining (around 30) unambiguous structures are pseudocentrosymmetric, with an 80–95% fit for an exact centrosymmetric structure. Nevertheless, for a triclinic structure, the chirality of the mol­ecules serves as a solid proof of space group *P*1, similar to our structure.

There are numerous structures of silyl-substituted mol­ecules similar to the title compound (*e.g*. there are 3874 structures with a di­phenyl­silicon moiety and 475 *tert*-butyl­diphenyl­silyl compounds). Of these, eight are compounds with high *Z*′, five of which are chiral. The triclinic centrosymmetric structure of *tert*-butyl­diphenyl­silanol (Habtemariam *et al.*, 2015 ▸) shows *Z* = 8 (*Z*′ = 4); four independent mol­ecules form a pseudo­tetra­gonal motif around four hydrogen bonds connecting the silanol groups in a fashion that remotely resembles the current structure. It was suggested by Prince *et al.* (2002 ▸) that weak inter­actions induce asymmetry in the crystal structures of triaryl derivatives of group 14 elements (Si, Ge, Sn), resulting in an abnormally large number of structures with high *Z*′.

## Synthesis and crystallization   

The title compound was prepared from 5-*O-tert*-butyl­diphen­yl­silyl-d-gluco­pyran­ose (Tsutsui *et al.* 2014 ▸) or 5-*O-tert*-butyl­diphenyl­silyl-d-galacto­pyran­ose (Doboszewski & Herde­wijn, 2012 ▸) using the procedure published before for 5-*O-tert*-butyl­diphenyl­silyl-d- or -l-arabino­furan­ose (Doboszewski & Herdewijn, 2011 ▸). To a solution of 5-*O-tert*-butyl­diphenyl­silyl-d-gluco­pyran­ose or 5-*O-tert*-butyl­diphenyl­silyl-d-galacto­pyran­ose (2.0 g, 5.1 mmol) in 96% ethanol, 20 mL, was added portionwise a solution of NaIO_4_ (3.8 g, 17.8 mmol) in water (15 mL) over a period of 10 min in a magnetically stirred ice bath. The solution became turbid within a few seconds. After the end of addition, the mixture was left at room temperature for 1.5 h. The white solid was removed by filtration (sintered glass) and the filtrate was cooled in an ice bath. NaBH_4_ (0.15 g, 4 mmol) was added with manual swirling. After 1h, the reaction mixture was transferred to a separatory funnel and extraction was performed (H_2_O–CH_2_Cl_2_). The organic phase was washed with water, dried (MgSO_4_) and the solids were removed by filtration. Vacuum evaporation at 303 K furnished a glassy material which was purified by chromatography on silica gel with hexa­ne–ethyl acetate (6:5) to yield the title compound (1.03 g, 61%). *R*_f_ 0.48, m.p. 332–334 K (hexa­ne–diethyl ether), α_D_ −5.2 (*c* 6, dioxane). ^1^H NMR (CDCl_3_) and ^13^C NMR (CDCl_3_) are identical to published data (Doboszewski & Herdewijn, 2011 ▸). Exact mass (electrospray): calculated for [C_19_H_26_O_3_Si + Na]^+^ = 353.1549, found: 353.1540.

## Refinement   

Crystal data, data collection and structure refinement details are summarized in Table 3 ▸. All C-bound hydrogen atoms were placed in calculated positions and treated as riding with *U*_iso_(H) = 1.2*U*_eq_(C) or 1.5*U*_eq_(C_meth­yl_). The O-bound H atoms were refined as riding with O—H = 0.84 Å with *U*_iso_(H) = 1.5*U*_eq_(O).

The difference-Fourier map indicated possible disorder for the H atom of hydroxyl group O3 (Fig. 1 ▸). The occupancies of this disordered group were set to be equal to those of the neighboring disordered glycerol fragment of mol­ecule 6. It was not possible to locate and refine a hydrogen atom of hydroxyl group O34 with occupancy of 0.13 at 123 K; its position was set as identical to that of H24 (Fig. 6 ▸). At 173 K, this atom was placed at a calculated position, which appeared to be very close to the previous assumption.

In a disordered phenyl group (atoms C121–C126 and C161-C166), the bond distances were restrained to make the geometry of both rings similar; an additional set of restraints was applied to make the phenyl rings symmetrical (Fig. 7 ▸). The anisotropic parameters of atoms C121 and C161 were set to be equal. Two disordered glycerol fragments in mol­ecules 6 and 8 were resolved; restraints were applied to all inter­atomic distances in these fragments to make equivalent distances approximately equal. The anisotropic parameters of two closely located pairs of atoms (C117 and C217, and O24 and O34) were set as equal (Figs. 6 ▸ and 8 ▸). An additional set of restraints was applied to make the Si6— O16 and Si—O216 as well as the Si8—O22 and S8—O32 bond distances approximately equal. An anti-bumping restraint was added to prevent a short distance between calculated hydrogen-atom positions involving the low-occupancy fragment in mol­ecule 6.

The chirality of the title compound was known from the synthetic route. Analysis of the absolute structure using anomalous scattering (Flack, 1983 ▸; Spek, 2009 ▸) was undertaken for three different crystals and confirmed the original assignment (Table 3 ▸).

## Supplementary Material

Crystal structure: contains datablock(s) 1, 2. DOI: 10.1107/S2056989018012021/zl2734sup1.cif

Structure factors: contains datablock(s) 1. DOI: 10.1107/S2056989018012021/zl27341sup2.hkl

Supporting information file. DOI: 10.1107/S2056989018012021/zl27341sup4.cdx

Structure factors: contains datablock(s) 2. DOI: 10.1107/S2056989018012021/zl27342sup3.hkl

Supporting information file. DOI: 10.1107/S2056989018012021/zl27341sup5.cml

CCDC references: 1863803, 1863802

Additional supporting information:  crystallographic information; 3D view; checkCIF report

## Figures and Tables

**Figure 2:**
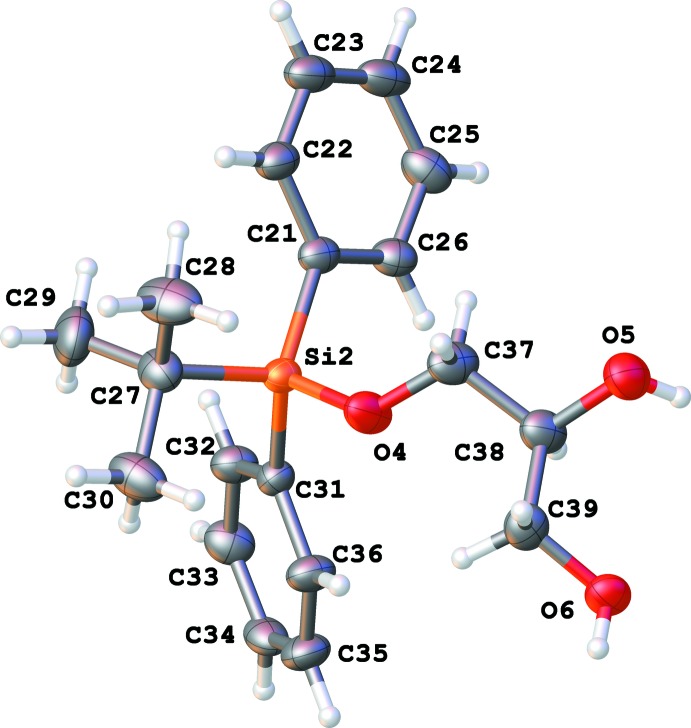
Numbering scheme for mol­ecule 2 of the title compound (50% probability displacement ellipsoids).

**Figure 3:**
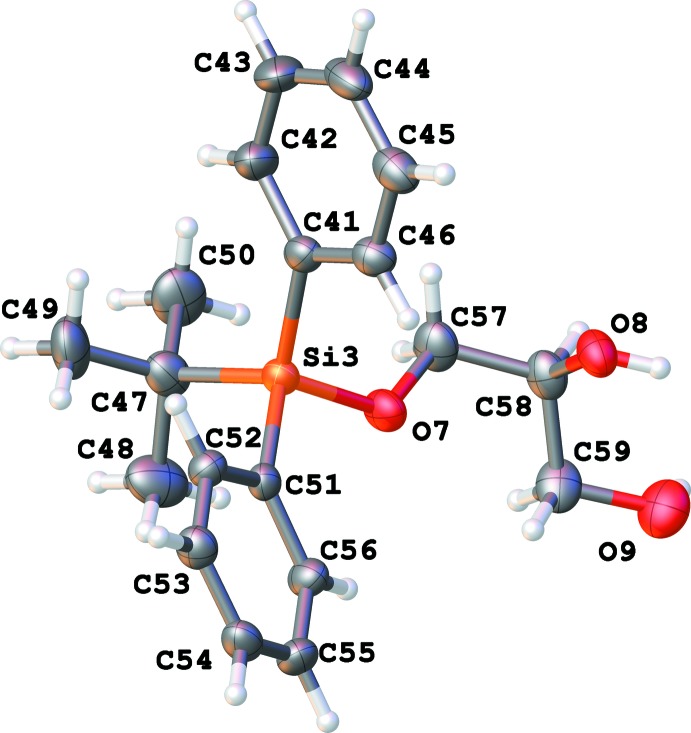
Numbering scheme for mol­ecule 3 of the title compound (50% probability displacement ellipsoids).

**Figure 4:**
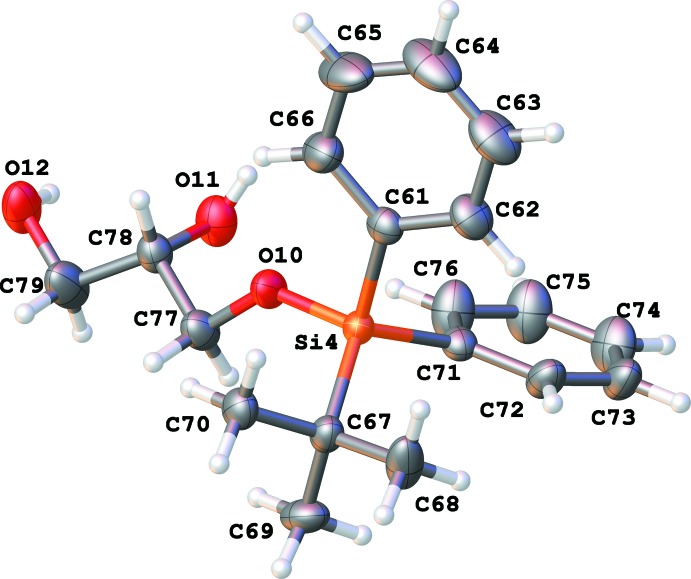
Numbering scheme for mol­ecule 4 of the title compound (50% probability displacement ellipsoids).

**Figure 5:**
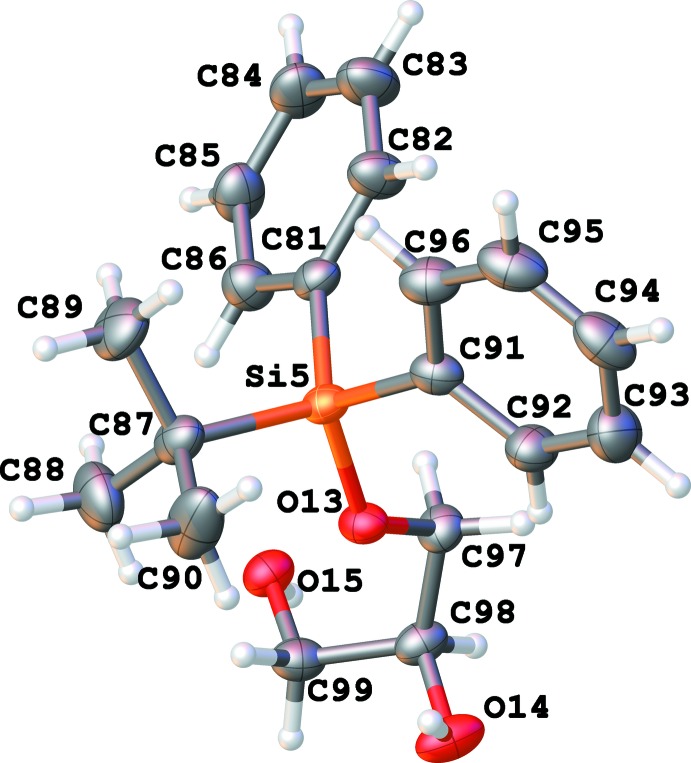
Numbering scheme for mol­ecule 5 of the title compound (50% probability displacement ellipsoids).

**Figure 8:**
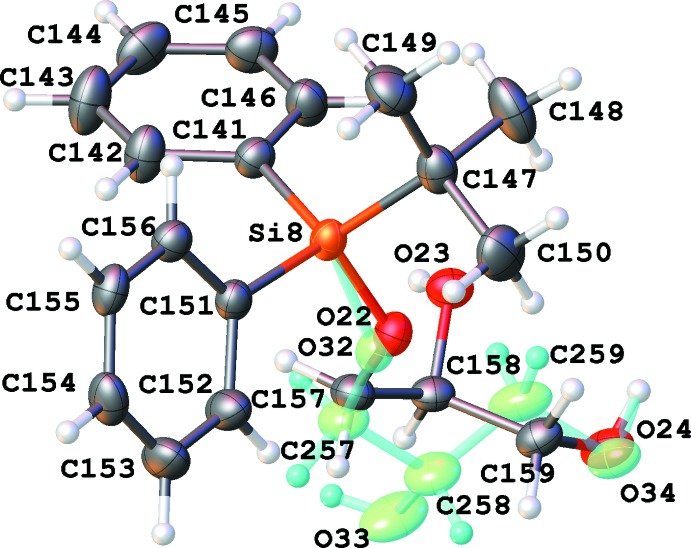
Numbering scheme for mol­ecule 8 of the title compound (50% probability displacement ellipsoids). The second glycerol chain (occupancy 0.13 at 123 K) is shown in green.

**Table 1:** Hydrogen-bond geometry (Å, °) at 123 K[Chem scheme1]

*D*—H⋯*A*	*D*—H	H⋯*A*	*D*⋯*A*	*D*—H⋯*A*
O2—H2⋯O18	0.84	2.02	2.840 (3)	166
O2—H2⋯O218	0.84	2.06	2.796 (9)	146
O3—H3*A*⋯O23	0.84	1.93	2.757 (3)	167
O5—H5*A*⋯O9	0.84	1.82	2.659 (3)	174
O6—H6*A*⋯O14^i^	0.84	1.87	2.701 (3)	168
O8—H8⋯O6	0.84	2.06	2.812 (3)	149
O9—H9⋯O24	0.84	2.14	2.835 (5)	140
O11—H11⋯O6	0.84	1.95	2.787 (3)	177
O12—H12*G*⋯O24	0.84	2.08	2.867 (4)	155
O14—H14*G*⋯O13	0.84	2.42	2.838 (2)	111
O15—H15*H*⋯O21^ii^	0.84	1.85	2.679 (3)	169
O17—H17⋯O12	0.84	1.86	2.639 (3)	154
O18—H18⋯O15	0.84	1.90	2.722 (3)	164
O218—H218⋯O15	0.84	1.80	2.630 (10)	171
O20—H20⋯O5	0.84	1.99	2.785 (3)	157
O21—H21⋯O8	0.84	1.89	2.731 (3)	175
O23—H23⋯O17	0.84	1.86	2.689 (3)	171
O24—H24⋯O2	0.84	1.92	2.727 (5)	161
C119—H11*G*⋯O3	0.99	2.45	3.283 (4)	141
C138—H138⋯O3^i^	1.00	2.50	3.314 (3)	138

Symmetry codes: (i) 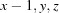; (ii) 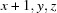.

**Table 2:** Hydrogen-bond geometry (Å, °) at 173 K[Chem scheme1]

*D*—H⋯*A*	*D*—H	H⋯*A*	*D*⋯*A*	*D*—H⋯*A*
O2—H2⋯O18	0.84	2.03	2.841 (4)	163
O2—H2⋯O218	0.84	2.11	2.828 (7)	143
O3—H3*A*⋯O23	0.84	1.93	2.752 (4)	165
O5—H5*A*⋯O9	0.84	1.81	2.651 (4)	176
O6—H6*A*⋯O14^i^	0.84	1.90	2.707 (3)	161
O8—H8⋯O6	0.84	2.06	2.814 (3)	149
O9—H9⋯O24	0.84	2.06	2.84 (2)	154
O11—H11⋯O6	0.84	1.95	2.782 (4)	174
O12—H12*G*⋯O24	0.84	2.33	3.00 (2)	137
O14—H14*G*⋯O13	0.84	2.44	2.839 (3)	110
O15—H15*H*⋯O21^ii^	0.84	1.85	2.676 (3)	169
O17—H17⋯O12	0.84	1.83	2.615 (4)	156
O18—H18⋯O15	0.84	1.93	2.745 (4)	164
C119—H11*G*⋯O3	0.99	2.39	3.222 (7)	141
O218—H218⋯O15	0.84	1.76	2.599 (8)	176
O20—H20⋯O5	0.84	2.00	2.794 (4)	157
O21—H21⋯O8	0.84	1.89	2.727 (4)	174
C138—H138⋯O3^i^	1.00	2.50	3.320 (5)	139
O23—H23⋯O17	0.84	1.87	2.697 (5)	169
O24—H24⋯O2	0.84	1.96	2.78 (2)	163

Symmetry codes: (i) 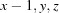; (ii) 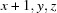.

**Table 3:** Experimental details

	123 K	173 K
Crystal data		
Chemical formula	C_19_H_26_O_3_Si	C_19_H_26_O_3_Si
*M*_r_	330.49	330.49
Crystal system, space group	Triclinic, *P*1	Triclinic, *P*1
*a*, *b*, *c* (Å)	14.7668 (2), 15.5936 (2), 17.2270 (12)	14.7922 (10), 15.6306 (10), 17.2048 (11)
α, β, γ (°)	111.053 (8), 91.616 (7), 92.898 (7)	110.901 (2), 91.705 (2), 92.851 (2)
*V* (Å^3^)	3692.7 (3)	3706.8 (4)
*Z*	8	8
Radiation type	Mo *K*α	Mo *K*α
μ (mm^−1^)	0.14	0.14
Crystal size (mm)	0.32 × 0.2 × 0.2	0.6 × 0.45 × 0.37

Data collection		
Diffractometer	Rigaku R-AXIS RAPID II imaging plate	Bruker PHOTON-100 CMOS
Absorption correction	Multi-scan (*ABSCOR*; Higashi, 1999 ▸)	Multi-scan (*SADABS*; Krause *et al.*, 2015 ▸)
*T*_min_, *T*_max_	0.92, 0.97	0.907, 0.950
No. of measured, independent and observed [*I* > 2σ(*I*)] reflections	124586, 33607, 31303	120838, 34177, 28468
*R*_int_	0.026	0.034
(sin θ/λ)_max_ (Å^−1^)	0.649	0.652

Refinement		
*R*[*F* ^2^ > 2σ(*F* ^2^)], *wR*(*F* ^2^), *S*	0.037, 0.097, 1.06	0.046, 0.118, 1.03
No. of reflections	33607	34177
No. of parameters	1839	1857
No. of restraints	378	272
H-atom treatment	H atoms treated by a mixture of independent and constrained refinement	H-atom parameters constrained
Δρ_max_, Δρ_min_ (e Å^−3^)	0.43, −0.23	0.33, −0.25
Absolute structure	Flack *x* determined using 14181 quotients [(*I* ^+^)−(*I* ^−^)]/[(*I* ^+^)+(*I* ^−^)] (Parsons et al., 2013 ▸)	Flack *x* determined using 11980 quotients [(*I* ^+^)−(*I* ^−^)]/[(*I* ^+^)+(*I* ^−^)] (Parsons et al., 2013 ▸
Absolute structure parameter	−0.011 (14)	−0.008 (18)

Computer programs: *CrystalClear* (Rigaku, 2009 ▸), *APEX2* and *SAINT* (Bruker, 2013 ▸), *HKL-2000* (Otwinowski & Minor, 1997 ▸), *SHELXT* (Sheldrick, 2015*a* ▸), *SHELXL* (Sheldrick, 2015*b* ▸), *OLEX2* (Dolomanov *et al.*, 2009 ▸) and *VESTA3* (Momma & Izumi, 2008 ▸).

## supplementary crystallographic information

### 3-[(tert-Butyldiphenylsilyl)oxy]propane-1,2-diol (1) Crystal data

**Table:**

C_19_H_26_O_3_Si	*F*(000) = 1424
*M**_r_* = 330.49	*D*_x_ = 1.189 Mg m^−^^3^
Triclinic, *P*1	Melting point: 334 K
*a* = 14.7668 (2) Å	Mo *K*α radiation, λ = 0.71075 Å
*b* = 15.5936 (2) Å	Cell parameters from 124590 reflections
*c* = 17.2270 (12) Å	θ = 3.0–27.5°
α = 111.053 (8)°	µ = 0.14 mm^−^^1^
β = 91.616 (7)°	*T* = 123 K
γ = 92.898 (7)°	Block, colourless
*V* = 3692.7 (3) Å^3^	0.32 × 0.2 × 0.2 mm
*Z* = 8	

### 3-[(tert-Butyldiphenylsilyl)oxy]propane-1,2-diol (1) Data collection

**Table:**

Rigaku R-AXIS RAPID II imaging plate diffractometer	31303 reflections with *I* > 2σ(*I*)
Radiation source: sealedtube	*R*_int_ = 0.026
ω scans	θ_max_ = 27.5°, θ_min_ = 3.0°
Absorption correction: multi-scan (ABSCOR; Higashi, 1999)	*h* = −19→19
*T*_min_ = 0.92, *T*_max_ = 0.97	*k* = −20→20
124586 measured reflections	*l* = −22→22
33607 independent reflections	

### 3-[(tert-Butyldiphenylsilyl)oxy]propane-1,2-diol (1) Refinement

**Table:**

Refinement on *F*^2^	Hydrogen site location: mixed
Least-squares matrix: full	H atoms treated by a mixture of independent and constrained refinement
*R*[*F*^2^ > 2σ(*F*^2^)] = 0.037	*w* = 1/[σ^2^(*F*_o_^2^) + (0.0626*P*)^2^ + 0.2573*P*] where *P* = (*F*_o_^2^ + 2*F*_c_^2^)/3
*wR*(*F*^2^) = 0.097	(Δ/σ)_max_ = 0.001
*S* = 1.06	Δρ_max_ = 0.43 e Å^−^^3^
33607 reflections	Δρ_min_ = −0.23 e Å^−^^3^
1839 parameters	Absolute structure: Flack *x* determined using 14181 quotients [(*I*^+^)-(*I*^-^)]/[(*I*^+^)+(*I*^-^)] (Parsons et al., 2013)
378 restraints	Absolute structure parameter: −0.011 (14)

### 3-[(tert-Butyldiphenylsilyl)oxy]propane-1,2-diol (1) Special details

**Table:**

Geometry. All esds (except the esd in the dihedral angle between two l.s. planes) are estimated using the full covariance matrix. The cell esds are taken into account individually in the estimation of esds in distances, angles and torsion angles; correlations between esds in cell parameters are only used when they are defined by crystal symmetry. An approximate (isotropic) treatment of cell esds is used for estimating esds involving l.s. planes.

### 3-[(tert-Butyldiphenylsilyl)oxy]propane-1,2-diol (1) Fractional atomic coordinates and isotropic or equivalent isotropic displacement parameters (Å^2^)

**Table:**

	*x*	*y*	*z*	*U*_iso_*/*U*_eq_	Occ. (<1)
Si1	0.61555 (4)	0.63300 (4)	0.22563 (4)	0.02302 (12)	
O1	0.66841 (11)	0.57243 (11)	0.27214 (10)	0.0282 (3)	
O2	0.69021 (13)	0.54372 (13)	0.42668 (11)	0.0354 (4)	
H2	0.708929	0.529686	0.466784	0.053*	
O3	0.79590 (13)	0.38796 (14)	0.35641 (14)	0.0432 (5)	
H3A	0.747580	0.376335	0.376358	0.065*	0.786 (3)
H3B	0.798061	0.416581	0.408144	0.065*	0.214 (3)
C1	0.55248 (16)	0.72079 (16)	0.30582 (15)	0.0283 (5)	
C2	0.5266 (2)	0.80169 (18)	0.29668 (18)	0.0414 (6)	
H2A	0.543812	0.814491	0.249026	0.050*	
C3	0.4763 (3)	0.8640 (2)	0.3557 (2)	0.0553 (9)	
H3	0.458785	0.918285	0.347930	0.066*	
C4	0.4517 (2)	0.8461 (2)	0.4261 (2)	0.0508 (8)	
H4	0.417658	0.888616	0.466707	0.061*	
C5	0.47646 (19)	0.7676 (2)	0.43711 (16)	0.0403 (6)	
H5	0.459522	0.755771	0.485271	0.048*	
C6	0.52646 (17)	0.70526 (18)	0.37768 (15)	0.0320 (5)	
H6	0.543315	0.651041	0.385932	0.038*	
C7	0.53441 (16)	0.54571 (16)	0.14641 (15)	0.0269 (5)	
C8	0.58273 (19)	0.46093 (17)	0.09337 (17)	0.0348 (5)	
H8A	0.538058	0.415045	0.055890	0.052*	
H8B	0.613331	0.434417	0.129883	0.052*	
H8C	0.627658	0.479199	0.060480	0.052*	
C9	0.46171 (18)	0.51413 (19)	0.19453 (17)	0.0350 (5)	
H9A	0.420584	0.465781	0.155153	0.053*	
H9B	0.427066	0.566548	0.225685	0.053*	
H9C	0.491215	0.489981	0.233360	0.053*	
C10	0.48619 (17)	0.58806 (19)	0.08976 (16)	0.0331 (5)	
H10G	0.438159	0.544153	0.055205	0.050*	
H10H	0.530212	0.602237	0.053857	0.050*	
H10I	0.459475	0.644767	0.124132	0.050*	
C11	0.70220 (15)	0.69357 (15)	0.18192 (14)	0.0250 (4)	
C12	0.75406 (17)	0.77016 (17)	0.23650 (15)	0.0311 (5)	
H12	0.741796	0.792289	0.293952	0.037*	
C13	0.82295 (18)	0.81489 (19)	0.20931 (17)	0.0357 (5)	
H13	0.857709	0.866182	0.247907	0.043*	
C14	0.84048 (18)	0.78426 (19)	0.12586 (17)	0.0354 (5)	
H14	0.887069	0.814852	0.106715	0.042*	
C15	0.79037 (18)	0.70919 (18)	0.07017 (16)	0.0327 (5)	
H15	0.802506	0.688187	0.012704	0.039*	
C16	0.72214 (16)	0.66420 (16)	0.09798 (15)	0.0280 (5)	
H16	0.688373	0.612382	0.059032	0.034*	
C17	0.74396 (16)	0.61061 (17)	0.33005 (16)	0.0306 (5)	
H17A	0.729997	0.671327	0.370640	0.037*	
H17B	0.797811	0.619567	0.300173	0.037*	
C18	0.76389 (17)	0.54598 (18)	0.37526 (16)	0.0323 (5)	
H18A	0.820090	0.570878	0.411921	0.039*	
C19	0.7798 (2)	0.4501 (2)	0.31488 (18)	0.0397 (6)	
H19A	0.726116	0.426313	0.275843	0.048*	
H19B	0.832724	0.453661	0.281844	0.048*	
Si2	0.38858 (4)	0.35475 (4)	0.76190 (4)	0.02756 (14)	
O4	0.31535 (15)	0.39476 (14)	0.71232 (12)	0.0436 (5)	
O5	0.24671 (16)	0.39021 (15)	0.50766 (13)	0.0476 (5)	
H5A	0.270932	0.416753	0.478086	0.071*	
O6	0.24659 (13)	0.58621 (13)	0.60907 (12)	0.0377 (4)	
H6A	0.218187	0.631680	0.636898	0.057*	
C21	0.44350 (17)	0.25345 (17)	0.68828 (16)	0.0304 (5)	
C22	0.4224 (2)	0.16128 (18)	0.68036 (18)	0.0378 (6)	
H22	0.379022	0.148659	0.715194	0.045*	
C23	0.4633 (2)	0.08876 (19)	0.62303 (19)	0.0436 (6)	
H23A	0.447497	0.027283	0.618501	0.052*	
C24	0.5265 (2)	0.10573 (19)	0.57305 (19)	0.0449 (6)	
H24A	0.554762	0.055944	0.534041	0.054*	
C25	0.54968 (19)	0.1957 (2)	0.57896 (18)	0.0422 (6)	
H25	0.593592	0.207410	0.544114	0.051*	
C26	0.50785 (18)	0.26862 (18)	0.63653 (16)	0.0348 (5)	
H26	0.523749	0.329858	0.640360	0.042*	
C27	0.32445 (19)	0.33114 (19)	0.84642 (18)	0.0387 (6)	
C28	0.2401 (2)	0.2642 (2)	0.8096 (2)	0.0577 (9)	
H28A	0.204234	0.259297	0.855041	0.087*	
H28B	0.259618	0.203352	0.776120	0.087*	
H28C	0.202988	0.287556	0.774448	0.087*	
C29	0.3856 (3)	0.2931 (3)	0.8976 (2)	0.0575 (9)	
H29A	0.351084	0.283093	0.941752	0.086*	
H29B	0.437476	0.337161	0.922643	0.086*	
H29C	0.407515	0.234487	0.861376	0.086*	
C30	0.2904 (2)	0.4230 (2)	0.9044 (2)	0.0525 (8)	
H30A	0.255049	0.412275	0.947684	0.079*	
H30B	0.252128	0.448713	0.871907	0.079*	
H30C	0.342533	0.466371	0.930402	0.079*	
C31	0.47679 (17)	0.45200 (16)	0.80992 (15)	0.0291 (5)	
C32	0.56340 (18)	0.43991 (18)	0.83707 (17)	0.0354 (5)	
H32	0.579838	0.379632	0.830362	0.043*	
C33	0.62588 (19)	0.5138 (2)	0.87355 (18)	0.0404 (6)	
H33A	0.684415	0.503583	0.891550	0.048*	
C34	0.6039 (2)	0.60224 (18)	0.88405 (17)	0.0379 (6)	
H34A	0.647405	0.652685	0.908098	0.045*	
C35	0.5180 (2)	0.61643 (19)	0.85918 (19)	0.0430 (6)	
H35	0.501670	0.677095	0.867387	0.052*	
C36	0.45562 (19)	0.54209 (18)	0.82220 (18)	0.0382 (6)	
H36	0.397015	0.552783	0.804794	0.046*	
C37	0.2786 (2)	0.3584 (2)	0.62953 (19)	0.0436 (7)	
H37A	0.214690	0.335535	0.628855	0.052*	
H37B	0.313213	0.305940	0.596312	0.052*	
C38	0.28307 (19)	0.43203 (19)	0.59164 (17)	0.0391 (6)	
H38	0.348020	0.453190	0.591015	0.047*	
C39	0.23143 (18)	0.5132 (2)	0.64052 (18)	0.0385 (6)	
H39A	0.165779	0.494685	0.635841	0.046*	
H39B	0.251804	0.534867	0.700067	0.046*	
Si3	0.10145 (5)	0.64517 (4)	0.24219 (4)	0.02706 (13)	
O7	0.17348 (12)	0.59985 (13)	0.28903 (11)	0.0348 (4)	
O8	0.19471 (13)	0.60054 (14)	0.45611 (11)	0.0372 (4)	
H8	0.209598	0.574760	0.489371	0.056*	
O9	0.33284 (14)	0.46727 (17)	0.41326 (15)	0.0507 (5)	
H9	0.387198	0.486363	0.427564	0.076*	
C41	0.04136 (17)	0.73817 (17)	0.32184 (15)	0.0309 (5)	
C42	0.0499 (3)	0.83191 (19)	0.33473 (18)	0.0476 (7)	
H42	0.088980	0.852977	0.301546	0.057*	
C43	0.0017 (3)	0.8952 (2)	0.3957 (2)	0.0594 (9)	
H43	0.008151	0.958692	0.403303	0.071*	
C44	−0.0550 (2)	0.8665 (2)	0.44492 (18)	0.0498 (8)	
H44	−0.087335	0.910016	0.486410	0.060*	
C45	−0.0645 (2)	0.7748 (2)	0.43374 (17)	0.0415 (6)	
H45	−0.103876	0.754479	0.467266	0.050*	
C46	−0.01643 (17)	0.71138 (18)	0.37312 (16)	0.0335 (5)	
H46	−0.023097	0.648182	0.366456	0.040*	
C47	0.1600 (2)	0.6827 (2)	0.16262 (17)	0.0405 (6)	
C48	0.2005 (3)	0.5974 (3)	0.1003 (2)	0.0603 (9)	
H48A	0.231422	0.614619	0.057931	0.090*	
H48B	0.151794	0.550002	0.073461	0.090*	
H48C	0.244203	0.573318	0.129915	0.090*	
C49	0.0905 (3)	0.7199 (3)	0.1168 (2)	0.0555 (8)	
H49A	0.120607	0.737484	0.074240	0.083*	
H49B	0.064550	0.773960	0.156722	0.083*	
H49C	0.041985	0.672174	0.090233	0.083*	
C50	0.2381 (2)	0.7568 (3)	0.2002 (2)	0.0554 (8)	
H50A	0.265585	0.773040	0.155782	0.083*	
H50B	0.284053	0.732893	0.227787	0.083*	
H50C	0.214364	0.811690	0.240943	0.083*	
C51	0.01679 (16)	0.54692 (16)	0.18823 (14)	0.0262 (4)	
C52	−0.07118 (17)	0.56076 (16)	0.16473 (15)	0.0294 (5)	
H52	−0.088508	0.621743	0.175927	0.035*	
C53	−0.13367 (17)	0.48696 (18)	0.12531 (16)	0.0334 (5)	
H53	−0.193174	0.497712	0.110086	0.040*	
C54	−0.10914 (19)	0.39809 (18)	0.10835 (16)	0.0358 (5)	
H54	−0.152030	0.347683	0.081948	0.043*	
C55	−0.0224 (2)	0.38218 (18)	0.12962 (17)	0.0372 (6)	
H55	−0.005334	0.320920	0.117102	0.045*	
C56	0.03976 (18)	0.45620 (17)	0.16944 (16)	0.0335 (5)	
H56	0.099193	0.444728	0.184171	0.040*	
C57	0.24322 (19)	0.6524 (2)	0.34792 (18)	0.0410 (6)	
H57A	0.221619	0.713264	0.380982	0.049*	
H57B	0.296793	0.662500	0.318260	0.049*	
C58	0.26996 (17)	0.6036 (2)	0.40516 (16)	0.0362 (6)	
H58	0.322430	0.639833	0.442477	0.043*	
C59	0.29851 (19)	0.5084 (2)	0.35744 (18)	0.0421 (6)	
H59A	0.245861	0.469827	0.323703	0.051*	
H59B	0.346014	0.511857	0.319063	0.051*	
Si4	0.37300 (4)	0.86171 (4)	0.77855 (4)	0.02612 (13)	
O10	0.41808 (12)	0.76435 (12)	0.72988 (12)	0.0355 (4)	
O11	0.42184 (15)	0.62098 (15)	0.56691 (12)	0.0446 (5)	
H11	0.369659	0.609246	0.580212	0.067*	
O12	0.55936 (15)	0.49972 (14)	0.56002 (14)	0.0502 (5)	
H12G	0.531541	0.486810	0.513625	0.075*	
C61	0.28623 (16)	0.82591 (18)	0.83973 (15)	0.0306 (5)	
C62	0.21447 (19)	0.8810 (2)	0.87418 (18)	0.0420 (6)	
H62	0.213550	0.940669	0.871311	0.050*	
C63	0.1453 (2)	0.8506 (3)	0.9121 (2)	0.0568 (9)	
H63	0.097149	0.888890	0.934293	0.068*	
C64	0.1463 (2)	0.7649 (3)	0.9175 (2)	0.0657 (11)	
H64	0.098185	0.743520	0.942755	0.079*	
C65	0.2162 (3)	0.7102 (3)	0.8869 (3)	0.0658 (11)	
H65	0.217459	0.651710	0.892325	0.079*	
C66	0.2862 (2)	0.7404 (2)	0.8475 (2)	0.0448 (7)	
H66	0.334209	0.701701	0.825861	0.054*	
C67	0.46068 (17)	0.94979 (17)	0.84805 (16)	0.0324 (5)	
C68	0.4177 (2)	1.0364 (2)	0.9048 (2)	0.0517 (8)	
H68A	0.464725	1.078295	0.942914	0.078*	
H68B	0.371441	1.018913	0.937045	0.078*	
H68C	0.389453	1.067275	0.870717	0.078*	
C69	0.5311 (2)	0.9776 (2)	0.7966 (2)	0.0456 (7)	
H69A	0.580259	1.016954	0.833746	0.068*	
H69B	0.502258	1.011324	0.765365	0.068*	
H69C	0.555961	0.922209	0.757547	0.068*	
C70	0.50708 (19)	0.9049 (2)	0.90383 (17)	0.0368 (6)	
H70A	0.555739	0.947389	0.938920	0.055*	
H70B	0.532727	0.847826	0.868802	0.055*	
H70C	0.462278	0.890909	0.939207	0.055*	
C71	0.31129 (16)	0.89928 (17)	0.70092 (15)	0.0293 (5)	
C72	0.26769 (19)	0.98162 (18)	0.72189 (18)	0.0393 (6)	
H72	0.275644	1.025092	0.777189	0.047*	
C73	0.2134 (2)	1.0019 (2)	0.6648 (2)	0.0454 (7)	
H73	0.183909	1.058003	0.681226	0.054*	
C74	0.2022 (2)	0.9401 (2)	0.58379 (19)	0.0518 (6)	
H74	0.165166	0.953191	0.543945	0.062*	
C75	0.2454 (3)	0.8599 (3)	0.5619 (2)	0.0579 (6)	
H75	0.238062	0.817055	0.506290	0.069*	
C76	0.2994 (3)	0.8399 (3)	0.61900 (19)	0.0542 (7)	
H76	0.329166	0.783946	0.601610	0.065*	
C77	0.49733 (18)	0.73901 (18)	0.68600 (18)	0.0377 (6)	
H77A	0.550851	0.751939	0.725350	0.045*	
H77B	0.506795	0.774269	0.648902	0.045*	
C78	0.48489 (17)	0.63693 (18)	0.63530 (16)	0.0327 (5)	
H78	0.461320	0.603819	0.671419	0.039*	
C79	0.5724 (2)	0.5977 (2)	0.5999 (2)	0.0543 (8)	
H79A	0.592464	0.625071	0.559031	0.065*	
H79B	0.620163	0.613196	0.645119	0.065*	
Si5	0.88653 (4)	0.87436 (5)	0.76889 (4)	0.02719 (13)	
O13	0.97056 (11)	0.80907 (12)	0.72967 (10)	0.0294 (3)	
O14	1.13384 (13)	0.71860 (15)	0.68599 (12)	0.0434 (5)	
H14G	1.116650	0.746340	0.734176	0.065*	
O15	0.93375 (12)	0.61086 (13)	0.54754 (11)	0.0332 (4)	
H15H	0.955030	0.581631	0.501441	0.050*	
C81	0.80756 (16)	0.87774 (18)	0.68306 (15)	0.0304 (5)	
C82	0.7837 (2)	0.9607 (2)	0.67661 (18)	0.0406 (6)	
H82	0.808726	1.017270	0.716296	0.049*	
C83	0.7239 (2)	0.9618 (2)	0.6131 (2)	0.0504 (7)	
H83	0.709110	1.018822	0.609444	0.060*	
C84	0.6861 (2)	0.8806 (2)	0.55566 (19)	0.0464 (7)	
H84	0.644103	0.881682	0.513236	0.056*	
C85	0.7093 (2)	0.7975 (2)	0.55975 (18)	0.0430 (6)	
H85	0.684249	0.741250	0.519510	0.052*	
C86	0.76922 (19)	0.7965 (2)	0.62273 (17)	0.0383 (6)	
H86	0.784675	0.739039	0.624976	0.046*	
C87	0.82943 (18)	0.8190 (2)	0.83793 (17)	0.0377 (6)	
C88	0.8130 (3)	0.7149 (2)	0.7926 (2)	0.0581 (9)	
H88A	0.778574	0.688632	0.827396	0.087*	
H88B	0.871492	0.686853	0.781385	0.087*	
H88C	0.778583	0.702613	0.739800	0.087*	
C89	0.7378 (2)	0.8591 (3)	0.8626 (2)	0.0543 (8)	
H89A	0.708876	0.830164	0.898336	0.081*	
H89B	0.698778	0.847227	0.812366	0.081*	
H89C	0.746883	0.925660	0.892849	0.081*	
C90	0.8891 (2)	0.8373 (3)	0.9172 (2)	0.0664 (11)	
H90A	0.857018	0.812676	0.954548	0.100*	
H90B	0.902198	0.903770	0.945298	0.100*	
H90C	0.946060	0.807130	0.902548	0.100*	
C91	0.93561 (17)	0.99359 (17)	0.83126 (15)	0.0311 (5)	
C92	1.02972 (19)	1.01190 (19)	0.83624 (18)	0.0373 (6)	
H92	1.066826	0.964248	0.806060	0.045*	
C93	1.0704 (2)	1.0973 (2)	0.8838 (2)	0.0484 (7)	
H93	1.134510	1.107589	0.886178	0.058*	
C94	1.0173 (2)	1.1678 (2)	0.92790 (19)	0.0472 (7)	
H94	1.044778	1.226618	0.960749	0.057*	
C95	0.9245 (2)	1.1519 (2)	0.92371 (18)	0.0474 (7)	
H95	0.887887	1.200099	0.953803	0.057*	
C96	0.8840 (2)	1.0666 (2)	0.87622 (17)	0.0400 (6)	
H96	0.819766	1.057238	0.873956	0.048*	
C97	1.01154 (17)	0.79928 (18)	0.65271 (16)	0.0320 (5)	
H97A	1.054265	0.853006	0.660352	0.038*	
H97B	0.964246	0.795930	0.609646	0.038*	
C98	1.06162 (16)	0.71211 (18)	0.62577 (15)	0.0321 (5)	
H98	1.089481	0.705336	0.571805	0.038*	
C99	1.00152 (18)	0.62580 (18)	0.61256 (16)	0.0347 (5)	
H99A	1.039301	0.572088	0.598198	0.042*	
H99B	0.971842	0.631657	0.664878	0.042*	
Si6	0.88453 (5)	0.36705 (5)	0.77432 (4)	0.03133 (15)	
C101	0.94351 (17)	0.2720 (2)	0.69782 (16)	0.0353 (5)	
C102	0.9514 (2)	0.1865 (2)	0.70689 (18)	0.0438 (6)	
H102	0.923495	0.175615	0.751808	0.053*	
C103	0.9984 (2)	0.1184 (2)	0.6525 (2)	0.0522 (8)	
H103	1.002645	0.061270	0.660090	0.063*	
C104	1.0399 (2)	0.1327 (2)	0.5862 (2)	0.0520 (8)	
H104	1.072680	0.085585	0.548693	0.062*	
C105	1.0334 (2)	0.2151 (2)	0.57512 (18)	0.0472 (7)	
H105	1.061626	0.225023	0.529899	0.057*	
C106	0.98572 (19)	0.2840 (2)	0.62976 (17)	0.0403 (6)	
H106	0.981414	0.340505	0.621103	0.048*	
C107	0.96953 (17)	0.44831 (19)	0.85368 (17)	0.0346 (5)	
C108	1.0427 (2)	0.4809 (3)	0.8069 (2)	0.0514 (8)	
H10A	1.085503	0.526451	0.847148	0.077*	
H10B	1.013940	0.508884	0.770655	0.077*	
H10C	1.075384	0.428149	0.773077	0.077*	
C109	0.9237 (2)	0.5338 (2)	0.9105 (2)	0.0490 (7)	
H10D	0.969904	0.577598	0.948039	0.074*	
H10E	0.879168	0.514956	0.943375	0.074*	
H10F	0.893095	0.562960	0.876220	0.074*	
C110	1.01575 (19)	0.4002 (2)	0.90628 (18)	0.0416 (6)	
H11A	1.063828	0.441975	0.942992	0.062*	
H11B	1.042164	0.344527	0.869459	0.062*	
H11C	0.970785	0.383341	0.939964	0.062*	
C111	0.79256 (17)	0.31127 (18)	0.81691 (16)	0.0310 (5)	
C112	0.73553 (19)	0.2395 (2)	0.76089 (17)	0.0391 (6)	
H112	0.745148	0.220398	0.702992	0.047*	
C113	0.66535 (19)	0.1957 (2)	0.78776 (18)	0.0417 (6)	
H113	0.627749	0.147450	0.748520	0.050*	
C114	0.65065 (18)	0.22277 (19)	0.87202 (18)	0.0370 (6)	
H114	0.603082	0.193032	0.890902	0.044*	
C115	0.70565 (18)	0.29340 (18)	0.92853 (17)	0.0348 (5)	
H115	0.695762	0.312102	0.986357	0.042*	
C116	0.77497 (17)	0.33692 (17)	0.90130 (15)	0.0310 (5)	
H116	0.811651	0.385600	0.941049	0.037*	
O16	0.8413 (3)	0.4378 (2)	0.7320 (2)	0.0295 (7)	0.786 (3)
O17	0.66290 (14)	0.36915 (16)	0.56641 (14)	0.0288 (5)	0.786 (3)
H17	0.641130	0.420659	0.577089	0.043*	0.786 (3)
O18	0.78044 (14)	0.51423 (18)	0.56184 (14)	0.0305 (5)	0.786 (3)
H18	0.823683	0.552541	0.564675	0.046*	0.786 (3)
C117	0.7537 (2)	0.4333 (2)	0.69416 (19)	0.0298 (6)	0.786 (3)
H11D	0.734784	0.496459	0.705123	0.036*	0.786 (3)
H11E	0.709390	0.401800	0.718637	0.036*	0.786 (3)
C118	0.7543 (2)	0.3820 (3)	0.6018 (2)	0.0270 (7)	0.786 (3)
H118	0.776543	0.319758	0.592664	0.032*	0.786 (3)
C119	0.8157 (2)	0.4282 (2)	0.5570 (2)	0.0302 (7)	0.786 (3)
H11F	0.877921	0.439365	0.583011	0.036*	0.786 (3)
H11G	0.818599	0.387612	0.497904	0.036*	0.786 (3)
O216	0.8405 (12)	0.4055 (9)	0.7100 (8)	0.037 (3)	0.214 (3)
O217	0.7036 (9)	0.3927 (8)	0.5286 (7)	0.059 (3)	0.214 (3)
H217	0.709944	0.423297	0.497519	0.088*	0.214 (3)
O218	0.7677 (7)	0.5777 (8)	0.5857 (6)	0.042 (2)	0.214 (3)
H218	0.819255	0.584536	0.568471	0.062*	0.214 (3)
C217	0.7670 (8)	0.3719 (9)	0.6518 (8)	0.0298 (6)	0.214 (3)
H21A	0.709107	0.383325	0.680419	0.036*	0.214 (3)
H21B	0.769474	0.304764	0.622210	0.036*	0.214 (3)
C218	0.7723 (11)	0.4207 (11)	0.5912 (9)	0.037 (2)	0.214 (3)
H21C	0.831469	0.407584	0.563820	0.044*	0.214 (3)
C219	0.7737 (8)	0.5228 (8)	0.6391 (8)	0.042 (3)	0.214 (3)
H21D	0.722256	0.535594	0.676339	0.050*	0.214 (3)
H21E	0.830514	0.542541	0.674459	0.050*	0.214 (3)
Si7	0.14019 (4)	0.15762 (4)	0.25987 (4)	0.02807 (14)	
O19	0.09203 (13)	0.25157 (13)	0.31485 (14)	0.0435 (5)	
O20	0.05865 (16)	0.38991 (17)	0.48986 (13)	0.0511 (5)	
H20	0.113962	0.383782	0.480665	0.077*	
O21	0.02256 (13)	0.52176 (12)	0.41127 (12)	0.0382 (4)	
H21	0.074086	0.548934	0.427127	0.046*	
C127	0.05758 (18)	0.06869 (19)	0.18395 (17)	0.0358 (6)	
C128	0.1066 (3)	−0.0140 (2)	0.1288 (2)	0.0594 (9)	
H12A	0.063001	−0.057147	0.087394	0.089*	
H12B	0.133694	−0.044959	0.163322	0.089*	
H12C	0.154357	0.007156	0.100272	0.089*	
C129	0.0134 (2)	0.1143 (3)	0.1276 (2)	0.0578 (9)	
H12D	−0.029185	0.069362	0.086496	0.087*	
H12E	0.060600	0.135680	0.098673	0.087*	
H12F	−0.019389	0.166813	0.161691	0.087*	
C130	−0.0180 (2)	0.0340 (2)	0.2272 (2)	0.0431 (7)	
H13A	−0.058291	−0.012620	0.185087	0.065*	
H13B	−0.053045	0.085646	0.259206	0.065*	
H13C	0.009141	0.006716	0.264750	0.065*	
C131	0.22497 (17)	0.20001 (17)	0.20134 (16)	0.0308 (5)	
C132	0.30364 (19)	0.1546 (2)	0.17488 (19)	0.0408 (6)	
H132	0.314409	0.101330	0.187446	0.049*	
C133	0.3665 (2)	0.1848 (2)	0.1309 (2)	0.0478 (7)	
H133	0.419462	0.152312	0.113448	0.057*	
C134	0.3523 (2)	0.2621 (2)	0.1122 (2)	0.0487 (7)	
H134	0.395430	0.283292	0.082189	0.058*	
C135	0.2751 (2)	0.3083 (2)	0.1376 (2)	0.0562 (8)	
H135	0.264641	0.361187	0.124346	0.067*	
C136	0.2125 (2)	0.2782 (2)	0.1822 (2)	0.0450 (7)	
H136	0.160148	0.311565	0.200119	0.054*	
C137	0.01100 (17)	0.26565 (18)	0.35746 (17)	0.0340 (5)	
H13D	0.007333	0.226802	0.391950	0.041*	
H13E	−0.041597	0.247194	0.316721	0.041*	
C138	0.00733 (18)	0.36590 (18)	0.41259 (16)	0.0349 (5)	
H138	−0.057515	0.375533	0.426684	0.042*	
C139	0.0323 (2)	0.42710 (18)	0.36523 (17)	0.0388 (6)	
H13F	0.096065	0.418716	0.349340	0.047*	
H13G	−0.006603	0.408044	0.313342	0.047*	
C121	0.2032 (12)	0.1180 (7)	0.3340 (7)	0.0296 (15)	0.646 (5)
C122	0.1867 (4)	0.0344 (3)	0.3427 (3)	0.0510 (14)	0.646 (5)
H122	0.139466	−0.007006	0.309054	0.061*	0.646 (5)
C123	0.2374 (5)	0.0084 (5)	0.3994 (4)	0.0653 (18)	0.646 (5)
H123	0.224453	−0.050264	0.403104	0.078*	0.646 (5)
C124	0.3037 (10)	0.0650 (8)	0.4485 (7)	0.060 (4)	0.646 (5)
H124	0.337438	0.047380	0.487500	0.072*	0.646 (5)
C125	0.3223 (4)	0.1492 (4)	0.4416 (3)	0.0543 (15)	0.646 (5)
H125	0.370341	0.189244	0.475452	0.065*	0.646 (5)
C126	0.2716 (3)	0.1771 (3)	0.3855 (3)	0.0478 (12)	0.646 (5)
H126	0.284282	0.236252	0.382786	0.057*	0.646 (5)
C161	0.199 (2)	0.1027 (15)	0.3303 (12)	0.0296 (15)	0.354 (5)
C162	0.2437 (6)	0.0217 (5)	0.2988 (5)	0.0353 (19)	0.354 (5)
H162	0.238611	−0.012324	0.242120	0.042*	0.354 (5)
C163	0.2902 (6)	−0.0109 (5)	0.3527 (5)	0.042 (2)	0.354 (5)
H163	0.313109	−0.068409	0.332992	0.050*	0.354 (5)
C164	0.2983 (19)	0.0375 (12)	0.4362 (11)	0.047 (4)	0.354 (5)
H164	0.332614	0.015460	0.471806	0.057*	0.354 (5)
C165	0.2568 (8)	0.1176 (6)	0.4678 (5)	0.049 (3)	0.354 (5)
H165	0.258613	0.149093	0.524925	0.059*	0.354 (5)
C166	0.2088 (5)	0.1522 (4)	0.4154 (4)	0.0317 (18)	0.354 (5)
H166	0.180522	0.206389	0.439185	0.038*	0.354 (5)
Si8	0.63688 (4)	0.09100 (4)	0.21825 (4)	0.02685 (13)	
C141	0.70785 (16)	0.08392 (17)	0.30793 (15)	0.0292 (5)	
C142	0.6985 (2)	0.00816 (19)	0.33224 (17)	0.0405 (6)	
H142	0.659140	−0.043213	0.300108	0.049*	
C143	0.7454 (3)	0.0058 (2)	0.40232 (19)	0.0515 (8)	
H143	0.737393	−0.046713	0.417673	0.062*	
C144	0.8033 (2)	0.0790 (2)	0.44978 (18)	0.0449 (7)	
H144	0.835157	0.077344	0.497847	0.054*	
C145	0.81439 (19)	0.1541 (2)	0.42694 (17)	0.0406 (6)	
H145	0.854004	0.205100	0.459502	0.049*	
C146	0.76814 (19)	0.15643 (19)	0.35645 (17)	0.0369 (6)	
H146	0.777799	0.208647	0.340892	0.044*	
C147	0.70145 (18)	0.12017 (18)	0.13638 (16)	0.0342 (5)	
C148	0.7673 (3)	0.2053 (2)	0.1758 (2)	0.0549 (8)	
H14A	0.796300	0.221106	0.131812	0.082*	
H14B	0.813845	0.192121	0.210881	0.082*	
H14C	0.733749	0.257208	0.209969	0.082*	
C149	0.7550 (2)	0.0395 (2)	0.0848 (2)	0.0473 (7)	
H14D	0.789923	0.057203	0.044784	0.071*	
H14E	0.712933	−0.013532	0.054855	0.071*	
H14F	0.796649	0.023084	0.121854	0.071*	
C150	0.6320 (2)	0.1409 (2)	0.0787 (2)	0.0492 (7)	
H15A	0.663621	0.155143	0.035144	0.074*	
H15B	0.598558	0.193696	0.111107	0.074*	
H15C	0.589445	0.086993	0.053048	0.074*	
C151	0.56551 (16)	−0.01935 (16)	0.16614 (15)	0.0274 (5)	
C152	0.47140 (18)	−0.01715 (18)	0.16254 (19)	0.0380 (6)	
H152	0.444641	0.039197	0.190579	0.046*	
C153	0.41529 (19)	−0.0956 (2)	0.1188 (2)	0.0446 (7)	
H153	0.351223	−0.092273	0.117903	0.054*	
C154	0.4525 (2)	−0.17712 (19)	0.07725 (18)	0.0395 (6)	
H154	0.414393	−0.230106	0.046364	0.047*	
C155	0.54551 (19)	−0.18232 (17)	0.08026 (16)	0.0338 (5)	
H155	0.571333	−0.239139	0.051943	0.041*	
C156	0.60149 (17)	−0.10464 (17)	0.12464 (15)	0.0298 (5)	
H156	0.665330	−0.109342	0.126918	0.036*	
O22	0.5705 (3)	0.1764 (2)	0.2526 (3)	0.0296 (7)	0.876 (4)
C157	0.5246 (2)	0.1996 (3)	0.3281 (3)	0.0320 (9)	0.876 (4)
H15D	0.546561	0.163298	0.360853	0.038*	0.876 (4)
H15E	0.458639	0.184425	0.315607	0.038*	0.876 (4)
O23	0.63645 (13)	0.32237 (14)	0.40090 (12)	0.0327 (5)	0.876 (4)
H23	0.645294	0.331077	0.451628	0.049*	0.876 (4)
C158	0.54175 (18)	0.30091 (19)	0.37800 (17)	0.0282 (6)	0.876 (4)
H158	0.507180	0.316499	0.429855	0.034*	0.876 (4)
C159	0.5134 (2)	0.3615 (2)	0.3308 (2)	0.0318 (6)	0.876 (4)
H15F	0.552705	0.352730	0.283249	0.038*	0.876 (4)
H15G	0.449929	0.343711	0.308474	0.038*	0.876 (4)
O24	0.5210 (3)	0.4573 (2)	0.3857 (2)	0.0364 (7)	0.876 (4)
H24	0.574248	0.479258	0.386301	0.055*	0.876 (4)
O32	0.555 (2)	0.168 (2)	0.260 (2)	0.0296 (7)	0.124 (4)
C257	0.501 (2)	0.1968 (18)	0.332 (2)	0.035 (4)	0.124 (4)
H25A	0.535481	0.189392	0.378560	0.042*	0.124 (4)
H25B	0.445436	0.154660	0.319806	0.042*	0.124 (4)
O33	0.4403 (12)	0.3227 (13)	0.4425 (10)	0.055 (5)	0.124 (4)
H33	0.452014	0.283000	0.463143	0.082*	0.124 (4)
C258	0.4715 (14)	0.2950 (13)	0.3599 (12)	0.042 (4)	0.124 (4)
H258	0.420430	0.297631	0.321602	0.050*	0.124 (4)
C259	0.5449 (16)	0.3628 (15)	0.3601 (18)	0.044 (4)	0.124 (4)
H25C	0.588315	0.372516	0.407838	0.053*	0.124 (4)
H25D	0.578078	0.337877	0.308408	0.053*	0.124 (4)
O34	0.511 (3)	0.4503 (18)	0.366 (2)	0.0364 (7)	0.124 (4)
H34	0.5742 (8)	0.4793 (7)	0.3863 (18)	0.055*	0.124 (4)

### 3-[(tert-Butyldiphenylsilyl)oxy]propane-1,2-diol (1) Atomic displacement parameters (Å^2^)

**Table:**

	*U*^11^	*U*^22^	*U*^33^	*U*^12^	*U*^13^	*U*^23^
Si1	0.0252 (3)	0.0226 (3)	0.0218 (3)	−0.0013 (2)	−0.0012 (2)	0.0091 (2)
O1	0.0287 (8)	0.0286 (8)	0.0296 (8)	−0.0026 (7)	−0.0075 (6)	0.0145 (7)
O2	0.0375 (9)	0.0414 (10)	0.0309 (9)	−0.0001 (8)	−0.0046 (7)	0.0184 (8)
O3	0.0368 (10)	0.0467 (11)	0.0578 (12)	0.0102 (9)	0.0035 (9)	0.0320 (10)
C1	0.0301 (12)	0.0265 (11)	0.0254 (11)	−0.0050 (9)	−0.0010 (9)	0.0070 (9)
C2	0.0581 (18)	0.0277 (13)	0.0384 (14)	0.0026 (12)	0.0136 (12)	0.0112 (11)
C3	0.079 (2)	0.0292 (14)	0.0544 (19)	0.0095 (14)	0.0219 (17)	0.0095 (13)
C4	0.0601 (19)	0.0388 (15)	0.0398 (15)	0.0011 (13)	0.0174 (14)	−0.0032 (12)
C5	0.0403 (14)	0.0460 (16)	0.0271 (12)	−0.0098 (12)	0.0034 (10)	0.0055 (11)
C6	0.0315 (12)	0.0358 (13)	0.0268 (12)	−0.0049 (10)	−0.0008 (9)	0.0101 (10)
C7	0.0272 (11)	0.0251 (11)	0.0278 (11)	−0.0020 (9)	−0.0021 (9)	0.0096 (9)
C8	0.0391 (14)	0.0245 (12)	0.0359 (13)	−0.0007 (10)	−0.0006 (10)	0.0056 (10)
C9	0.0341 (13)	0.0344 (13)	0.0363 (13)	−0.0088 (10)	−0.0013 (10)	0.0141 (11)
C10	0.0312 (12)	0.0377 (13)	0.0309 (12)	−0.0026 (10)	−0.0079 (10)	0.0141 (10)
C11	0.0243 (11)	0.0239 (11)	0.0287 (11)	0.0012 (8)	−0.0011 (8)	0.0119 (9)
C12	0.0349 (13)	0.0297 (12)	0.0284 (12)	−0.0032 (10)	−0.0014 (9)	0.0111 (10)
C13	0.0350 (13)	0.0312 (12)	0.0402 (14)	−0.0082 (10)	−0.0026 (10)	0.0136 (11)
C14	0.0317 (13)	0.0356 (13)	0.0446 (15)	−0.0011 (10)	0.0043 (10)	0.0216 (12)
C15	0.0347 (13)	0.0345 (13)	0.0323 (12)	0.0021 (10)	0.0060 (10)	0.0157 (10)
C16	0.0291 (11)	0.0262 (11)	0.0287 (12)	−0.0002 (9)	−0.0002 (9)	0.0102 (9)
C17	0.0280 (12)	0.0324 (12)	0.0327 (12)	−0.0050 (9)	−0.0072 (9)	0.0148 (10)
C18	0.0288 (12)	0.0378 (13)	0.0339 (12)	−0.0021 (10)	−0.0067 (9)	0.0188 (11)
C19	0.0411 (14)	0.0434 (15)	0.0422 (14)	0.0104 (12)	0.0014 (11)	0.0236 (12)
Si2	0.0291 (3)	0.0233 (3)	0.0289 (3)	0.0006 (2)	−0.0046 (2)	0.0084 (3)
O4	0.0502 (12)	0.0376 (10)	0.0382 (10)	0.0137 (9)	−0.0146 (9)	0.0076 (8)
O5	0.0568 (13)	0.0442 (12)	0.0382 (11)	0.0037 (9)	−0.0114 (9)	0.0114 (9)
O6	0.0386 (10)	0.0348 (10)	0.0392 (10)	0.0122 (8)	−0.0008 (8)	0.0116 (8)
C21	0.0298 (12)	0.0264 (11)	0.0330 (12)	0.0011 (9)	−0.0086 (9)	0.0090 (9)
C22	0.0453 (15)	0.0277 (12)	0.0388 (14)	−0.0009 (11)	−0.0081 (11)	0.0112 (11)
C23	0.0522 (13)	0.0281 (11)	0.0435 (13)	0.0055 (10)	−0.0127 (10)	0.0054 (10)
C24	0.0496 (13)	0.0307 (11)	0.0436 (13)	0.0110 (10)	−0.0149 (10)	0.0005 (10)
C25	0.0340 (14)	0.0463 (16)	0.0382 (14)	0.0052 (12)	−0.0010 (11)	0.0052 (12)
C26	0.0339 (13)	0.0291 (12)	0.0361 (13)	0.0002 (10)	−0.0020 (10)	0.0059 (10)
C27	0.0383 (14)	0.0378 (14)	0.0382 (14)	−0.0053 (11)	0.0030 (11)	0.0125 (11)
C28	0.0465 (18)	0.0452 (18)	0.069 (2)	−0.0146 (14)	0.0125 (16)	0.0076 (16)
C29	0.070 (2)	0.066 (2)	0.0472 (18)	−0.0041 (18)	0.0014 (16)	0.0348 (17)
C30	0.0455 (17)	0.0500 (18)	0.0480 (17)	−0.0069 (14)	0.0114 (13)	0.0016 (14)
C31	0.0336 (12)	0.0251 (11)	0.0291 (12)	0.0019 (9)	−0.0009 (9)	0.0106 (9)
C32	0.0342 (13)	0.0279 (12)	0.0409 (14)	0.0030 (10)	−0.0031 (10)	0.0086 (11)
C33	0.0321 (13)	0.0408 (15)	0.0425 (15)	−0.0002 (11)	−0.0034 (11)	0.0088 (12)
C34	0.0447 (15)	0.0299 (13)	0.0325 (13)	−0.0110 (11)	0.0006 (11)	0.0052 (10)
C35	0.0545 (17)	0.0252 (13)	0.0484 (16)	−0.0005 (11)	−0.0031 (13)	0.0128 (12)
C36	0.0404 (14)	0.0257 (12)	0.0463 (15)	0.0029 (10)	−0.0068 (11)	0.0107 (11)
C37	0.0422 (15)	0.0357 (14)	0.0474 (16)	0.0002 (11)	−0.0196 (12)	0.0102 (12)
C38	0.0373 (14)	0.0386 (14)	0.0377 (14)	0.0062 (11)	−0.0099 (11)	0.0096 (11)
C39	0.0335 (13)	0.0433 (15)	0.0401 (14)	0.0084 (11)	−0.0019 (11)	0.0162 (12)
Si3	0.0289 (3)	0.0253 (3)	0.0252 (3)	−0.0013 (2)	−0.0016 (2)	0.0076 (2)
O7	0.0321 (9)	0.0362 (10)	0.0317 (9)	0.0017 (7)	−0.0091 (7)	0.0078 (8)
O8	0.0357 (9)	0.0470 (11)	0.0288 (9)	0.0105 (8)	0.0011 (7)	0.0126 (8)
O9	0.0351 (10)	0.0614 (14)	0.0621 (14)	0.0131 (10)	0.0027 (9)	0.0289 (11)
C41	0.0366 (13)	0.0262 (11)	0.0270 (11)	0.0003 (9)	−0.0063 (9)	0.0068 (9)
C42	0.079 (2)	0.0293 (14)	0.0337 (14)	0.0037 (14)	0.0031 (14)	0.0099 (11)
C43	0.105 (3)	0.0269 (14)	0.0429 (17)	0.0167 (16)	0.0013 (17)	0.0072 (13)
C44	0.065 (2)	0.0452 (16)	0.0302 (14)	0.0217 (15)	−0.0054 (13)	0.0007 (12)
C45	0.0395 (14)	0.0478 (16)	0.0317 (13)	0.0085 (12)	−0.0016 (11)	0.0072 (12)
C46	0.0323 (12)	0.0323 (13)	0.0322 (12)	0.0023 (10)	−0.0007 (10)	0.0075 (10)
C47	0.0448 (15)	0.0400 (15)	0.0348 (14)	−0.0083 (12)	0.0030 (11)	0.0126 (12)
C48	0.068 (2)	0.055 (2)	0.0510 (19)	−0.0026 (17)	0.0263 (17)	0.0110 (16)
C49	0.066 (2)	0.070 (2)	0.0388 (16)	−0.0095 (17)	−0.0042 (14)	0.0326 (16)
C50	0.0506 (18)	0.058 (2)	0.061 (2)	−0.0204 (15)	0.0007 (15)	0.0283 (17)
C51	0.0309 (11)	0.0265 (11)	0.0222 (10)	0.0003 (9)	0.0005 (8)	0.0104 (9)
C52	0.0324 (12)	0.0254 (11)	0.0311 (12)	0.0017 (9)	−0.0001 (9)	0.0111 (9)
C53	0.0313 (12)	0.0350 (13)	0.0328 (13)	−0.0033 (10)	−0.0034 (10)	0.0120 (10)
C54	0.0443 (14)	0.0310 (13)	0.0292 (12)	−0.0086 (11)	−0.0045 (10)	0.0092 (10)
C55	0.0533 (16)	0.0230 (12)	0.0349 (13)	0.0004 (11)	−0.0038 (11)	0.0107 (10)
C56	0.0373 (13)	0.0278 (12)	0.0348 (13)	0.0048 (10)	−0.0048 (10)	0.0108 (10)
C57	0.0357 (14)	0.0471 (16)	0.0395 (14)	−0.0077 (12)	−0.0102 (11)	0.0169 (12)
C58	0.0284 (12)	0.0471 (15)	0.0333 (13)	−0.0040 (11)	−0.0053 (10)	0.0159 (11)
C59	0.0323 (13)	0.0568 (18)	0.0431 (15)	0.0142 (12)	0.0114 (11)	0.0229 (13)
Si4	0.0249 (3)	0.0245 (3)	0.0293 (3)	0.0035 (2)	0.0011 (2)	0.0099 (2)
O10	0.0311 (9)	0.0285 (9)	0.0424 (10)	0.0061 (7)	0.0067 (7)	0.0066 (8)
O11	0.0466 (11)	0.0474 (12)	0.0350 (10)	0.0095 (9)	−0.0056 (8)	0.0090 (9)
O12	0.0432 (11)	0.0382 (11)	0.0536 (13)	0.0126 (9)	−0.0057 (9)	−0.0031 (9)
C61	0.0282 (12)	0.0368 (13)	0.0282 (12)	0.0000 (10)	−0.0038 (9)	0.0140 (10)
C62	0.0364 (14)	0.0512 (17)	0.0374 (14)	0.0062 (12)	0.0084 (11)	0.0137 (12)
C63	0.0418 (17)	0.086 (3)	0.0435 (17)	0.0056 (16)	0.0129 (13)	0.0236 (17)
C64	0.0438 (18)	0.116 (3)	0.057 (2)	−0.007 (2)	0.0062 (15)	0.057 (2)
C65	0.057 (2)	0.076 (3)	0.088 (3)	−0.0177 (19)	−0.0115 (19)	0.063 (2)
C66	0.0387 (15)	0.0477 (16)	0.0567 (18)	0.0007 (12)	−0.0031 (12)	0.0302 (14)
C67	0.0313 (12)	0.0276 (12)	0.0379 (13)	0.0023 (9)	−0.0057 (10)	0.0118 (10)
C68	0.0501 (17)	0.0327 (14)	0.0554 (18)	0.0102 (12)	−0.0214 (14)	−0.0037 (13)
C69	0.0456 (16)	0.0416 (16)	0.0539 (18)	−0.0143 (13)	−0.0089 (13)	0.0255 (14)
C70	0.0335 (13)	0.0389 (14)	0.0396 (14)	0.0041 (11)	−0.0049 (10)	0.0163 (11)
C71	0.0270 (11)	0.0329 (12)	0.0317 (12)	0.0011 (9)	0.0030 (9)	0.0159 (10)
C72	0.0439 (15)	0.0268 (12)	0.0442 (15)	−0.0015 (11)	−0.0118 (12)	0.0105 (11)
C73	0.0452 (16)	0.0331 (14)	0.0593 (18)	0.0000 (12)	−0.0157 (13)	0.0201 (13)
C74	0.0634 (15)	0.0634 (15)	0.0342 (11)	0.0145 (12)	−0.0040 (11)	0.0236 (11)
C75	0.0694 (14)	0.0685 (14)	0.0336 (10)	0.0228 (12)	−0.0017 (10)	0.0138 (10)
C76	0.0658 (15)	0.0639 (15)	0.0313 (11)	0.0271 (13)	0.0032 (11)	0.0123 (11)
C77	0.0308 (13)	0.0342 (13)	0.0418 (14)	0.0039 (10)	0.0050 (11)	0.0057 (11)
C78	0.0293 (12)	0.0333 (13)	0.0321 (12)	0.0047 (10)	−0.0002 (9)	0.0075 (10)
C79	0.0390 (16)	0.0395 (16)	0.067 (2)	0.0071 (12)	0.0073 (14)	−0.0022 (15)
Si5	0.0245 (3)	0.0329 (3)	0.0247 (3)	0.0080 (2)	0.0042 (2)	0.0101 (3)
O13	0.0308 (8)	0.0319 (9)	0.0267 (8)	0.0106 (7)	0.0072 (6)	0.0105 (7)
O14	0.0331 (9)	0.0502 (12)	0.0344 (10)	0.0140 (8)	−0.0044 (7)	−0.0010 (9)
O15	0.0284 (8)	0.0355 (9)	0.0321 (9)	0.0044 (7)	0.0017 (7)	0.0074 (7)
C81	0.0240 (11)	0.0388 (13)	0.0296 (12)	0.0059 (9)	0.0039 (9)	0.0133 (10)
C82	0.0433 (15)	0.0383 (14)	0.0385 (14)	0.0045 (11)	−0.0085 (11)	0.0125 (12)
C83	0.0512 (17)	0.0504 (18)	0.0544 (18)	0.0046 (14)	−0.0135 (14)	0.0258 (15)
C84	0.0406 (15)	0.0586 (19)	0.0429 (16)	−0.0057 (13)	−0.0146 (12)	0.0242 (14)
C85	0.0422 (15)	0.0476 (16)	0.0369 (14)	−0.0097 (12)	−0.0082 (11)	0.0151 (12)
C86	0.0406 (14)	0.0391 (14)	0.0359 (14)	0.0019 (11)	−0.0009 (11)	0.0146 (11)
C87	0.0296 (12)	0.0538 (16)	0.0360 (13)	0.0066 (11)	0.0073 (10)	0.0228 (12)
C88	0.067 (2)	0.0526 (19)	0.064 (2)	0.0015 (16)	0.0248 (17)	0.0313 (17)
C89	0.0387 (16)	0.072 (2)	0.0563 (19)	0.0088 (15)	0.0206 (14)	0.0258 (17)
C90	0.0514 (19)	0.114 (3)	0.054 (2)	−0.014 (2)	−0.0077 (15)	0.059 (2)
C91	0.0370 (13)	0.0322 (12)	0.0244 (11)	0.0097 (10)	0.0018 (9)	0.0095 (9)
C92	0.0370 (14)	0.0321 (13)	0.0411 (14)	0.0069 (11)	0.0035 (11)	0.0105 (11)
C93	0.0478 (17)	0.0412 (16)	0.0542 (18)	−0.0026 (13)	−0.0010 (14)	0.0157 (14)
C94	0.072 (2)	0.0296 (14)	0.0375 (15)	0.0048 (13)	−0.0025 (14)	0.0096 (12)
C95	0.067 (2)	0.0389 (15)	0.0343 (14)	0.0212 (14)	0.0048 (13)	0.0084 (12)
C96	0.0440 (15)	0.0420 (15)	0.0319 (13)	0.0142 (12)	0.0044 (11)	0.0091 (11)
C97	0.0320 (12)	0.0336 (13)	0.0294 (12)	0.0057 (10)	0.0096 (9)	0.0090 (10)
C98	0.0268 (11)	0.0354 (13)	0.0289 (12)	0.0064 (9)	0.0007 (9)	0.0050 (10)
C99	0.0353 (13)	0.0327 (13)	0.0339 (13)	0.0087 (10)	−0.0007 (10)	0.0089 (10)
Si6	0.0268 (3)	0.0425 (4)	0.0289 (3)	−0.0025 (3)	−0.0046 (2)	0.0189 (3)
C101	0.0301 (12)	0.0456 (15)	0.0301 (12)	−0.0013 (11)	−0.0060 (10)	0.0147 (11)
C102	0.0453 (16)	0.0497 (17)	0.0373 (14)	−0.0021 (13)	−0.0018 (12)	0.0176 (13)
C103	0.0563 (19)	0.0453 (17)	0.0553 (19)	0.0060 (14)	−0.0012 (15)	0.0184 (15)
C104	0.0551 (19)	0.0566 (19)	0.0394 (16)	0.0137 (15)	0.0004 (13)	0.0100 (14)
C105	0.0438 (16)	0.069 (2)	0.0313 (14)	0.0101 (14)	0.0016 (11)	0.0198 (14)
C106	0.0359 (14)	0.0545 (17)	0.0350 (14)	0.0041 (12)	−0.0033 (10)	0.0219 (13)
C107	0.0294 (12)	0.0383 (14)	0.0376 (14)	−0.0032 (10)	−0.0044 (10)	0.0167 (11)
C108	0.0403 (16)	0.059 (2)	0.060 (2)	−0.0150 (14)	−0.0040 (14)	0.0302 (16)
C109	0.0530 (18)	0.0352 (15)	0.0546 (18)	−0.0016 (13)	−0.0023 (14)	0.0120 (13)
C110	0.0326 (13)	0.0540 (17)	0.0365 (14)	−0.0010 (12)	−0.0092 (11)	0.0156 (13)
C111	0.0272 (11)	0.0375 (13)	0.0298 (12)	0.0000 (10)	−0.0025 (9)	0.0145 (10)
C112	0.0355 (13)	0.0501 (16)	0.0290 (13)	−0.0060 (12)	−0.0027 (10)	0.0125 (12)
C113	0.0363 (14)	0.0406 (15)	0.0429 (15)	−0.0077 (11)	−0.0058 (11)	0.0105 (12)
C114	0.0316 (13)	0.0334 (13)	0.0484 (15)	−0.0007 (10)	0.0059 (11)	0.0178 (12)
C115	0.0383 (13)	0.0350 (13)	0.0324 (13)	0.0075 (10)	0.0049 (10)	0.0130 (11)
C116	0.0311 (12)	0.0314 (12)	0.0290 (12)	0.0014 (9)	−0.0024 (9)	0.0095 (10)
O16	0.0290 (12)	0.0369 (19)	0.0264 (16)	0.0022 (15)	−0.0042 (13)	0.0165 (14)
O17	0.0252 (11)	0.0349 (12)	0.0261 (11)	−0.0027 (8)	−0.0015 (8)	0.0115 (9)
O18	0.0231 (10)	0.0377 (14)	0.0350 (12)	0.0005 (9)	0.0017 (8)	0.0186 (11)
C117	0.0289 (14)	0.0359 (15)	0.0274 (14)	0.0055 (12)	−0.0004 (11)	0.0146 (11)
C118	0.0261 (15)	0.0303 (16)	0.0289 (16)	0.0027 (12)	0.0033 (11)	0.0156 (13)
C119	0.0275 (15)	0.0389 (17)	0.0289 (15)	0.0058 (12)	0.0020 (12)	0.0175 (13)
O216	0.043 (5)	0.030 (6)	0.030 (6)	−0.001 (5)	−0.007 (4)	0.002 (4)
O217	0.076 (7)	0.051 (6)	0.045 (6)	0.005 (5)	−0.025 (5)	0.014 (5)
O218	0.047 (5)	0.045 (6)	0.043 (5)	0.006 (4)	−0.002 (4)	0.028 (5)
C217	0.0289 (14)	0.0359 (15)	0.0274 (14)	0.0055 (12)	−0.0004 (11)	0.0146 (11)
C218	0.039 (5)	0.045 (5)	0.028 (4)	−0.002 (5)	−0.011 (4)	0.017 (4)
C219	0.052 (6)	0.040 (5)	0.031 (5)	0.004 (5)	−0.006 (4)	0.012 (4)
Si7	0.0257 (3)	0.0223 (3)	0.0379 (4)	0.0030 (2)	0.0022 (3)	0.0128 (3)
O19	0.0361 (10)	0.0295 (9)	0.0660 (13)	0.0082 (8)	0.0211 (9)	0.0163 (9)
O20	0.0547 (13)	0.0593 (13)	0.0384 (11)	0.0080 (11)	−0.0042 (9)	0.0165 (10)
O21	0.0386 (10)	0.0292 (9)	0.0439 (10)	0.0045 (7)	0.0068 (8)	0.0091 (8)
C127	0.0348 (13)	0.0359 (13)	0.0397 (14)	−0.0042 (10)	−0.0083 (11)	0.0191 (11)
C128	0.066 (2)	0.0455 (18)	0.0492 (18)	−0.0072 (16)	−0.0015 (16)	−0.0029 (14)
C129	0.0426 (17)	0.079 (2)	0.070 (2)	−0.0185 (16)	−0.0223 (15)	0.054 (2)
C130	0.0406 (15)	0.0377 (15)	0.0541 (17)	−0.0125 (12)	−0.0084 (12)	0.0232 (13)
C131	0.0288 (12)	0.0255 (11)	0.0385 (13)	0.0027 (9)	0.0012 (10)	0.0120 (10)
C132	0.0369 (14)	0.0451 (15)	0.0479 (16)	0.0125 (12)	0.0097 (12)	0.0239 (13)
C133	0.0359 (15)	0.0602 (19)	0.0523 (17)	0.0120 (13)	0.0145 (12)	0.0242 (15)
C134	0.0472 (17)	0.0544 (18)	0.0490 (17)	−0.0085 (14)	0.0110 (13)	0.0250 (15)
C135	0.058 (2)	0.0473 (18)	0.078 (2)	0.0012 (15)	0.0125 (17)	0.0401 (18)
C136	0.0400 (15)	0.0345 (14)	0.070 (2)	0.0073 (11)	0.0125 (14)	0.0294 (14)
C137	0.0306 (12)	0.0330 (13)	0.0381 (13)	−0.0017 (10)	0.0037 (10)	0.0129 (11)
C138	0.0314 (12)	0.0363 (13)	0.0368 (13)	0.0013 (10)	0.0029 (10)	0.0129 (11)
C139	0.0473 (15)	0.0301 (13)	0.0387 (14)	0.0090 (11)	0.0072 (11)	0.0108 (11)
C121	0.029 (2)	0.027 (4)	0.0323 (15)	0.001 (3)	−0.0006 (12)	0.0106 (19)
C122	0.059 (3)	0.044 (3)	0.053 (3)	−0.010 (2)	−0.023 (3)	0.026 (2)
C123	0.078 (4)	0.074 (4)	0.062 (4)	−0.007 (3)	−0.020 (4)	0.048 (3)
C124	0.061 (6)	0.088 (9)	0.040 (4)	0.021 (7)	−0.006 (3)	0.031 (5)
C125	0.042 (3)	0.065 (3)	0.039 (2)	0.003 (2)	−0.012 (2)	−0.001 (2)
C126	0.043 (3)	0.043 (3)	0.047 (3)	−0.004 (2)	−0.006 (2)	0.005 (2)
C161	0.029 (2)	0.027 (4)	0.0323 (15)	0.001 (3)	−0.0006 (12)	0.0106 (19)
C162	0.045 (4)	0.022 (3)	0.035 (4)	0.011 (3)	−0.011 (3)	0.005 (3)
C163	0.059 (5)	0.016 (3)	0.046 (5)	0.008 (3)	−0.017 (4)	0.005 (3)
C164	0.052 (8)	0.037 (7)	0.054 (9)	0.003 (6)	−0.020 (6)	0.019 (6)
C165	0.093 (8)	0.029 (4)	0.027 (4)	0.006 (5)	−0.011 (4)	0.013 (3)
C166	0.049 (4)	0.021 (3)	0.022 (3)	0.012 (3)	−0.001 (3)	0.002 (2)
Si8	0.0296 (3)	0.0231 (3)	0.0277 (3)	−0.0002 (2)	−0.0036 (2)	0.0095 (2)
C141	0.0297 (12)	0.0297 (12)	0.0288 (12)	0.0027 (9)	−0.0011 (9)	0.0115 (9)
C142	0.0565 (17)	0.0306 (13)	0.0348 (13)	−0.0027 (12)	−0.0098 (12)	0.0141 (11)
C143	0.083 (2)	0.0369 (15)	0.0387 (15)	0.0027 (15)	−0.0108 (15)	0.0195 (13)
C144	0.0528 (17)	0.0505 (17)	0.0319 (13)	0.0145 (13)	−0.0069 (12)	0.0148 (12)
C145	0.0363 (14)	0.0474 (16)	0.0361 (14)	−0.0004 (12)	−0.0077 (11)	0.0135 (12)
C146	0.0368 (13)	0.0368 (14)	0.0395 (14)	−0.0047 (11)	−0.0061 (11)	0.0183 (11)
C147	0.0388 (13)	0.0320 (13)	0.0318 (12)	−0.0068 (10)	−0.0056 (10)	0.0133 (10)
C148	0.066 (2)	0.0494 (18)	0.0488 (18)	−0.0260 (16)	−0.0022 (15)	0.0211 (15)
C149	0.0450 (16)	0.0549 (18)	0.0457 (16)	0.0009 (13)	0.0123 (13)	0.0224 (14)
C150	0.0532 (18)	0.061 (2)	0.0429 (16)	−0.0001 (15)	−0.0053 (13)	0.0320 (15)
C151	0.0291 (11)	0.0260 (11)	0.0286 (11)	0.0004 (9)	−0.0021 (9)	0.0122 (9)
C152	0.0305 (13)	0.0308 (13)	0.0513 (16)	0.0032 (10)	0.0018 (11)	0.0131 (12)
C153	0.0289 (13)	0.0380 (15)	0.0669 (19)	−0.0050 (11)	−0.0035 (12)	0.0203 (14)
C154	0.0446 (15)	0.0314 (13)	0.0426 (15)	−0.0121 (11)	−0.0096 (11)	0.0165 (11)
C155	0.0486 (15)	0.0223 (11)	0.0310 (12)	0.0040 (10)	−0.0016 (10)	0.0103 (10)
C156	0.0309 (12)	0.0292 (12)	0.0297 (11)	0.0026 (9)	−0.0016 (9)	0.0114 (10)
O22	0.0353 (18)	0.0238 (12)	0.0291 (13)	0.0029 (12)	−0.0001 (10)	0.0088 (8)
C157	0.0247 (19)	0.0318 (15)	0.0384 (16)	0.0017 (13)	0.0043 (15)	0.0112 (12)
O23	0.0285 (10)	0.0377 (11)	0.0303 (10)	0.0021 (8)	−0.0017 (8)	0.0106 (9)
C158	0.0237 (13)	0.0323 (14)	0.0275 (13)	0.0055 (10)	0.0043 (10)	0.0086 (11)
C159	0.0303 (15)	0.0294 (14)	0.0322 (15)	0.0072 (11)	−0.0006 (12)	0.0064 (12)
O24	0.0302 (14)	0.0282 (11)	0.041 (2)	0.0065 (9)	−0.0052 (14)	−0.0001 (12)
O32	0.0353 (18)	0.0238 (12)	0.0291 (13)	0.0029 (12)	−0.0001 (10)	0.0088 (8)
C257	0.033 (7)	0.032 (6)	0.032 (6)	0.007 (6)	0.005 (6)	0.002 (6)
O33	0.055 (9)	0.065 (10)	0.037 (8)	0.033 (8)	0.012 (7)	0.006 (7)
C258	0.038 (6)	0.039 (6)	0.039 (6)	0.015 (5)	0.008 (5)	0.003 (5)
C259	0.033 (7)	0.044 (7)	0.042 (7)	0.013 (6)	−0.005 (6)	−0.002 (7)
O34	0.0302 (14)	0.0282 (11)	0.041 (2)	0.0065 (9)	−0.0052 (14)	−0.0001 (12)

### 3-[(tert-Butyldiphenylsilyl)oxy]propane-1,2-diol (1) Geometric parameters (Å, º)

**Table:**

Si1—O1	1.6513 (17)	C86—H86	0.9500
Si1—C1	1.869 (2)	C87—C88	1.530 (5)
Si1—C7	1.886 (2)	C87—C89	1.526 (4)
Si1—C11	1.881 (2)	C87—C90	1.533 (4)
O1—C17	1.428 (3)	C88—H88A	0.9800
O2—H2	0.8400	C88—H88B	0.9800
O2—C18	1.429 (3)	C88—H88C	0.9800
O3—H3A	0.8400	C89—H89A	0.9800
O3—H3B	0.8400	C89—H89B	0.9800
O3—C19	1.421 (3)	C89—H89C	0.9800
C1—C2	1.396 (4)	C90—H90A	0.9800
C1—C6	1.404 (3)	C90—H90B	0.9800
C2—H2A	0.9500	C90—H90C	0.9800
C2—C3	1.391 (4)	C91—C92	1.398 (4)
C3—H3	0.9500	C91—C96	1.402 (3)
C3—C4	1.392 (5)	C92—H92	0.9500
C4—H4	0.9500	C92—C93	1.383 (4)
C4—C5	1.370 (5)	C93—H93	0.9500
C5—H5	0.9500	C93—C94	1.386 (4)
C5—C6	1.393 (4)	C94—H94	0.9500
C6—H6	0.9500	C94—C95	1.375 (5)
C7—C8	1.535 (3)	C95—H95	0.9500
C7—C9	1.540 (3)	C95—C96	1.380 (4)
C7—C10	1.539 (3)	C96—H96	0.9500
C8—H8A	0.9800	C97—H97A	0.9900
C8—H8B	0.9800	C97—H97B	0.9900
C8—H8C	0.9800	C97—C98	1.509 (3)
C9—H9A	0.9800	C98—H98	1.0000
C9—H9B	0.9800	C98—C99	1.516 (4)
C9—H9C	0.9800	C99—H99A	0.9900
C10—H10G	0.9800	C99—H99B	0.9900
C10—H10H	0.9800	Si6—C101	1.866 (3)
C10—H10I	0.9800	Si6—C107	1.884 (3)
C11—C12	1.399 (3)	Si6—C111	1.880 (3)
C11—C16	1.396 (3)	Si6—O16	1.667 (3)
C12—H12	0.9500	Si6—O216	1.578 (15)
C12—C13	1.389 (4)	C101—C102	1.408 (4)
C13—H13	0.9500	C101—C106	1.409 (4)
C13—C14	1.379 (4)	C102—H102	0.9500
C14—H14	0.9500	C102—C103	1.373 (5)
C14—C15	1.380 (4)	C103—H103	0.9500
C15—H15	0.9500	C103—C104	1.393 (5)
C15—C16	1.391 (3)	C104—H104	0.9500
C16—H16	0.9500	C104—C105	1.373 (5)
C17—H17A	0.9900	C105—H105	0.9500
C17—H17B	0.9900	C105—C106	1.386 (4)
C17—C18	1.513 (3)	C106—H106	0.9500
C18—H18A	1.0000	C107—C108	1.537 (4)
C18—C19	1.518 (4)	C107—C109	1.543 (4)
C19—H19A	0.9900	C107—C110	1.535 (4)
C19—H19B	0.9900	C108—H10A	0.9800
Si2—O4	1.6392 (19)	C108—H10B	0.9800
Si2—C21	1.873 (3)	C108—H10C	0.9800
Si2—C27	1.892 (3)	C109—H10D	0.9800
Si2—C31	1.876 (3)	C109—H10E	0.9800
O4—C37	1.412 (3)	C109—H10F	0.9800
O5—H5A	0.8400	C110—H11A	0.9800
O5—C38	1.432 (3)	C110—H11B	0.9800
O6—H6A	0.8400	C110—H11C	0.9800
O6—C39	1.437 (3)	C111—C112	1.405 (4)
C21—C22	1.412 (4)	C111—C116	1.398 (4)
C21—C26	1.389 (4)	C112—H112	0.9500
C22—H22	0.9500	C112—C113	1.392 (4)
C22—C23	1.386 (4)	C113—H113	0.9500
C23—H23A	0.9500	C113—C114	1.385 (4)
C23—C24	1.366 (5)	C114—H114	0.9500
C24—H24A	0.9500	C114—C115	1.384 (4)
C24—C25	1.394 (4)	C115—H115	0.9500
C25—H25	0.9500	C115—C116	1.384 (4)
C25—C26	1.397 (4)	C116—H116	0.9500
C26—H26	0.9500	O16—C117	1.419 (5)
C27—C28	1.546 (4)	O17—H17	0.8400
C27—C29	1.527 (4)	O17—C118	1.439 (4)
C27—C30	1.541 (4)	O18—H18	0.8400
C28—H28A	0.9800	O18—C119	1.438 (4)
C28—H28B	0.9800	C117—H11D	0.9900
C28—H28C	0.9800	C117—H11E	0.9900
C29—H29A	0.9800	C117—C118	1.503 (4)
C29—H29B	0.9800	C118—H118	1.0000
C29—H29C	0.9800	C118—C119	1.521 (5)
C30—H30A	0.9800	C119—H11F	0.9900
C30—H30B	0.9800	C119—H11G	0.9900
C30—H30C	0.9800	O216—C217	1.400 (19)
C31—C32	1.394 (4)	O217—H217	0.8400
C31—C36	1.397 (3)	O217—C218	1.391 (16)
C32—H32	0.9500	O218—H218	0.8400
C32—C33	1.383 (4)	O218—C219	1.467 (14)
C33—H33A	0.9500	C217—H21A	0.9900
C33—C34	1.382 (4)	C217—H21B	0.9900
C34—H34A	0.9500	C217—C218	1.498 (19)
C34—C35	1.381 (4)	C218—H21C	1.0000
C35—H35	0.9500	C218—C219	1.51 (2)
C35—C36	1.388 (4)	C219—H21D	0.9900
C36—H36	0.9500	C219—H21E	0.9900
C37—H37A	0.9900	Si7—O19	1.6418 (19)
C37—H37B	0.9900	Si7—C127	1.886 (3)
C37—C38	1.511 (4)	Si7—C131	1.867 (3)
C38—H38	1.0000	Si7—C121	1.853 (11)
C38—C39	1.503 (4)	Si7—C161	1.93 (2)
C39—H39A	0.9900	O19—C137	1.407 (3)
C39—H39B	0.9900	O20—H20	0.8400
Si3—O7	1.6488 (18)	O20—C138	1.429 (3)
Si3—C41	1.882 (3)	O21—H21	0.8400
Si3—C47	1.887 (3)	O21—C139	1.420 (3)
Si3—C51	1.869 (2)	C127—C128	1.530 (4)
O7—C57	1.421 (3)	C127—C129	1.544 (4)
O8—H8	0.8400	C127—C130	1.539 (4)
O8—C58	1.444 (3)	C128—H12A	0.9800
O9—H9	0.8400	C128—H12B	0.9800
O9—C59	1.429 (4)	C128—H12C	0.9800
C41—C42	1.396 (4)	C129—H12D	0.9800
C41—C46	1.398 (4)	C129—H12E	0.9800
C42—H42	0.9500	C129—H12F	0.9800
C42—C43	1.397 (5)	C130—H13A	0.9800
C43—H43	0.9500	C130—H13B	0.9800
C43—C44	1.376 (5)	C130—H13C	0.9800
C44—H44	0.9500	C131—C132	1.393 (4)
C44—C45	1.373 (5)	C131—C136	1.391 (4)
C45—H45	0.9500	C132—H132	0.9500
C45—C46	1.394 (4)	C132—C133	1.383 (4)
C46—H46	0.9500	C133—H133	0.9500
C47—C48	1.540 (4)	C133—C134	1.378 (5)
C47—C49	1.535 (5)	C134—H134	0.9500
C47—C50	1.539 (4)	C134—C135	1.376 (5)
C48—H48A	0.9800	C135—H135	0.9500
C48—H48B	0.9800	C135—C136	1.387 (4)
C48—H48C	0.9800	C136—H136	0.9500
C49—H49A	0.9800	C137—H13D	0.9900
C49—H49B	0.9800	C137—H13E	0.9900
C49—H49C	0.9800	C137—C138	1.514 (4)
C50—H50A	0.9800	C138—H138	1.0000
C50—H50B	0.9800	C138—C139	1.501 (4)
C50—H50C	0.9800	C139—H13F	0.9900
C51—C52	1.400 (3)	C139—H13G	0.9900
C51—C56	1.395 (3)	C121—C122	1.376 (13)
C52—H52	0.9500	C121—C126	1.387 (13)
C52—C53	1.390 (3)	C122—H122	0.9500
C53—H53	0.9500	C122—C123	1.397 (7)
C53—C54	1.379 (4)	C123—H123	0.9500
C54—H54	0.9500	C123—C124	1.335 (12)
C54—C55	1.380 (4)	C124—H124	0.9500
C55—H55	0.9500	C124—C125	1.374 (12)
C55—C56	1.391 (4)	C125—H125	0.9500
C56—H56	0.9500	C125—C126	1.408 (7)
C57—H57A	0.9900	C126—H126	0.9500
C57—H57B	0.9900	C161—C162	1.394 (18)
C57—C58	1.502 (4)	C161—C166	1.389 (17)
C58—H58	1.0000	C162—H162	0.9275
C58—C59	1.504 (4)	C162—C163	1.390 (9)
C59—H59A	0.9900	C163—H163	0.9228
C59—H59B	0.9900	C163—C164	1.361 (17)
Si4—O10	1.6341 (18)	C164—H164	0.9500
Si4—C61	1.868 (3)	C164—C165	1.356 (16)
Si4—C67	1.884 (3)	C165—H165	0.9275
Si4—C71	1.873 (2)	C165—C166	1.401 (10)
O10—C77	1.407 (3)	C166—H166	0.9235
O11—H11	0.8400	Si8—C141	1.881 (2)
O11—C78	1.422 (3)	Si8—C147	1.897 (3)
O12—H12G	0.8400	Si8—C151	1.879 (2)
O12—C79	1.433 (4)	Si8—O22	1.638 (3)
C61—C62	1.405 (4)	Si8—O32	1.73 (2)
C61—C66	1.388 (4)	C141—C142	1.390 (4)
C62—H62	0.9500	C141—C146	1.395 (4)
C62—C63	1.380 (4)	C142—H142	0.9500
C63—H63	0.9500	C142—C143	1.387 (4)
C63—C64	1.375 (6)	C143—H143	0.9500
C64—H64	0.9500	C143—C144	1.376 (5)
C64—C65	1.366 (6)	C144—H144	0.9500
C65—H65	0.9500	C144—C145	1.367 (4)
C65—C66	1.404 (5)	C145—H145	0.9500
C66—H66	0.9500	C145—C146	1.389 (4)
C67—C68	1.535 (4)	C146—H146	0.9500
C67—C69	1.529 (4)	C147—C148	1.534 (4)
C67—C70	1.542 (3)	C147—C149	1.528 (4)
C68—H68A	0.9800	C147—C150	1.533 (4)
C68—H68B	0.9800	C148—H14A	0.9800
C68—H68C	0.9800	C148—H14B	0.9800
C69—H69A	0.9800	C148—H14C	0.9800
C69—H69B	0.9800	C149—H14D	0.9800
C69—H69C	0.9800	C149—H14E	0.9800
C70—H70A	0.9800	C149—H14F	0.9800
C70—H70B	0.9800	C150—H15A	0.9800
C70—H70C	0.9800	C150—H15B	0.9800
C71—C72	1.399 (4)	C150—H15C	0.9800
C71—C76	1.382 (4)	C151—C152	1.392 (4)
C72—H72	0.9500	C151—C156	1.404 (3)
C72—C73	1.384 (4)	C152—H152	0.9500
C73—H73	0.9500	C152—C153	1.397 (4)
C73—C74	1.381 (5)	C153—H153	0.9500
C74—H74	0.9500	C153—C154	1.367 (4)
C74—C75	1.368 (5)	C154—H154	0.9500
C75—H75	0.9500	C154—C155	1.381 (4)
C75—C76	1.379 (5)	C155—H155	0.9500
C76—H76	0.9500	C155—C156	1.390 (4)
C77—H77A	0.9900	C156—H156	0.9500
C77—H77B	0.9900	O22—C157	1.420 (5)
C77—C78	1.514 (4)	C157—H15D	0.9900
C78—H78	1.0000	C157—H15E	0.9900
C78—C79	1.507 (4)	C157—C158	1.507 (5)
C79—H79A	0.9900	O23—H23	0.8400
C79—H79B	0.9900	O23—C158	1.432 (3)
Si5—O13	1.6474 (17)	C158—H158	1.0000
Si5—C81	1.874 (3)	C158—C159	1.517 (4)
Si5—C87	1.895 (3)	C159—H15F	0.9900
Si5—C91	1.876 (3)	C159—H15G	0.9900
O13—C97	1.434 (3)	C159—O24	1.449 (4)
O14—H14G	0.8400	O24—H24	0.8400
O14—C98	1.441 (3)	O32—C257	1.44 (2)
O15—H15H	0.8400	C257—H25A	0.9900
O15—C99	1.427 (3)	C257—H25B	0.9900
C81—C82	1.398 (4)	C257—C258	1.52 (2)
C81—C86	1.398 (4)	O33—H33	0.8400
C82—H82	0.9500	O33—C258	1.43 (2)
C82—C83	1.392 (4)	C258—H258	1.0000
C83—H83	0.9500	C258—C259	1.47 (2)
C83—C84	1.374 (5)	C259—H25C	0.9900
C84—H84	0.9500	C259—H25D	0.9900
C84—C85	1.383 (4)	C259—O34	1.45 (2)
C85—H85	0.9500	O34—H34	1.01 (4)
C85—C86	1.385 (4)		
			
O1—Si1—C1	107.86 (10)	C87—C88—H88B	109.5
O1—Si1—C7	104.13 (10)	C87—C88—H88C	109.5
O1—Si1—C11	109.02 (9)	H88A—C88—H88B	109.5
C1—Si1—C7	110.88 (11)	H88A—C88—H88C	109.5
C1—Si1—C11	109.11 (10)	H88B—C88—H88C	109.5
C11—Si1—C7	115.49 (11)	C87—C89—H89A	109.5
C17—O1—Si1	122.24 (15)	C87—C89—H89B	109.5
C18—O2—H2	109.5	C87—C89—H89C	109.5
C19—O3—H3A	109.5	H89A—C89—H89B	109.5
C19—O3—H3B	109.5	H89A—C89—H89C	109.5
C2—C1—Si1	122.78 (19)	H89B—C89—H89C	109.5
C2—C1—C6	117.2 (2)	C87—C90—H90A	109.5
C6—C1—Si1	119.96 (19)	C87—C90—H90B	109.5
C1—C2—H2A	119.2	C87—C90—H90C	109.5
C3—C2—C1	121.6 (3)	H90A—C90—H90B	109.5
C3—C2—H2A	119.2	H90A—C90—H90C	109.5
C2—C3—H3	120.3	H90B—C90—H90C	109.5
C2—C3—C4	119.5 (3)	C92—C91—Si5	119.14 (19)
C4—C3—H3	120.3	C92—C91—C96	116.7 (2)
C3—C4—H4	119.8	C96—C91—Si5	124.2 (2)
C5—C4—C3	120.4 (3)	C91—C92—H92	119.0
C5—C4—H4	119.8	C93—C92—C91	122.0 (3)
C4—C5—H5	120.0	C93—C92—H92	119.0
C4—C5—C6	119.9 (3)	C92—C93—H93	120.1
C6—C5—H5	120.0	C92—C93—C94	119.8 (3)
C1—C6—H6	119.3	C94—C93—H93	120.1
C5—C6—C1	121.4 (3)	C93—C94—H94	120.3
C5—C6—H6	119.3	C95—C94—C93	119.4 (3)
C8—C7—Si1	111.52 (16)	C95—C94—H94	120.3
C8—C7—C9	108.2 (2)	C94—C95—H95	119.7
C8—C7—C10	110.0 (2)	C94—C95—C96	120.7 (3)
C9—C7—Si1	107.47 (16)	C96—C95—H95	119.7
C10—C7—Si1	111.49 (16)	C91—C96—H96	119.3
C10—C7—C9	108.0 (2)	C95—C96—C91	121.4 (3)
C7—C8—H8A	109.5	C95—C96—H96	119.3
C7—C8—H8B	109.5	O13—C97—H97A	110.0
C7—C8—H8C	109.5	O13—C97—H97B	110.0
H8A—C8—H8B	109.5	O13—C97—C98	108.3 (2)
H8A—C8—H8C	109.5	H97A—C97—H97B	108.4
H8B—C8—H8C	109.5	C98—C97—H97A	110.0
C7—C9—H9A	109.5	C98—C97—H97B	110.0
C7—C9—H9B	109.5	O14—C98—C97	110.3 (2)
C7—C9—H9C	109.5	O14—C98—H98	107.9
H9A—C9—H9B	109.5	O14—C98—C99	108.7 (2)
H9A—C9—H9C	109.5	C97—C98—H98	107.9
H9B—C9—H9C	109.5	C97—C98—C99	113.9 (2)
C7—C10—H10G	109.5	C99—C98—H98	107.9
C7—C10—H10H	109.5	O15—C99—C98	111.2 (2)
C7—C10—H10I	109.5	O15—C99—H99A	109.4
H10G—C10—H10H	109.5	O15—C99—H99B	109.4
H10G—C10—H10I	109.5	C98—C99—H99A	109.4
H10H—C10—H10I	109.5	C98—C99—H99B	109.4
C12—C11—Si1	118.92 (18)	H99A—C99—H99B	108.0
C16—C11—Si1	124.13 (18)	C101—Si6—C107	110.08 (12)
C16—C11—C12	116.9 (2)	C101—Si6—C111	106.80 (12)
C11—C12—H12	119.0	C111—Si6—C107	115.81 (12)
C13—C12—C11	122.1 (2)	O16—Si6—C101	112.65 (15)
C13—C12—H12	119.0	O16—Si6—C107	100.80 (15)
C12—C13—H13	120.3	O16—Si6—C111	110.80 (16)
C14—C13—C12	119.5 (2)	O216—Si6—C101	97.3 (5)
C14—C13—H13	120.3	O216—Si6—C107	115.8 (5)
C13—C14—H14	120.0	O216—Si6—C111	109.1 (7)
C13—C14—C15	120.0 (2)	C102—C101—Si6	122.0 (2)
C15—C14—H14	120.0	C102—C101—C106	116.7 (3)
C14—C15—H15	119.9	C106—C101—Si6	121.3 (2)
C14—C15—C16	120.1 (2)	C101—C102—H102	119.2
C16—C15—H15	119.9	C103—C102—C101	121.7 (3)
C11—C16—H16	119.3	C103—C102—H102	119.2
C15—C16—C11	121.4 (2)	C102—C103—H103	119.9
C15—C16—H16	119.3	C102—C103—C104	120.2 (3)
O1—C17—H17A	109.8	C104—C103—H103	119.9
O1—C17—H17B	109.8	C103—C104—H104	120.1
O1—C17—C18	109.33 (19)	C105—C104—C103	119.8 (3)
H17A—C17—H17B	108.3	C105—C104—H104	120.1
C18—C17—H17A	109.8	C104—C105—H105	119.9
C18—C17—H17B	109.8	C104—C105—C106	120.2 (3)
O2—C18—C17	108.8 (2)	C106—C105—H105	119.9
O2—C18—H18A	108.4	C101—C106—H106	119.3
O2—C18—C19	111.1 (2)	C105—C106—C101	121.5 (3)
C17—C18—H18A	108.4	C105—C106—H106	119.3
C17—C18—C19	111.6 (2)	C108—C107—Si6	108.1 (2)
C19—C18—H18A	108.4	C108—C107—C109	108.1 (2)
O3—C19—C18	112.2 (2)	C109—C107—Si6	110.72 (19)
O3—C19—H19A	109.2	C110—C107—Si6	111.22 (19)
O3—C19—H19B	109.2	C110—C107—C108	108.4 (2)
C18—C19—H19A	109.2	C110—C107—C109	110.2 (2)
C18—C19—H19B	109.2	C107—C108—H10A	109.5
H19A—C19—H19B	107.9	C107—C108—H10B	109.5
O4—Si2—C21	111.21 (11)	C107—C108—H10C	109.5
O4—Si2—C27	106.04 (13)	H10A—C108—H10B	109.5
O4—Si2—C31	104.90 (11)	H10A—C108—H10C	109.5
C21—Si2—C27	115.08 (12)	H10B—C108—H10C	109.5
C21—Si2—C31	109.24 (11)	C107—C109—H10D	109.5
C31—Si2—C27	109.86 (12)	C107—C109—H10E	109.5
C37—O4—Si2	131.25 (18)	C107—C109—H10F	109.5
C38—O5—H5A	109.5	H10D—C109—H10E	109.5
C39—O6—H6A	109.5	H10D—C109—H10F	109.5
C22—C21—Si2	124.1 (2)	H10E—C109—H10F	109.5
C26—C21—Si2	118.71 (19)	C107—C110—H11A	109.5
C26—C21—C22	117.1 (2)	C107—C110—H11B	109.5
C21—C22—H22	119.2	C107—C110—H11C	109.5
C23—C22—C21	121.6 (3)	H11A—C110—H11B	109.5
C23—C22—H22	119.2	H11A—C110—H11C	109.5
C22—C23—H23A	120.0	H11B—C110—H11C	109.5
C24—C23—C22	120.0 (3)	C112—C111—Si6	118.69 (19)
C24—C23—H23A	120.0	C116—C111—Si6	124.7 (2)
C23—C24—H24A	119.8	C116—C111—C112	116.6 (2)
C23—C24—C25	120.4 (3)	C111—C112—H112	119.0
C25—C24—H24A	119.8	C113—C112—C111	121.9 (3)
C24—C25—H25	120.3	C113—C112—H112	119.0
C24—C25—C26	119.5 (3)	C112—C113—H113	120.2
C26—C25—H25	120.3	C114—C113—C112	119.7 (3)
C21—C26—C25	121.4 (3)	C114—C113—H113	120.2
C21—C26—H26	119.3	C113—C114—H114	120.2
C25—C26—H26	119.3	C115—C114—C113	119.6 (2)
C28—C27—Si2	111.3 (2)	C115—C114—H114	120.2
C29—C27—Si2	111.3 (2)	C114—C115—H115	119.8
C29—C27—C28	110.1 (3)	C116—C115—C114	120.4 (2)
C29—C27—C30	108.8 (3)	C116—C115—H115	119.8
C30—C27—Si2	107.8 (2)	C111—C116—H116	119.1
C30—C27—C28	107.4 (2)	C115—C116—C111	121.8 (2)
C27—C28—H28A	109.5	C115—C116—H116	119.1
C27—C28—H28B	109.5	C117—O16—Si6	129.2 (3)
C27—C28—H28C	109.5	C118—O17—H17	109.5
H28A—C28—H28B	109.5	C119—O18—H18	109.5
H28A—C28—H28C	109.5	O16—C117—H11D	109.6
H28B—C28—H28C	109.5	O16—C117—H11E	109.6
C27—C29—H29A	109.5	O16—C117—C118	110.4 (3)
C27—C29—H29B	109.5	H11D—C117—H11E	108.1
C27—C29—H29C	109.5	C118—C117—H11D	109.6
H29A—C29—H29B	109.5	C118—C117—H11E	109.6
H29A—C29—H29C	109.5	O17—C118—C117	109.3 (2)
H29B—C29—H29C	109.5	O17—C118—H118	107.8
C27—C30—H30A	109.5	O17—C118—C119	110.0 (3)
C27—C30—H30B	109.5	C117—C118—H118	107.8
C27—C30—H30C	109.5	C117—C118—C119	113.9 (3)
H30A—C30—H30B	109.5	C119—C118—H118	107.8
H30A—C30—H30C	109.5	O18—C119—C118	109.3 (3)
H30B—C30—H30C	109.5	O18—C119—H11F	109.8
C32—C31—Si2	123.29 (18)	O18—C119—H11G	109.8
C32—C31—C36	117.0 (2)	C118—C119—H11F	109.8
C36—C31—Si2	119.66 (19)	C118—C119—H11G	109.8
C31—C32—H32	119.3	H11F—C119—H11G	108.3
C33—C32—C31	121.4 (2)	C217—O216—Si6	131.8 (12)
C33—C32—H32	119.3	C218—O217—H217	109.5
C32—C33—H33A	119.7	C219—O218—H218	109.5
C34—C33—C32	120.6 (3)	O216—C217—H21A	110.1
C34—C33—H33A	119.7	O216—C217—H21B	110.1
C33—C34—H34A	120.4	O216—C217—C218	108.2 (12)
C35—C34—C33	119.2 (2)	H21A—C217—H21B	108.4
C35—C34—H34A	120.4	C218—C217—H21A	110.1
C34—C35—H35	120.0	C218—C217—H21B	110.1
C34—C35—C36	120.0 (3)	O217—C218—C217	114.3 (14)
C36—C35—H35	120.0	O217—C218—H21C	107.6
C31—C36—H36	119.2	O217—C218—C219	111.0 (11)
C35—C36—C31	121.7 (3)	C217—C218—H21C	107.6
C35—C36—H36	119.2	C217—C218—C219	108.5 (11)
O4—C37—H37A	109.7	C219—C218—H21C	107.6
O4—C37—H37B	109.7	O218—C219—C218	113.6 (11)
O4—C37—C38	109.9 (2)	O218—C219—H21D	108.9
H37A—C37—H37B	108.2	O218—C219—H21E	108.9
C38—C37—H37A	109.7	C218—C219—H21D	108.9
C38—C37—H37B	109.7	C218—C219—H21E	108.9
O5—C38—C37	106.9 (2)	H21D—C219—H21E	107.7
O5—C38—H38	109.0	O19—Si7—C127	112.72 (12)
O5—C38—C39	111.5 (2)	O19—Si7—C131	103.08 (10)
C37—C38—H38	109.0	O19—Si7—C121	107.4 (3)
C39—C38—C37	111.3 (3)	O19—Si7—C161	111.5 (5)
C39—C38—H38	109.0	C127—Si7—C161	108.8 (8)
O6—C39—C38	109.7 (2)	C131—Si7—C127	109.51 (12)
O6—C39—H39A	109.7	C131—Si7—C161	111.2 (11)
O6—C39—H39B	109.7	C121—Si7—C127	115.4 (4)
C38—C39—H39A	109.7	C121—Si7—C131	108.0 (6)
C38—C39—H39B	109.7	C137—O19—Si7	132.15 (17)
H39A—C39—H39B	108.2	C138—O20—H20	109.5
O7—Si3—C41	110.06 (10)	C139—O21—H21	109.5
O7—Si3—C47	110.64 (12)	C128—C127—Si7	111.0 (2)
O7—Si3—C51	103.04 (10)	C128—C127—C129	108.2 (3)
C41—Si3—C47	114.59 (12)	C128—C127—C130	108.7 (2)
C51—Si3—C41	108.23 (11)	C129—C127—Si7	107.4 (2)
C51—Si3—C47	109.62 (11)	C130—C127—Si7	112.86 (19)
C57—O7—Si3	123.53 (18)	C130—C127—C129	108.5 (2)
C58—O8—H8	109.5	C127—C128—H12A	109.5
C59—O9—H9	109.5	C127—C128—H12B	109.5
C42—C41—Si3	125.7 (2)	C127—C128—H12C	109.5
C42—C41—C46	116.9 (2)	H12A—C128—H12B	109.5
C46—C41—Si3	117.33 (18)	H12A—C128—H12C	109.5
C41—C42—H42	119.5	H12B—C128—H12C	109.5
C41—C42—C43	121.0 (3)	C127—C129—H12D	109.5
C43—C42—H42	119.5	C127—C129—H12E	109.5
C42—C43—H43	119.7	C127—C129—H12F	109.5
C44—C43—C42	120.6 (3)	H12D—C129—H12E	109.5
C44—C43—H43	119.7	H12D—C129—H12F	109.5
C43—C44—H44	120.2	H12E—C129—H12F	109.5
C45—C44—C43	119.7 (3)	C127—C130—H13A	109.5
C45—C44—H44	120.2	C127—C130—H13B	109.5
C44—C45—H45	120.1	C127—C130—H13C	109.5
C44—C45—C46	119.9 (3)	H13A—C130—H13B	109.5
C46—C45—H45	120.1	H13A—C130—H13C	109.5
C41—C46—H46	119.1	H13B—C130—H13C	109.5
C45—C46—C41	121.9 (3)	C132—C131—Si7	121.33 (19)
C45—C46—H46	119.1	C136—C131—Si7	121.6 (2)
C48—C47—Si3	107.4 (2)	C136—C131—C132	117.0 (2)
C48—C47—C50	107.7 (3)	C131—C132—H132	119.1
C49—C47—Si3	109.6 (2)	C133—C132—C131	121.9 (3)
C49—C47—C48	109.7 (3)	C133—C132—H132	119.1
C49—C47—C50	108.8 (3)	C132—C133—H133	120.0
C50—C47—Si3	113.6 (2)	C134—C133—C132	120.0 (3)
C47—C48—H48A	109.5	C134—C133—H133	120.0
C47—C48—H48B	109.5	C133—C134—H134	120.3
C47—C48—H48C	109.5	C135—C134—C133	119.4 (3)
H48A—C48—H48B	109.5	C135—C134—H134	120.3
H48A—C48—H48C	109.5	C134—C135—H135	119.7
H48B—C48—H48C	109.5	C134—C135—C136	120.5 (3)
C47—C49—H49A	109.5	C136—C135—H135	119.7
C47—C49—H49B	109.5	C131—C136—H136	119.4
C47—C49—H49C	109.5	C135—C136—C131	121.2 (3)
H49A—C49—H49B	109.5	C135—C136—H136	119.4
H49A—C49—H49C	109.5	O19—C137—H13D	109.5
H49B—C49—H49C	109.5	O19—C137—H13E	109.5
C47—C50—H50A	109.5	O19—C137—C138	110.5 (2)
C47—C50—H50B	109.5	H13D—C137—H13E	108.1
C47—C50—H50C	109.5	C138—C137—H13D	109.5
H50A—C50—H50B	109.5	C138—C137—H13E	109.5
H50A—C50—H50C	109.5	O20—C138—C137	113.3 (2)
H50B—C50—H50C	109.5	O20—C138—H138	106.6
C52—C51—Si3	121.94 (18)	O20—C138—C139	112.9 (2)
C56—C51—Si3	120.62 (18)	C137—C138—H138	106.6
C56—C51—C52	117.4 (2)	C139—C138—C137	110.4 (2)
C51—C52—H52	119.4	C139—C138—H138	106.6
C53—C52—C51	121.2 (2)	O21—C139—C138	113.1 (2)
C53—C52—H52	119.4	O21—C139—H13F	109.0
C52—C53—H53	120.1	O21—C139—H13G	109.0
C54—C53—C52	119.9 (2)	C138—C139—H13F	109.0
C54—C53—H53	120.1	C138—C139—H13G	109.0
C53—C54—H54	119.9	H13F—C139—H13G	107.8
C53—C54—C55	120.2 (2)	C122—C121—Si7	125.0 (9)
C55—C54—H54	119.9	C122—C121—C126	117.0 (8)
C54—C55—H55	120.1	C126—C121—Si7	118.0 (8)
C54—C55—C56	119.7 (2)	C121—C122—H122	119.0
C56—C55—H55	120.1	C121—C122—C123	122.1 (7)
C51—C56—H56	119.3	C123—C122—H122	119.0
C55—C56—C51	121.4 (2)	C122—C123—H123	119.6
C55—C56—H56	119.3	C124—C123—C122	120.8 (7)
O7—C57—H57A	109.5	C124—C123—H123	119.6
O7—C57—H57B	109.5	C123—C124—H124	120.6
O7—C57—C58	110.7 (2)	C123—C124—C125	118.9 (8)
H57A—C57—H57B	108.1	C125—C124—H124	120.6
C58—C57—H57A	109.5	C124—C125—H125	119.4
C58—C57—H57B	109.5	C124—C125—C126	121.2 (6)
O8—C58—C57	108.8 (2)	C126—C125—H125	119.4
O8—C58—H58	108.4	C121—C126—C125	119.9 (6)
O8—C58—C59	111.2 (2)	C121—C126—H126	120.0
C57—C58—H58	108.4	C125—C126—H126	120.0
C57—C58—C59	111.5 (2)	C162—C161—Si7	122.8 (13)
C59—C58—H58	108.4	C166—C161—Si7	118.9 (11)
O9—C59—C58	110.4 (2)	C166—C161—C162	117.6 (15)
O9—C59—H59A	109.6	C161—C162—H162	119.4
O9—C59—H59B	109.6	C163—C162—C161	120.1 (10)
C58—C59—H59A	109.6	C163—C162—H162	120.4
C58—C59—H59B	109.6	C162—C163—H163	120.3
H59A—C59—H59B	108.1	C164—C163—C162	121.6 (10)
O10—Si4—C61	101.57 (11)	C164—C163—H163	118.0
O10—Si4—C67	110.92 (11)	C163—C164—H164	120.4
O10—Si4—C71	109.34 (11)	C165—C164—C163	119.1 (15)
C61—Si4—C67	111.91 (12)	C165—C164—H164	120.4
C61—Si4—C71	107.76 (11)	C164—C165—H165	119.9
C71—Si4—C67	114.51 (11)	C164—C165—C166	120.8 (10)
C77—O10—Si4	135.04 (18)	C166—C165—H165	119.3
C78—O11—H11	109.5	C161—C166—C165	120.6 (10)
C79—O12—H12G	109.5	C161—C166—H166	120.9
C62—C61—Si4	121.6 (2)	C165—C166—H166	118.4
C66—C61—Si4	121.0 (2)	C141—Si8—C147	115.86 (11)
C66—C61—C62	117.2 (3)	C151—Si8—C141	110.26 (11)
C61—C62—H62	119.2	C151—Si8—C147	108.95 (11)
C63—C62—C61	121.7 (3)	O22—Si8—C141	109.4 (2)
C63—C62—H62	119.2	O22—Si8—C147	102.70 (14)
C62—C63—H63	120.1	O22—Si8—C151	109.29 (17)
C64—C63—C62	119.8 (3)	O32—Si8—C141	107.4 (14)
C64—C63—H63	120.1	O32—Si8—C147	112.0 (11)
C63—C64—H64	119.8	O32—Si8—C151	101.4 (13)
C65—C64—C63	120.4 (3)	C142—C141—Si8	121.35 (19)
C65—C64—H64	119.8	C142—C141—C146	116.6 (2)
C64—C65—H65	120.0	C146—C141—Si8	121.89 (19)
C64—C65—C66	120.0 (3)	C141—C142—H142	119.2
C66—C65—H65	120.0	C143—C142—C141	121.5 (3)
C61—C66—C65	121.0 (3)	C143—C142—H142	119.2
C61—C66—H66	119.5	C142—C143—H143	119.7
C65—C66—H66	119.5	C144—C143—C142	120.5 (3)
C68—C67—Si4	111.97 (18)	C144—C143—H143	119.7
C68—C67—C70	108.0 (2)	C143—C144—H144	120.4
C69—C67—Si4	110.89 (19)	C145—C144—C143	119.2 (3)
C69—C67—C68	109.1 (2)	C145—C144—H144	120.4
C69—C67—C70	110.0 (2)	C144—C145—H145	119.8
C70—C67—Si4	106.87 (17)	C144—C145—C146	120.4 (3)
C67—C68—H68A	109.5	C146—C145—H145	119.8
C67—C68—H68B	109.5	C141—C146—H146	119.2
C67—C68—H68C	109.5	C145—C146—C141	121.6 (3)
H68A—C68—H68B	109.5	C145—C146—H146	119.2
H68A—C68—H68C	109.5	C148—C147—Si8	111.54 (19)
H68B—C68—H68C	109.5	C149—C147—Si8	111.03 (18)
C67—C69—H69A	109.5	C149—C147—C148	108.8 (3)
C67—C69—H69B	109.5	C149—C147—C150	109.0 (2)
C67—C69—H69C	109.5	C150—C147—Si8	107.82 (19)
H69A—C69—H69B	109.5	C150—C147—C148	108.5 (2)
H69A—C69—H69C	109.5	C147—C148—H14A	109.5
H69B—C69—H69C	109.5	C147—C148—H14B	109.5
C67—C70—H70A	109.5	C147—C148—H14C	109.5
C67—C70—H70B	109.5	H14A—C148—H14B	109.5
C67—C70—H70C	109.5	H14A—C148—H14C	109.5
H70A—C70—H70B	109.5	H14B—C148—H14C	109.5
H70A—C70—H70C	109.5	C147—C149—H14D	109.5
H70B—C70—H70C	109.5	C147—C149—H14E	109.5
C72—C71—Si4	123.8 (2)	C147—C149—H14F	109.5
C76—C71—Si4	119.5 (2)	H14D—C149—H14E	109.5
C76—C71—C72	116.3 (2)	H14D—C149—H14F	109.5
C71—C72—H72	118.9	H14E—C149—H14F	109.5
C73—C72—C71	122.2 (3)	C147—C150—H15A	109.5
C73—C72—H72	118.9	C147—C150—H15B	109.5
C72—C73—H73	120.2	C147—C150—H15C	109.5
C74—C73—C72	119.7 (3)	H15A—C150—H15B	109.5
C74—C73—H73	120.2	H15A—C150—H15C	109.5
C73—C74—H74	120.6	H15B—C150—H15C	109.5
C75—C74—C73	118.9 (3)	C152—C151—Si8	119.24 (19)
C75—C74—H74	120.6	C152—C151—C156	116.8 (2)
C74—C75—H75	119.4	C156—C151—Si8	123.78 (18)
C74—C75—C76	121.3 (3)	C151—C152—H152	119.1
C76—C75—H75	119.4	C151—C152—C153	121.7 (2)
C71—C76—H76	119.2	C153—C152—H152	119.1
C75—C76—C71	121.6 (3)	C152—C153—H153	120.0
C75—C76—H76	119.2	C154—C153—C152	120.0 (3)
O10—C77—H77A	110.3	C154—C153—H153	120.0
O10—C77—H77B	110.3	C153—C154—H154	120.1
O10—C77—C78	107.3 (2)	C153—C154—C155	119.9 (2)
H77A—C77—H77B	108.5	C155—C154—H154	120.1
C78—C77—H77A	110.3	C154—C155—H155	119.9
C78—C77—H77B	110.3	C154—C155—C156	120.2 (2)
O11—C78—C77	110.1 (2)	C156—C155—H155	119.9
O11—C78—H78	109.2	C151—C156—H156	119.4
O11—C78—C79	107.1 (2)	C155—C156—C151	121.3 (2)
C77—C78—H78	109.2	C155—C156—H156	119.4
C79—C78—C77	112.0 (2)	C157—O22—Si8	124.8 (3)
C79—C78—H78	109.2	O22—C157—H15D	109.7
O12—C79—C78	109.9 (3)	O22—C157—H15E	109.7
O12—C79—H79A	109.7	O22—C157—C158	109.8 (3)
O12—C79—H79B	109.7	H15D—C157—H15E	108.2
C78—C79—H79A	109.7	C158—C157—H15D	109.7
C78—C79—H79B	109.7	C158—C157—H15E	109.7
H79A—C79—H79B	108.2	C158—O23—H23	109.5
O13—Si5—C81	110.14 (10)	C157—C158—H158	108.7
O13—Si5—C87	104.69 (11)	C157—C158—C159	113.0 (3)
O13—Si5—C91	108.45 (11)	O23—C158—C157	110.1 (2)
C81—Si5—C87	112.28 (12)	O23—C158—H158	108.7
C81—Si5—C91	109.87 (12)	O23—C158—C159	107.4 (2)
C91—Si5—C87	111.23 (12)	C159—C158—H158	108.7
C97—O13—Si5	125.71 (15)	C158—C159—H15F	109.7
C98—O14—H14G	109.5	C158—C159—H15G	109.7
C99—O15—H15H	109.5	H15F—C159—H15G	108.2
C82—C81—Si5	122.1 (2)	O24—C159—C158	110.0 (3)
C86—C81—Si5	120.9 (2)	O24—C159—H15F	109.7
C86—C81—C82	117.0 (2)	O24—C159—H15G	109.7
C81—C82—H82	119.4	C159—O24—H24	109.5
C83—C82—C81	121.2 (3)	C257—O32—Si8	139 (3)
C83—C82—H82	119.4	O32—C257—H25A	108.1
C82—C83—H83	119.9	O32—C257—H25B	108.1
C84—C83—C82	120.3 (3)	O32—C257—C258	117 (2)
C84—C83—H83	119.9	H25A—C257—H25B	107.3
C83—C84—H84	120.1	C258—C257—H25A	108.1
C83—C84—C85	119.9 (3)	C258—C257—H25B	108.1
C85—C84—H84	120.1	C258—O33—H33	109.5
C84—C85—H85	120.1	C257—C258—H258	108.5
C84—C85—C86	119.8 (3)	O33—C258—C257	110.6 (19)
C86—C85—H85	120.1	O33—C258—H258	108.5
C81—C86—H86	119.1	O33—C258—C259	107.2 (18)
C85—C86—C81	121.9 (3)	C259—C258—C257	113.4 (19)
C85—C86—H86	119.1	C259—C258—H258	108.5
C88—C87—Si5	110.93 (19)	C258—C259—H25C	109.1
C88—C87—C90	109.0 (3)	C258—C259—H25D	109.1
C89—C87—Si5	110.1 (2)	H25C—C259—H25D	107.8
C89—C87—C88	108.1 (3)	O34—C259—C258	112 (2)
C89—C87—C90	108.7 (3)	O34—C259—H25C	109.1
C90—C87—Si5	110.0 (2)	O34—C259—H25D	109.1
C87—C88—H88A	109.5	C259—O34—H34	88 (3)
			
Si1—O1—C17—C18	167.75 (17)	C82—C81—C86—C85	−0.8 (4)
Si1—C1—C2—C3	177.2 (3)	C82—C83—C84—C85	−1.6 (5)
Si1—C1—C6—C5	−177.56 (19)	C83—C84—C85—C86	1.2 (5)
Si1—C11—C12—C13	176.1 (2)	C84—C85—C86—C81	0.0 (5)
Si1—C11—C16—C15	−176.55 (18)	C86—C81—C82—C83	0.4 (4)
O1—Si1—C1—C2	157.6 (2)	C87—Si5—O13—C97	154.7 (2)
O1—Si1—C1—C6	−24.6 (2)	C87—Si5—C81—C82	117.4 (2)
O1—Si1—C7—C8	−50.27 (19)	C87—Si5—C81—C86	−62.1 (2)
O1—Si1—C7—C9	68.17 (18)	C87—Si5—C91—C92	115.1 (2)
O1—Si1—C7—C10	−173.62 (17)	C87—Si5—C91—C96	−62.9 (2)
O1—Si1—C11—C12	−74.4 (2)	C91—Si5—O13—C97	−86.4 (2)
O1—Si1—C11—C16	102.0 (2)	C91—Si5—C81—C82	−7.0 (3)
O1—C17—C18—O2	−67.1 (3)	C91—Si5—C81—C86	173.6 (2)
O1—C17—C18—C19	55.9 (3)	C91—Si5—C87—C88	−164.3 (2)
O2—C18—C19—O3	−56.6 (3)	C91—Si5—C87—C89	76.0 (2)
C1—Si1—O1—C17	−69.9 (2)	C91—Si5—C87—C90	−43.7 (3)
C1—Si1—C7—C8	−166.02 (17)	C91—C92—C93—C94	−0.3 (5)
C1—Si1—C7—C9	−47.58 (19)	C92—C91—C96—C95	−0.7 (4)
C1—Si1—C7—C10	70.63 (19)	C92—C93—C94—C95	−0.1 (5)
C1—Si1—C11—C12	43.1 (2)	C93—C94—C95—C96	0.1 (5)
C1—Si1—C11—C16	−140.4 (2)	C94—C95—C96—C91	0.3 (4)
C1—C2—C3—C4	0.7 (5)	C96—C91—C92—C93	0.7 (4)
C2—C1—C6—C5	0.4 (4)	C97—C98—C99—O15	62.3 (3)
C2—C3—C4—C5	−0.4 (6)	Si6—C101—C102—C103	−177.0 (2)
C3—C4—C5—C6	0.0 (5)	Si6—C101—C106—C105	176.8 (2)
C4—C5—C6—C1	0.0 (4)	Si6—C111—C112—C113	−179.3 (2)
C6—C1—C2—C3	−0.7 (4)	Si6—C111—C116—C115	179.5 (2)
C7—Si1—O1—C17	172.24 (18)	Si6—O16—C117—C118	−92.6 (4)
C7—Si1—C1—C2	−89.0 (2)	Si6—O216—C217—C218	−163.6 (14)
C7—Si1—C1—C6	88.8 (2)	C101—Si6—C107—C108	54.5 (2)
C7—Si1—C11—C12	168.82 (18)	C101—Si6—C107—C109	172.7 (2)
C7—Si1—C11—C16	−14.7 (2)	C101—Si6—C107—C110	−64.4 (2)
C11—Si1—O1—C17	48.5 (2)	C101—Si6—C111—C112	−47.3 (2)
C11—Si1—C1—C2	39.3 (3)	C101—Si6—C111—C116	133.9 (2)
C11—Si1—C1—C6	−142.91 (19)	C101—Si6—O16—C117	90.8 (3)
C11—Si1—C7—C8	69.22 (19)	C101—Si6—O216—C217	74.5 (16)
C11—Si1—C7—C9	−172.34 (16)	C101—C102—C103—C104	0.0 (5)
C11—Si1—C7—C10	−54.1 (2)	C102—C101—C106—C105	−0.7 (4)
C11—C12—C13—C14	1.0 (4)	C102—C103—C104—C105	−0.3 (5)
C12—C11—C16—C15	0.0 (3)	C103—C104—C105—C106	0.1 (5)
C12—C13—C14—C15	−0.6 (4)	C104—C105—C106—C101	0.4 (4)
C13—C14—C15—C16	0.0 (4)	C106—C101—C102—C103	0.4 (4)
C14—C15—C16—C11	0.4 (4)	C107—Si6—C101—C102	95.3 (2)
C16—C11—C12—C13	−0.6 (4)	C107—Si6—C101—C106	−82.0 (2)
C17—C18—C19—O3	−178.2 (2)	C107—Si6—C111—C112	−170.2 (2)
Si2—O4—C37—C38	−132.7 (2)	C107—Si6—C111—C116	10.9 (3)
Si2—C21—C22—C23	178.1 (2)	C107—Si6—O16—C117	−152.0 (3)
Si2—C21—C26—C25	−178.5 (2)	C107—Si6—O216—C217	−169.0 (13)
Si2—C31—C32—C33	−178.8 (2)	C111—Si6—C101—C102	−31.1 (2)
Si2—C31—C36—C35	178.5 (2)	C111—Si6—C101—C106	151.5 (2)
O4—Si2—C21—C22	−105.1 (2)	C111—Si6—C107—C108	175.7 (2)
O4—Si2—C21—C26	73.5 (2)	C111—Si6—C107—C109	−66.0 (2)
O4—Si2—C27—C28	56.7 (2)	C111—Si6—C107—C110	56.8 (2)
O4—Si2—C27—C29	179.9 (2)	C111—Si6—O16—C117	−28.8 (4)
O4—Si2—C27—C30	−60.9 (2)	C111—Si6—O216—C217	−36.2 (17)
O4—Si2—C31—C32	−161.0 (2)	C111—C112—C113—C114	−0.1 (4)
O4—Si2—C31—C36	21.0 (2)	C112—C111—C116—C115	0.7 (4)
O4—C37—C38—O5	178.8 (2)	C112—C113—C114—C115	0.3 (4)
O4—C37—C38—C39	−59.2 (3)	C113—C114—C115—C116	0.0 (4)
O5—C38—C39—O6	−67.9 (3)	C114—C115—C116—C111	−0.5 (4)
C21—Si2—O4—C37	10.9 (3)	C116—C111—C112—C113	−0.3 (4)
C21—Si2—C27—C28	−66.7 (3)	O16—Si6—C101—C102	−153.0 (2)
C21—Si2—C27—C29	56.5 (2)	O16—Si6—C101—C106	29.7 (3)
C21—Si2—C27—C30	175.77 (19)	O16—Si6—C107—C108	−64.7 (2)
C21—Si2—C31—C32	−41.7 (3)	O16—Si6—C107—C109	53.6 (2)
C21—Si2—C31—C36	140.3 (2)	O16—Si6—C107—C110	176.4 (2)
C21—C22—C23—C24	0.7 (4)	O16—Si6—C111—C112	75.8 (3)
C22—C21—C26—C25	0.2 (4)	O16—Si6—C111—C116	−103.0 (2)
C22—C23—C24—C25	−0.4 (4)	O16—C117—C118—O17	173.4 (3)
C23—C24—C25—C26	0.0 (4)	O16—C117—C118—C119	−63.1 (4)
C24—C25—C26—C21	0.1 (4)	O17—C118—C119—O18	57.1 (3)
C26—C21—C22—C23	−0.5 (4)	C117—C118—C119—O18	−66.1 (3)
C27—Si2—O4—C37	−114.9 (3)	O216—Si6—C101—C102	−143.7 (7)
C27—Si2—C21—C22	15.5 (3)	O216—Si6—C101—C106	39.0 (7)
C27—Si2—C21—C26	−165.9 (2)	O216—Si6—C107—C108	−54.6 (7)
C27—Si2—C31—C32	85.4 (2)	O216—Si6—C107—C109	63.7 (7)
C27—Si2—C31—C36	−92.5 (2)	O216—Si6—C107—C110	−173.5 (7)
C31—Si2—O4—C37	128.9 (3)	O216—Si6—C111—C112	56.9 (5)
C31—Si2—C21—C22	139.6 (2)	O216—Si6—C111—C116	−121.9 (5)
C31—Si2—C21—C26	−41.8 (2)	O216—C217—C218—O217	179.0 (13)
C31—Si2—C27—C28	169.5 (2)	O216—C217—C218—C219	−56.5 (15)
C31—Si2—C27—C29	−67.2 (2)	O217—C218—C219—O218	−46.6 (18)
C31—Si2—C27—C30	52.0 (2)	C217—C218—C219—O218	−173.0 (10)
C31—C32—C33—C34	−0.1 (4)	Si7—O19—C137—C138	168.6 (2)
C32—C31—C36—C35	0.5 (4)	Si7—C131—C132—C133	179.4 (2)
C32—C33—C34—C35	1.3 (4)	Si7—C131—C136—C135	−178.9 (3)
C33—C34—C35—C36	−1.6 (4)	Si7—C121—C122—C123	179.4 (8)
C34—C35—C36—C31	0.7 (5)	Si7—C121—C126—C125	−178.8 (6)
C36—C31—C32—C33	−0.8 (4)	Si7—C161—C162—C163	175.3 (15)
C37—C38—C39—O6	172.8 (2)	Si7—C161—C166—C165	−175.2 (14)
Si3—O7—C57—C58	155.52 (19)	O19—Si7—C127—C128	175.0 (2)
Si3—C41—C42—C43	179.8 (3)	O19—Si7—C127—C129	56.8 (2)
Si3—C41—C46—C45	−179.6 (2)	O19—Si7—C127—C130	−62.7 (2)
Si3—C51—C52—C53	−179.39 (19)	O19—Si7—C131—C132	153.1 (2)
Si3—C51—C56—C55	179.7 (2)	O19—Si7—C131—C136	−26.9 (3)
O7—Si3—C41—C42	113.9 (2)	O19—Si7—C121—C122	119.3 (10)
O7—Si3—C41—C46	−65.6 (2)	O19—Si7—C121—C126	−60.1 (11)
O7—Si3—C47—C48	57.9 (2)	O19—C137—C138—O20	−80.5 (3)
O7—Si3—C47—C49	177.1 (2)	O19—C137—C138—C139	47.3 (3)
O7—Si3—C47—C50	−61.1 (3)	O20—C138—C139—O21	−57.1 (3)
O7—Si3—C51—C52	157.82 (19)	C127—Si7—O19—C137	45.9 (3)
O7—Si3—C51—C56	−22.5 (2)	C127—Si7—C131—C132	−86.7 (2)
O7—C57—C58—O8	−66.9 (3)	C127—Si7—C131—C136	93.3 (3)
O7—C57—C58—C59	56.1 (3)	C127—Si7—C121—C122	−7.3 (14)
O8—C58—C59—O9	−64.6 (3)	C127—Si7—C121—C126	173.3 (8)
C41—Si3—O7—C57	−55.5 (2)	C131—Si7—O19—C137	163.9 (3)
C41—Si3—C47—C48	−176.9 (2)	C131—Si7—C127—C128	60.9 (2)
C41—Si3—C47—C49	−57.8 (2)	C131—Si7—C127—C129	−57.3 (2)
C41—Si3—C47—C50	64.1 (3)	C131—Si7—C127—C130	−176.82 (19)
C41—Si3—C51—C52	41.3 (2)	C131—Si7—C121—C122	−130.2 (11)
C41—Si3—C51—C56	−139.1 (2)	C131—Si7—C121—C126	50.5 (10)
C41—C42—C43—C44	0.4 (5)	C131—C132—C133—C134	0.2 (5)
C42—C41—C46—C45	0.8 (4)	C132—C131—C136—C135	1.1 (5)
C42—C43—C44—C45	−0.2 (5)	C132—C133—C134—C135	−0.3 (5)
C43—C44—C45—C46	0.4 (5)	C133—C134—C135—C136	0.7 (6)
C44—C45—C46—C41	−0.7 (4)	C134—C135—C136—C131	−1.2 (6)
C46—C41—C42—C43	−0.7 (4)	C136—C131—C132—C133	−0.6 (4)
C47—Si3—O7—C57	72.2 (2)	C137—C138—C139—O21	174.9 (2)
C47—Si3—C41—C42	−11.6 (3)	C121—Si7—O19—C137	−82.2 (6)
C47—Si3—C41—C46	168.93 (19)	C121—Si7—C127—C128	−61.2 (6)
C47—Si3—C51—C52	−84.4 (2)	C121—Si7—C127—C129	−179.3 (6)
C47—Si3—C51—C56	95.3 (2)	C121—Si7—C127—C130	61.1 (6)
C51—Si3—O7—C57	−170.7 (2)	C121—Si7—C131—C132	39.7 (4)
C51—Si3—C41—C42	−134.2 (2)	C121—Si7—C131—C136	−140.3 (4)
C51—Si3—C41—C46	46.3 (2)	C121—C122—C123—C124	0.8 (15)
C51—Si3—C47—C48	−55.0 (3)	C122—C121—C126—C125	1.8 (15)
C51—Si3—C47—C49	64.1 (2)	C122—C123—C124—C125	−0.8 (17)
C51—Si3—C47—C50	−174.0 (2)	C123—C124—C125—C126	1.4 (16)
C51—C52—C53—C54	−0.3 (4)	C124—C125—C126—C121	−1.9 (12)
C52—C51—C56—C55	−0.6 (4)	C126—C121—C122—C123	−1.2 (16)
C52—C53—C54—C55	−0.7 (4)	C161—Si7—O19—C137	−76.8 (12)
C53—C54—C55—C56	1.0 (4)	C161—Si7—C127—C128	−60.8 (10)
C54—C55—C56—C51	−0.4 (4)	C161—Si7—C127—C129	−179.0 (10)
C56—C51—C52—C53	1.0 (3)	C161—Si7—C127—C130	61.5 (10)
C57—C58—C59—O9	173.7 (2)	C161—Si7—C131—C132	33.6 (6)
Si4—O10—C77—C78	−163.62 (19)	C161—Si7—C131—C136	−146.4 (6)
Si4—C61—C62—C63	173.3 (2)	C161—C162—C163—C164	−4 (3)
Si4—C61—C66—C65	−174.1 (3)	C162—C161—C166—C165	−5 (4)
Si4—C71—C72—C73	−171.8 (2)	C162—C163—C164—C165	3 (3)
Si4—C71—C76—C75	172.2 (3)	C163—C164—C165—C166	−2 (3)
O10—Si4—C61—C62	−162.8 (2)	C164—C165—C166—C161	3 (3)
O10—Si4—C61—C66	12.2 (2)	C166—C161—C162—C163	5 (4)
O10—Si4—C67—C68	−170.9 (2)	Si8—C141—C142—C143	−174.8 (3)
O10—Si4—C67—C69	67.1 (2)	Si8—C141—C146—C145	174.2 (2)
O10—Si4—C67—C70	−52.8 (2)	Si8—C151—C152—C153	174.7 (2)
O10—Si4—C71—C72	−177.7 (2)	Si8—C151—C156—C155	−173.65 (19)
O10—Si4—C71—C76	8.8 (3)	Si8—O22—C157—C158	129.9 (4)
O10—C77—C78—O11	73.0 (3)	Si8—O32—C257—C258	152 (4)
O10—C77—C78—C79	−167.9 (3)	C141—Si8—C147—C148	−50.2 (3)
O11—C78—C79—O12	−65.3 (3)	C141—Si8—C147—C149	71.3 (2)
C61—Si4—O10—C77	−165.2 (3)	C141—Si8—C147—C150	−169.29 (19)
C61—Si4—C67—C68	−58.2 (2)	C141—Si8—C151—C152	120.3 (2)
C61—Si4—C67—C69	179.71 (19)	C141—Si8—C151—C156	−64.4 (2)
C61—Si4—C67—C70	59.9 (2)	C141—Si8—O22—C157	−40.0 (4)
C61—Si4—C71—C72	72.7 (2)	C141—Si8—O32—C257	−33 (5)
C61—Si4—C71—C76	−100.8 (3)	C141—C142—C143—C144	−0.4 (5)
C61—C62—C63—C64	0.8 (5)	C142—C141—C146—C145	−1.9 (4)
C62—C61—C66—C65	1.1 (4)	C142—C143—C144—C145	−0.2 (5)
C62—C63—C64—C65	1.1 (6)	C143—C144—C145—C146	−0.3 (5)
C63—C64—C65—C66	−1.8 (6)	C144—C145—C146—C141	1.4 (4)
C64—C65—C66—C61	0.7 (5)	C146—C141—C142—C143	1.4 (4)
C66—C61—C62—C63	−1.8 (4)	C147—Si8—C141—C142	−129.3 (2)
C67—Si4—O10—C77	−46.1 (3)	C147—Si8—C141—C146	54.7 (3)
C67—Si4—C61—C62	78.8 (2)	C147—Si8—C151—C152	−111.5 (2)
C67—Si4—C61—C66	−106.2 (2)	C147—Si8—C151—C156	63.7 (2)
C67—Si4—C71—C72	−52.6 (3)	C147—Si8—O22—C157	−163.6 (3)
C67—Si4—C71—C76	134.0 (3)	C147—Si8—O32—C257	−162 (4)
C71—Si4—O10—C77	81.1 (3)	C151—Si8—C141—C142	−5.0 (3)
C71—Si4—C61—C62	−47.9 (2)	C151—Si8—C141—C146	179.0 (2)
C71—Si4—C61—C66	127.1 (2)	C151—Si8—C147—C148	−175.2 (2)
C71—Si4—C67—C68	64.8 (2)	C151—Si8—C147—C149	−53.6 (2)
C71—Si4—C67—C69	−57.3 (2)	C151—Si8—C147—C150	65.7 (2)
C71—Si4—C67—C70	−177.12 (17)	C151—Si8—O22—C157	80.8 (4)
C71—C72—C73—C74	−1.0 (5)	C151—Si8—O32—C257	82 (5)
C72—C71—C76—C75	−1.8 (5)	C151—C152—C153—C154	−0.7 (5)
C72—C73—C74—C75	0.0 (5)	C152—C151—C156—C155	1.7 (4)
C73—C74—C75—C76	0.0 (6)	C152—C153—C154—C155	1.5 (4)
C74—C75—C76—C71	0.9 (6)	C153—C154—C155—C156	−0.7 (4)
C76—C71—C72—C73	1.8 (4)	C154—C155—C156—C151	−0.9 (4)
C77—C78—C79—O12	174.0 (3)	C156—C151—C152—C153	−0.9 (4)
Si5—O13—C97—C98	−161.09 (16)	O22—Si8—C141—C142	115.2 (2)
Si5—C81—C82—C83	−179.0 (2)	O22—Si8—C141—C146	−60.8 (3)
Si5—C81—C86—C85	178.7 (2)	O22—Si8—C147—C148	69.0 (3)
Si5—C91—C92—C93	−177.4 (2)	O22—Si8—C147—C149	−169.4 (3)
Si5—C91—C96—C95	177.3 (2)	O22—Si8—C147—C150	−50.1 (3)
O13—Si5—C81—C82	−126.4 (2)	O22—Si8—C151—C152	0.0 (3)
O13—Si5—C81—C86	54.2 (2)	O22—Si8—C151—C156	175.2 (2)
O13—Si5—C87—C88	−47.4 (2)	O22—C157—C158—O23	−63.0 (4)
O13—Si5—C87—C89	−167.1 (2)	O22—C157—C158—C159	57.1 (4)
O13—Si5—C87—C90	73.2 (3)	C157—C158—C159—O24	172.8 (3)
O13—Si5—C91—C92	0.5 (2)	O23—C158—C159—O24	−65.6 (3)
O13—Si5—C91—C96	−177.5 (2)	O32—Si8—C141—C142	104.6 (12)
O13—C97—C98—O14	−62.9 (3)	O32—Si8—C141—C146	−71.3 (12)
O13—C97—C98—C99	59.6 (3)	O32—Si8—C147—C148	73.4 (16)
O14—C98—C99—O15	−174.36 (19)	O32—Si8—C147—C149	−165.0 (16)
C81—Si5—O13—C97	33.8 (2)	O32—Si8—C147—C150	−45.7 (16)
C81—Si5—C87—C88	72.1 (3)	O32—Si8—C151—C152	6.8 (13)
C81—Si5—C87—C89	−47.6 (3)	O32—Si8—C151—C156	−178.0 (13)
C81—Si5—C87—C90	−167.3 (2)	O32—C257—C258—O33	−164 (3)
C81—Si5—C91—C92	−119.9 (2)	O32—C257—C258—C259	−44 (4)
C81—Si5—C91—C96	62.0 (2)	C257—C258—C259—O34	165 (3)
C81—C82—C83—C84	0.8 (5)	O33—C258—C259—O34	−73 (3)

### 3-[(tert-Butyldiphenylsilyl)oxy]propane-1,2-diol (1) Hydrogen-bond geometry (Å, º)

**Table:**

*D*—H···*A*	*D*—H	H···*A*	*D*···*A*	*D*—H···*A*
O2—H2···O18	0.84	2.02	2.840 (3)	166
O2—H2···O218	0.84	2.06	2.796 (9)	146
O3—H3*A*···O23	0.84	1.93	2.757 (3)	167
O5—H5*A*···O9	0.84	1.82	2.659 (3)	174
O6—H6*A*···O14^i^	0.84	1.87	2.701 (3)	168
O8—H8···O6	0.84	2.06	2.812 (3)	149
O9—H9···O24	0.84	2.14	2.835 (5)	140
O11—H11···O6	0.84	1.95	2.787 (3)	177
O12—H12*G*···O24	0.84	2.08	2.867 (4)	155
O14—H14*G*···O13	0.84	2.42	2.838 (2)	111
O15—H15*H*···O21^ii^	0.84	1.85	2.679 (3)	169
O17—H17···O12	0.84	1.86	2.639 (3)	154
O18—H18···O15	0.84	1.90	2.722 (3)	164
O218—H218···O15	0.84	1.80	2.630 (10)	171
O20—H20···O5	0.84	1.99	2.785 (3)	157
O21—H21···O8	0.84	1.89	2.731 (3)	175
O23—H23···O17	0.84	1.86	2.689 (3)	171
O24—H24···O2	0.84	1.92	2.727 (5)	161
C119—H11*G*···O3	0.99	2.45	3.283 (4)	141
C138—H138···O3^i^	1.00	2.50	3.314 (3)	138

Symmetry codes: (i) *x*−1, *y*, *z*; (ii) *x*+1, *y*, *z*.

### (S)-1-O-t-butyldiphenylsilylpropane-2,3-diol (2) Crystal data

**Table:**

C_19_H_26_O_3_Si	*F*(000) = 1424
*M**_r_* = 330.49	*D*_x_ = 1.184 Mg m^−^^3^
Triclinic, *P*1	Melting point: 334 K
*a* = 14.7922 (10) Å	Mo *K*α radiation, λ = 0.71073 Å
*b* = 15.6306 (10) Å	Cell parameters from 9308 reflections
*c* = 17.2048 (11) Å	θ = 2.9–27.6°
α = 110.901 (2)°	µ = 0.14 mm^−^^1^
β = 91.705 (2)°	*T* = 173 K
γ = 92.851 (2)°	Block, colourless
*V* = 3706.8 (4) Å^3^	0.6 × 0.45 × 0.37 mm
*Z* = 8	

### (S)-1-O-t-butyldiphenylsilylpropane-2,3-diol (2) Data collection

**Table:**

Bruker PHOTON-100 CMOS diffractometer	28468 reflections with *I* > 2σ(*I*)
Radiation source: sealedtube	*R*_int_ = 0.034
ω scans	θ_max_ = 27.6°, θ_min_ = 2.8°
Absorption correction: multi-scan (SADABS; Krause *et al.*, 2015)	*h* = −19→19
*T*_min_ = 0.907, *T*_max_ = 0.950	*k* = −20→20
120838 measured reflections	*l* = −22→22
34177 independent reflections	

### (S)-1-O-t-butyldiphenylsilylpropane-2,3-diol (2) Refinement

**Table:**

Refinement on *F*^2^	Hydrogen site location: mixed
Least-squares matrix: full	H-atom parameters constrained
*R*[*F*^2^ > 2σ(*F*^2^)] = 0.046	*w* = 1/[σ^2^(*F*_o_^2^) + (0.0633*P*)^2^ + 0.5751*P*] where *P* = (*F*_o_^2^ + 2*F*_c_^2^)/3
*wR*(*F*^2^) = 0.118	(Δ/σ)_max_ = 0.001
*S* = 1.03	Δρ_max_ = 0.33 e Å^−^^3^
34177 reflections	Δρ_min_ = −0.25 e Å^−^^3^
1857 parameters	Absolute structure: Flack x determined using 11980 quotients [(*I*^+^)-(*I*^-^)]/[(*I*^+^)+(*I*^-^)] (Parsons et al., 2013
272 restraints	Absolute structure parameter: −0.008 (18)

### (S)-1-O-t-butyldiphenylsilylpropane-2,3-diol (2) Special details

**Table:**

Geometry. All esds (except the esd in the dihedral angle between two l.s. planes) are estimated using the full covariance matrix. The cell esds are taken into account individually in the estimation of esds in distances, angles and torsion angles; correlations between esds in cell parameters are only used when they are defined by crystal symmetry. An approximate (isotropic) treatment of cell esds is used for estimating esds involving l.s. planes.

### (S)-1-O-t-butyldiphenylsilylpropane-2,3-diol (2) Fractional atomic coordinates and isotropic or equivalent isotropic displacement parameters (Å^2^)

**Table:**

	*x*	*y*	*z*	*U*_iso_*/*U*_eq_	Occ. (<1)
Si1	0.61557 (5)	0.63297 (5)	0.22560 (5)	0.02855 (17)	
O1	0.66840 (14)	0.57278 (14)	0.27224 (13)	0.0346 (5)	
O2	0.68848 (16)	0.54615 (18)	0.42796 (15)	0.0452 (6)	
H2	0.705997	0.529953	0.467222	0.068*	
O3	0.7951 (2)	0.3905 (2)	0.3604 (2)	0.0606 (7)	
H3A	0.747064	0.378765	0.380569	0.091*	0.639 (6)
H3B	0.795846	0.419177	0.412029	0.091*	0.361 (6)
C1	0.5524 (2)	0.7205 (2)	0.30591 (19)	0.0340 (7)	
C2	0.5274 (3)	0.8011 (2)	0.2966 (2)	0.0483 (9)	
H2A	0.544775	0.813773	0.248951	0.058*	
C3	0.4777 (3)	0.8633 (3)	0.3555 (3)	0.0658 (12)	
H3	0.460769	0.917620	0.347794	0.079*	
C4	0.4529 (3)	0.8457 (3)	0.4257 (3)	0.0593 (11)	
H4	0.418841	0.887937	0.466264	0.071*	
C5	0.4777 (2)	0.7673 (3)	0.4366 (2)	0.0474 (8)	
H5	0.461046	0.755482	0.484906	0.057*	
C6	0.5269 (2)	0.7051 (2)	0.3775 (2)	0.0390 (7)	
H6	0.543587	0.650948	0.385867	0.047*	
C7	0.5347 (2)	0.5462 (2)	0.14620 (19)	0.0329 (6)	
C8	0.5829 (3)	0.4621 (2)	0.0930 (2)	0.0455 (8)	
H8A	0.538038	0.415754	0.056636	0.068*	
H8B	0.615110	0.436559	0.129417	0.068*	
H8C	0.626239	0.480349	0.058893	0.068*	
C9	0.4620 (2)	0.5143 (3)	0.1936 (2)	0.0460 (8)	
H9A	0.419054	0.468904	0.153671	0.069*	
H9B	0.429637	0.567102	0.227258	0.069*	
H9C	0.490955	0.486617	0.230013	0.069*	
C10	0.4867 (2)	0.5889 (3)	0.0898 (2)	0.0431 (8)	
H10G	0.437828	0.545950	0.055962	0.065*	
H10H	0.530407	0.602039	0.053152	0.065*	
H10I	0.461250	0.646110	0.124262	0.065*	
C11	0.7015 (2)	0.6934 (2)	0.18167 (19)	0.0302 (6)	
C12	0.7529 (2)	0.7704 (2)	0.2364 (2)	0.0393 (7)	
H12	0.740456	0.792358	0.293771	0.047*	
C13	0.8208 (2)	0.8154 (2)	0.2096 (2)	0.0448 (8)	
H13	0.855230	0.866499	0.248463	0.054*	
C14	0.8384 (2)	0.7856 (3)	0.1261 (2)	0.0442 (8)	
H14	0.884120	0.816929	0.106831	0.053*	
C15	0.7891 (2)	0.7102 (2)	0.0708 (2)	0.0428 (8)	
H15	0.801497	0.689047	0.013357	0.051*	
C16	0.7218 (2)	0.6651 (2)	0.0981 (2)	0.0352 (7)	
H16	0.688497	0.613306	0.058969	0.042*	
C17	0.7433 (2)	0.6106 (2)	0.3306 (2)	0.0371 (7)	
H17A	0.729334	0.671398	0.370523	0.045*	
H17B	0.797399	0.618993	0.300940	0.045*	
C18	0.7625 (2)	0.5474 (2)	0.3769 (2)	0.0402 (7)	
H18A	0.818473	0.572368	0.413819	0.048*	
C19	0.7781 (3)	0.4516 (3)	0.3180 (3)	0.0518 (9)	
H19A	0.724008	0.427300	0.279518	0.062*	
H19B	0.830299	0.454447	0.284152	0.062*	
Si2	0.38999 (6)	0.35632 (6)	0.76076 (5)	0.0347 (2)	
Si3	0.10055 (6)	0.64607 (6)	0.24174 (5)	0.03348 (18)	
O4	0.31610 (19)	0.39614 (18)	0.71207 (16)	0.0540 (7)	
O5	0.2475 (2)	0.3925 (2)	0.50721 (17)	0.0603 (7)	
H5A	0.272291	0.418354	0.477398	0.090*	
O6	0.24708 (17)	0.58801 (16)	0.60938 (16)	0.0470 (6)	
H6A	0.222646	0.634852	0.639887	0.071*	
O7	0.17269 (16)	0.60173 (17)	0.28903 (15)	0.0436 (6)	
O8	0.19451 (16)	0.60092 (19)	0.45560 (15)	0.0473 (6)	
H8	0.210019	0.575794	0.489259	0.071*	
O9	0.33162 (19)	0.4687 (2)	0.4118 (2)	0.0656 (8)	
H9	0.388231	0.478168	0.417891	0.098*	
C21	0.4449 (2)	0.2565 (2)	0.6864 (2)	0.0383 (7)	
C22	0.4248 (3)	0.1642 (2)	0.6775 (2)	0.0518 (9)	
H22	0.381759	0.150942	0.712178	0.062*	
C23	0.4654 (3)	0.0927 (3)	0.6204 (3)	0.0601 (10)	
H23A	0.449732	0.031186	0.615460	0.072*	
C24	0.5276 (3)	0.1105 (3)	0.5714 (3)	0.0622 (10)	
H24A	0.556168	0.061160	0.532476	0.075*	
C25	0.5506 (3)	0.2006 (3)	0.5770 (3)	0.0598 (10)	
H25	0.594013	0.212632	0.542022	0.072*	
C26	0.5086 (3)	0.2720 (3)	0.6348 (2)	0.0480 (9)	
H26	0.524077	0.333246	0.638913	0.058*	
C27	0.3270 (3)	0.3309 (3)	0.8451 (2)	0.0497 (9)	
C28	0.2433 (3)	0.2642 (3)	0.8079 (3)	0.0764 (14)	
H28A	0.207059	0.259276	0.853060	0.115*	
H28B	0.263080	0.203575	0.774754	0.115*	
H28C	0.206555	0.287299	0.772213	0.115*	
C29	0.3890 (4)	0.2924 (4)	0.8948 (3)	0.0764 (14)	
H29A	0.353999	0.275761	0.935316	0.115*	
H29B	0.437478	0.338793	0.924124	0.115*	
H29C	0.415617	0.237756	0.856849	0.115*	
C30	0.2927 (3)	0.4218 (3)	0.9033 (3)	0.0674 (12)	
H30A	0.260348	0.411085	0.948172	0.101*	
H30B	0.251584	0.445934	0.871540	0.101*	
H30C	0.344352	0.466251	0.927151	0.101*	
C31	0.4776 (2)	0.4533 (2)	0.8092 (2)	0.0355 (7)	
C32	0.5650 (2)	0.4409 (2)	0.8342 (2)	0.0476 (9)	
H32	0.582168	0.380688	0.826039	0.057*	
C33	0.6269 (3)	0.5145 (3)	0.8707 (3)	0.0533 (9)	
H33A	0.685887	0.504310	0.887459	0.064*	
C34	0.6044 (3)	0.6023 (3)	0.8830 (2)	0.0478 (9)	
H34A	0.647800	0.652567	0.907077	0.057*	
C35	0.5185 (3)	0.6169 (2)	0.8602 (3)	0.0529 (9)	
H35	0.501710	0.677555	0.869613	0.064*	
C36	0.4561 (3)	0.5430 (2)	0.8233 (2)	0.0462 (8)	
H36	0.397095	0.554003	0.807242	0.055*	
C37	0.2802 (3)	0.3611 (3)	0.6287 (3)	0.0555 (10)	
H37A	0.216514	0.337672	0.627291	0.067*	
H37B	0.315245	0.309367	0.595464	0.067*	
C38	0.2840 (3)	0.4340 (3)	0.5911 (2)	0.0481 (8)	
H38	0.348679	0.455308	0.590393	0.058*	
C39	0.2321 (3)	0.5149 (3)	0.6409 (2)	0.0495 (9)	
H39A	0.166564	0.496396	0.636278	0.059*	
H39B	0.252728	0.536109	0.700348	0.059*	
C41	0.0410 (2)	0.7390 (2)	0.3209 (2)	0.0376 (7)	
C42	0.0494 (3)	0.8323 (3)	0.3337 (3)	0.0599 (11)	
H42	0.088048	0.853378	0.300226	0.072*	
C43	0.0020 (4)	0.8950 (3)	0.3950 (3)	0.0744 (15)	
H43	0.008810	0.958349	0.402646	0.089*	
C44	−0.0542 (3)	0.8671 (3)	0.4442 (3)	0.0634 (12)	
H44	−0.086481	0.910565	0.485541	0.076*	
C45	−0.0636 (3)	0.7758 (3)	0.4334 (2)	0.0518 (9)	
H45	−0.102430	0.755647	0.467251	0.062*	
C46	−0.0162 (2)	0.7130 (2)	0.3729 (2)	0.0419 (8)	
H46	−0.022933	0.650048	0.366591	0.050*	
C47	0.1578 (3)	0.6825 (3)	0.1614 (2)	0.0495 (9)	
C48	0.1987 (4)	0.5973 (3)	0.0999 (3)	0.0744 (14)	
H48A	0.229375	0.614119	0.057157	0.112*	
H48B	0.150314	0.549721	0.073359	0.112*	
H48C	0.242562	0.573971	0.129996	0.112*	
C49	0.0878 (3)	0.7182 (4)	0.1151 (3)	0.0726 (13)	
H49A	0.117155	0.734754	0.071687	0.109*	
H49B	0.061952	0.772384	0.154482	0.109*	
H49C	0.039360	0.670251	0.089514	0.109*	
C50	0.2359 (3)	0.7568 (3)	0.1986 (3)	0.0706 (13)	
H50A	0.262850	0.772922	0.153858	0.106*	
H50B	0.282146	0.733200	0.226143	0.106*	
H50C	0.212228	0.811495	0.239252	0.106*	
C51	0.0160 (2)	0.5479 (2)	0.18866 (18)	0.0318 (6)	
C52	−0.0714 (2)	0.5610 (2)	0.1654 (2)	0.0380 (7)	
H52	−0.089258	0.621608	0.176580	0.046*	
C53	−0.1335 (2)	0.4869 (2)	0.1261 (2)	0.0446 (8)	
H53	−0.193097	0.497211	0.110943	0.054*	
C54	−0.1083 (3)	0.3993 (2)	0.1094 (2)	0.0457 (8)	
H54	−0.150783	0.348825	0.082734	0.055*	
C55	−0.0225 (3)	0.3839 (2)	0.1308 (2)	0.0467 (8)	
H55	−0.005344	0.322910	0.118823	0.056*	
C56	0.0396 (2)	0.4577 (2)	0.1701 (2)	0.0418 (8)	
H56	0.099169	0.446466	0.184565	0.050*	
C57	0.2420 (3)	0.6539 (3)	0.3485 (2)	0.0524 (9)	
H57A	0.219977	0.714100	0.382106	0.063*	
H57B	0.295409	0.665044	0.319221	0.063*	
C58	0.2690 (2)	0.6044 (3)	0.4045 (2)	0.0461 (8)	
H58	0.321530	0.640262	0.441863	0.055*	
C59	0.2974 (3)	0.5106 (3)	0.3569 (2)	0.0552 (10)	
H59A	0.244848	0.472443	0.322881	0.066*	
H59B	0.344784	0.514587	0.318656	0.066*	
Si4	0.37456 (6)	0.86218 (6)	0.77683 (6)	0.03321 (19)	
O10	0.41874 (16)	0.76503 (16)	0.72834 (16)	0.0457 (6)	
O11	0.4219 (2)	0.6208 (2)	0.56680 (16)	0.0578 (7)	
H11	0.370089	0.606887	0.579022	0.087*	
O12	0.5586 (2)	0.49972 (19)	0.5625 (2)	0.0663 (8)	
H12G	0.519413	0.485798	0.522853	0.099*	
C61	0.2884 (2)	0.8274 (2)	0.8391 (2)	0.0393 (7)	
C62	0.2184 (3)	0.8830 (4)	0.8747 (3)	0.0618 (10)	
H62	0.217753	0.942365	0.871275	0.074*	
C63	0.1505 (3)	0.8545 (4)	0.9144 (3)	0.0793 (12)	
H63	0.103453	0.893447	0.938107	0.095*	
C64	0.1516 (4)	0.7686 (5)	0.9194 (3)	0.0839 (12)	
H64	0.103948	0.747726	0.945399	0.101*	
C65	0.2190 (4)	0.7139 (4)	0.8882 (4)	0.0842 (13)	
H65	0.219757	0.655668	0.893925	0.101*	
C66	0.2890 (3)	0.7425 (3)	0.8467 (3)	0.0597 (11)	
H66	0.336169	0.703316	0.824157	0.072*	
C67	0.4626 (2)	0.9501 (2)	0.8451 (2)	0.0414 (8)	
C68	0.4201 (3)	1.0367 (3)	0.9009 (3)	0.0672 (13)	
H68A	0.467078	1.078189	0.939270	0.101*	
H68B	0.373305	1.019875	0.932930	0.101*	
H68C	0.392808	1.067597	0.866323	0.101*	
C69	0.5325 (3)	0.9768 (3)	0.7928 (3)	0.0579 (10)	
H69A	0.580312	1.018482	0.829653	0.087*	
H69B	0.502874	1.007501	0.759177	0.087*	
H69C	0.559050	0.921511	0.755977	0.087*	
C70	0.5096 (3)	0.9068 (3)	0.9015 (2)	0.0473 (8)	
H70A	0.557287	0.950150	0.936910	0.071*	
H70B	0.536528	0.850493	0.866998	0.071*	
H70C	0.465048	0.891969	0.936577	0.071*	
C71	0.3122 (2)	0.8996 (2)	0.6994 (2)	0.0366 (7)	
C72	0.2693 (3)	0.9814 (2)	0.7197 (3)	0.0521 (9)	
H72	0.277270	1.024784	0.774916	0.063*	
C73	0.2157 (3)	1.0020 (3)	0.6627 (3)	0.0611 (11)	
H73	0.186719	1.058177	0.678800	0.073*	
C74	0.2044 (3)	0.9403 (3)	0.5820 (3)	0.0631 (10)	
H74	0.168500	0.953849	0.541733	0.076*	
C75	0.2453 (4)	0.8598 (4)	0.5608 (3)	0.0763 (12)	
H75	0.236529	0.816417	0.505626	0.092*	
C76	0.2994 (3)	0.8400 (3)	0.6179 (2)	0.0656 (11)	
H76	0.328616	0.783888	0.600817	0.079*	
C77	0.4973 (2)	0.7387 (2)	0.6848 (2)	0.0482 (9)	
H77A	0.550854	0.751983	0.724164	0.058*	
H77B	0.506909	0.772828	0.646718	0.058*	
C78	0.4843 (2)	0.6368 (2)	0.6357 (2)	0.0432 (7)	
H78	0.460534	0.604494	0.672396	0.052*	
C79	0.5709 (3)	0.5980 (3)	0.6008 (3)	0.0650 (10)	
H79A	0.590214	0.624221	0.558912	0.078*	
H79B	0.618911	0.614593	0.645882	0.078*	
Si5	0.88899 (6)	0.87732 (6)	0.77004 (5)	0.0360 (2)	
O13	0.97184 (15)	0.81163 (15)	0.73036 (13)	0.0368 (5)	
O14	1.13413 (17)	0.7200 (2)	0.68654 (16)	0.0551 (7)	
H14G	1.115522	0.742604	0.734869	0.083*	
O15	0.93514 (15)	0.61301 (17)	0.54848 (15)	0.0422 (5)	
H15H	0.956050	0.581933	0.502834	0.063*	
C81	0.8096 (2)	0.8807 (2)	0.6847 (2)	0.0392 (7)	
C82	0.7860 (3)	0.9629 (3)	0.6773 (2)	0.0521 (9)	
H82	0.811412	1.019470	0.716235	0.062*	
C83	0.7256 (3)	0.9632 (3)	0.6136 (3)	0.0642 (11)	
H83	0.710298	1.019869	0.609663	0.077*	
C84	0.6883 (3)	0.8827 (3)	0.5568 (3)	0.0594 (11)	
H84	0.647231	0.883570	0.513635	0.071*	
C85	0.7102 (3)	0.8003 (3)	0.5620 (2)	0.0546 (10)	
H85	0.684059	0.744329	0.522583	0.066*	
C86	0.7707 (3)	0.7992 (3)	0.6249 (2)	0.0486 (8)	
H86	0.786030	0.741962	0.627421	0.058*	
C87	0.8329 (3)	0.8232 (3)	0.8404 (3)	0.0569 (9)	
C88	0.8145 (4)	0.7199 (3)	0.7953 (3)	0.0772 (13)	
H88A	0.784276	0.693475	0.832218	0.116*	
H88B	0.871941	0.691365	0.779412	0.116*	
H88C	0.775386	0.708560	0.745135	0.116*	
C89	0.7422 (3)	0.8633 (4)	0.8654 (3)	0.0762 (13)	
H89A	0.712475	0.832730	0.899577	0.114*	
H89B	0.703724	0.853773	0.815293	0.114*	
H89C	0.751721	0.929133	0.897523	0.114*	
C90	0.8941 (4)	0.8410 (5)	0.9179 (3)	0.0868 (14)	
H90A	0.863326	0.816842	0.956117	0.130*	
H90B	0.907986	0.907146	0.945477	0.130*	
H90C	0.950552	0.810345	0.901937	0.130*	
C91	0.9387 (2)	0.9961 (2)	0.8319 (2)	0.0414 (8)	
C92	1.0319 (3)	1.0137 (3)	0.8372 (2)	0.0482 (9)	
H92	1.068546	0.965727	0.807236	0.058*	
C93	1.0738 (3)	1.0983 (3)	0.8845 (3)	0.0634 (11)	
H93	1.137852	1.108179	0.886986	0.076*	
C94	1.0203 (4)	1.1684 (3)	0.9284 (3)	0.0645 (12)	
H94	1.047913	1.226828	0.961506	0.077*	
C95	0.9289 (4)	1.1539 (3)	0.9240 (2)	0.0625 (12)	
H95	0.892827	1.202247	0.954195	0.075*	
C96	0.8877 (3)	1.0696 (3)	0.8762 (2)	0.0525 (9)	
H96	0.823511	1.061164	0.873208	0.063*	
C97	1.0121 (2)	0.8006 (2)	0.6532 (2)	0.0399 (7)	
H97A	1.054618	0.854017	0.659980	0.048*	
H97B	0.964574	0.796738	0.610144	0.048*	
C98	1.0621 (2)	0.7141 (2)	0.6268 (2)	0.0398 (7)	
H98	1.089808	0.707268	0.572764	0.048*	
C99	1.0020 (2)	0.6284 (2)	0.6137 (2)	0.0440 (8)	
H99A	1.039672	0.574830	0.600024	0.053*	
H99B	0.971929	0.634526	0.665888	0.053*	
Si6	0.88543 (6)	0.36523 (7)	0.77272 (6)	0.0399 (2)	
C101	0.9447 (2)	0.2703 (3)	0.6969 (2)	0.0443 (8)	
C102	0.9536 (3)	0.1857 (3)	0.7070 (3)	0.0554 (10)	
H102	0.926651	0.175095	0.752448	0.066*	
C103	1.0004 (3)	0.1182 (3)	0.6524 (3)	0.0677 (12)	
H103	1.005342	0.061591	0.660496	0.081*	
C104	1.0406 (3)	0.1315 (4)	0.5858 (3)	0.0686 (12)	
H104	1.072978	0.084377	0.548257	0.082*	
C105	1.0333 (3)	0.2132 (4)	0.5743 (3)	0.0634 (12)	
H105	1.060998	0.222908	0.528801	0.076*	
C106	0.9859 (3)	0.2818 (3)	0.6285 (2)	0.0505 (9)	
H106	0.981066	0.337784	0.619232	0.061*	
C107	0.9692 (2)	0.4479 (3)	0.8512 (2)	0.0443 (8)	
C108	1.0420 (3)	0.4801 (3)	0.8038 (3)	0.0660 (12)	
H10A	1.083399	0.527423	0.843672	0.099*	
H10B	1.012764	0.505539	0.765722	0.099*	
H10C	1.076115	0.427906	0.771894	0.099*	
C109	0.9226 (3)	0.5327 (3)	0.9071 (3)	0.0632 (11)	
H10D	0.968035	0.576353	0.945541	0.095*	
H10E	0.877179	0.513655	0.939049	0.095*	
H10F	0.892969	0.561979	0.872303	0.095*	
C110	1.0164 (3)	0.4022 (3)	0.9052 (2)	0.0526 (9)	
H11A	1.063741	0.444994	0.941623	0.079*	
H11B	1.043692	0.346890	0.869213	0.079*	
H11C	0.971881	0.385297	0.939161	0.079*	
C111	0.7940 (2)	0.3106 (2)	0.8163 (2)	0.0382 (7)	
C112	0.7374 (2)	0.2386 (3)	0.7610 (2)	0.0490 (9)	
H112	0.747476	0.218360	0.703181	0.059*	
C113	0.6670 (3)	0.1957 (3)	0.7882 (2)	0.0507 (9)	
H113	0.629114	0.147687	0.749320	0.061*	
C114	0.6528 (2)	0.2234 (2)	0.8714 (2)	0.0453 (8)	
H114	0.605348	0.194053	0.890620	0.054*	
C115	0.7071 (2)	0.2938 (2)	0.9277 (2)	0.0439 (8)	
H115	0.696835	0.312873	0.985445	0.053*	
C116	0.7763 (2)	0.3368 (2)	0.9004 (2)	0.0383 (7)	
H116	0.812883	0.385452	0.939940	0.046*	
O16	0.8417 (6)	0.4387 (4)	0.7320 (4)	0.0349 (14)	0.639 (6)
O17	0.6631 (2)	0.3712 (2)	0.5665 (2)	0.0351 (9)	0.639 (6)
H17	0.642514	0.422361	0.573458	0.053*	0.639 (6)
O18	0.7808 (2)	0.5152 (3)	0.5617 (2)	0.0365 (10)	0.639 (6)
H18	0.823977	0.553555	0.565115	0.055*	0.639 (6)
C117	0.7545 (3)	0.4342 (4)	0.6937 (3)	0.0369 (12)	0.639 (6)
H11D	0.735307	0.497198	0.705263	0.044*	0.639 (6)
H11E	0.710334	0.402068	0.717567	0.044*	0.639 (6)
C118	0.7548 (3)	0.3847 (5)	0.6016 (4)	0.0323 (12)	0.639 (6)
H118	0.777249	0.322664	0.592105	0.039*	0.639 (6)
C119	0.8158 (4)	0.4304 (4)	0.5574 (4)	0.0369 (13)	0.639 (6)
H11F	0.877711	0.441581	0.583845	0.044*	0.639 (6)
H11G	0.819005	0.389887	0.498413	0.044*	0.639 (6)
O216	0.8400 (12)	0.4074 (7)	0.7100 (8)	0.038 (3)	0.361 (6)
O217	0.7059 (6)	0.3947 (5)	0.5278 (5)	0.052 (2)	0.361 (6)
H217	0.690666	0.339465	0.519385	0.078*	0.361 (6)
O218	0.7718 (5)	0.5805 (6)	0.5872 (5)	0.0456 (19)	0.361 (6)
H218	0.824633	0.593411	0.576466	0.068*	0.361 (6)
C217	0.7710 (6)	0.3719 (6)	0.6490 (7)	0.037 (2)	0.361 (6)
H21A	0.711475	0.378426	0.674718	0.044*	0.361 (6)
H21B	0.777906	0.305860	0.618163	0.044*	0.361 (6)
C218	0.7762 (9)	0.4253 (7)	0.5891 (8)	0.037 (2)	0.361 (6)
H21C	0.834951	0.413685	0.561083	0.044*	0.361 (6)
C219	0.7754 (7)	0.5249 (6)	0.6376 (6)	0.045 (2)	0.361 (6)
H21D	0.722331	0.535734	0.672857	0.055*	0.361 (6)
H21E	0.830591	0.544404	0.675174	0.055*	0.361 (6)
Si7	0.13650 (6)	0.15854 (6)	0.26029 (6)	0.0368 (2)	
O19	0.08991 (17)	0.25227 (17)	0.31634 (19)	0.0538 (7)	
O20	0.0589 (2)	0.3915 (2)	0.49084 (18)	0.0664 (8)	
H20	0.113762	0.382933	0.481250	0.100*	
O21	0.02271 (17)	0.52272 (16)	0.41232 (17)	0.0481 (6)	
H21	0.074511	0.549834	0.424674	0.072*	
C127	0.0525 (2)	0.0717 (3)	0.1852 (2)	0.0461 (8)	
C128	0.0994 (4)	−0.0110 (3)	0.1285 (3)	0.0765 (14)	
H12A	0.055167	−0.051781	0.086372	0.115*	
H12B	0.124836	−0.044267	0.161836	0.115*	
H12C	0.148112	0.010040	0.100996	0.115*	
C129	0.0071 (3)	0.1189 (4)	0.1309 (3)	0.0729 (14)	
H12D	−0.036825	0.075213	0.090619	0.109*	
H12E	0.053384	0.140039	0.101041	0.109*	
H12F	−0.024139	0.171585	0.166433	0.109*	
C130	−0.0219 (3)	0.0371 (3)	0.2291 (3)	0.0591 (11)	
H13A	−0.063952	−0.007357	0.187349	0.089*	
H13B	−0.054992	0.088993	0.263384	0.089*	
H13C	0.005700	0.007533	0.264718	0.089*	
C131	0.2204 (2)	0.2007 (2)	0.2014 (2)	0.0389 (7)	
C132	0.2978 (3)	0.1546 (3)	0.1727 (3)	0.0588 (10)	
H132	0.308014	0.100506	0.183927	0.071*	
C133	0.3603 (3)	0.1845 (4)	0.1286 (3)	0.0693 (12)	
H133	0.412868	0.151814	0.110610	0.083*	
C134	0.3461 (3)	0.2621 (3)	0.1108 (3)	0.0666 (12)	
H134	0.387842	0.282558	0.079236	0.080*	
C135	0.2715 (4)	0.3091 (3)	0.1389 (4)	0.0750 (14)	
H135	0.261826	0.363262	0.127612	0.090*	
C136	0.2096 (3)	0.2793 (3)	0.1837 (3)	0.0609 (11)	
H136	0.158331	0.313662	0.202913	0.073*	
C137	0.0098 (2)	0.2678 (2)	0.3592 (2)	0.0447 (8)	
H13D	0.005564	0.229115	0.393890	0.054*	
H13E	−0.042987	0.250092	0.318688	0.054*	
C138	0.0074 (2)	0.3677 (2)	0.4142 (2)	0.0444 (8)	
H138	−0.057138	0.377690	0.428925	0.053*	
C139	0.0310 (3)	0.4281 (2)	0.3665 (2)	0.0482 (8)	
H13F	0.094203	0.419049	0.349329	0.058*	
H13G	−0.008995	0.409417	0.315297	0.058*	
C121	0.1993 (15)	0.1186 (9)	0.3330 (10)	0.0376 (14)	0.570 (8)
C122	0.1834 (7)	0.0340 (6)	0.3411 (6)	0.068 (3)	0.570 (8)
H122	0.136121	−0.007136	0.307577	0.081*	0.570 (8)
C123	0.2346 (8)	0.0076 (7)	0.3968 (7)	0.084 (3)	0.570 (8)
H123	0.223936	−0.051939	0.398929	0.100*	0.570 (8)
C124	0.2984 (14)	0.0655 (10)	0.4471 (10)	0.069 (5)	0.570 (8)
H124	0.330788	0.048207	0.487163	0.083*	0.570 (8)
C125	0.3177 (6)	0.1482 (6)	0.4421 (5)	0.063 (2)	0.570 (8)
H125	0.365518	0.187823	0.476400	0.076*	0.570 (8)
C126	0.2668 (5)	0.1762 (5)	0.3855 (5)	0.057 (2)	0.570 (8)
H126	0.279286	0.235656	0.383646	0.069*	0.570 (8)
C161	0.197 (2)	0.1048 (13)	0.3299 (12)	0.0376 (14)	0.430 (8)
C162	0.2406 (7)	0.0233 (6)	0.2982 (6)	0.049 (3)	0.430 (8)
H162	0.235487	−0.010711	0.241546	0.059*	0.430 (8)
C163	0.2886 (8)	−0.0092 (5)	0.3511 (7)	0.055 (3)	0.430 (8)
H163	0.311461	−0.066748	0.331392	0.066*	0.430 (8)
C164	0.3001 (15)	0.0386 (12)	0.4336 (11)	0.048 (4)	0.430 (8)
H164	0.335539	0.016071	0.468215	0.058*	0.430 (8)
C165	0.2604 (10)	0.1190 (8)	0.4663 (6)	0.061 (3)	0.430 (8)
H165	0.262225	0.150519	0.523308	0.074*	0.430 (8)
C166	0.2111 (7)	0.1540 (5)	0.4146 (5)	0.043 (2)	0.430 (8)
H166	0.182898	0.208149	0.438377	0.051*	0.430 (8)
Si8	0.63071 (6)	0.09366 (6)	0.21977 (6)	0.0367 (2)	
C141	0.7026 (2)	0.0863 (2)	0.3084 (2)	0.0391 (7)	
C142	0.6960 (3)	0.0105 (3)	0.3320 (2)	0.0522 (9)	
H142	0.656882	−0.041022	0.300263	0.063*	
C143	0.7449 (4)	0.0080 (3)	0.4008 (3)	0.0684 (13)	
H143	0.739463	−0.045384	0.414987	0.082*	
C144	0.8007 (3)	0.0808 (3)	0.4485 (2)	0.0581 (10)	
H144	0.833108	0.078860	0.496331	0.070*	
C145	0.8096 (3)	0.1562 (3)	0.4271 (2)	0.0575 (10)	
H145	0.848671	0.207316	0.459793	0.069*	
C146	0.7620 (3)	0.1588 (3)	0.3579 (3)	0.0555 (10)	
H146	0.769873	0.211850	0.343447	0.067*	
C147	0.6945 (3)	0.1242 (3)	0.1387 (2)	0.0476 (8)	
C148	0.7573 (4)	0.2109 (3)	0.1787 (3)	0.0800 (16)	
H14A	0.782780	0.229928	0.134994	0.120*	
H14B	0.806575	0.198109	0.211507	0.120*	
H14C	0.722829	0.260194	0.215150	0.120*	
C149	0.7496 (3)	0.0455 (3)	0.0881 (3)	0.0651 (11)	
H14D	0.787573	0.065530	0.051191	0.098*	
H14E	0.708556	−0.006611	0.054587	0.098*	
H14F	0.788270	0.027043	0.125810	0.098*	
C150	0.6246 (3)	0.1431 (4)	0.0803 (3)	0.0681 (12)	
H15A	0.655775	0.156801	0.036199	0.102*	
H15B	0.590719	0.195624	0.111944	0.102*	
H15C	0.582598	0.088890	0.055255	0.102*	
C151	0.5607 (2)	−0.0168 (2)	0.1670 (2)	0.0352 (7)	
C152	0.4671 (2)	−0.0163 (3)	0.1619 (3)	0.0499 (9)	
H152	0.439187	0.039170	0.189624	0.060*	
C153	0.4128 (3)	−0.0952 (3)	0.1170 (3)	0.0577 (10)	
H153	0.348806	−0.092847	0.114658	0.069*	
C154	0.4511 (3)	−0.1750 (3)	0.0768 (2)	0.0512 (9)	
H154	0.413851	−0.228268	0.045703	0.061*	
C155	0.5436 (3)	−0.1793 (2)	0.0807 (2)	0.0438 (8)	
H155	0.570255	−0.235570	0.052891	0.053*	
C156	0.5980 (2)	−0.1012 (2)	0.1256 (2)	0.0396 (7)	
H156	0.661849	−0.104759	0.128398	0.047*	
O22	0.5669 (6)	0.1792 (5)	0.2528 (7)	0.0367 (17)	0.712 (5)
O23	0.6352 (2)	0.3223 (2)	0.40032 (19)	0.0387 (9)	0.712 (5)
H23	0.644870	0.330194	0.450846	0.058*	0.712 (5)
O24	0.5185 (18)	0.4556 (6)	0.3799 (14)	0.049 (3)	0.712 (5)
H24	0.572858	0.475589	0.384383	0.073*	0.712 (5)
C157	0.5224 (5)	0.2010 (5)	0.3285 (7)	0.0412 (17)	0.712 (5)
H15D	0.544947	0.164144	0.360473	0.049*	0.712 (5)
H15E	0.456376	0.186263	0.316731	0.049*	0.712 (5)
C158	0.5403 (3)	0.3012 (3)	0.3784 (3)	0.0360 (10)	0.712 (5)
H158	0.506344	0.316865	0.430630	0.043*	0.712 (5)
C159	0.5114 (4)	0.3618 (3)	0.3307 (3)	0.0389 (11)	0.712 (5)
H15F	0.549625	0.351272	0.282314	0.047*	0.712 (5)
H15G	0.447776	0.343689	0.309133	0.047*	0.712 (5)
O32	0.5482 (15)	0.1686 (14)	0.2607 (18)	0.035 (3)	0.288 (5)
O33	0.4371 (8)	0.3243 (8)	0.4423 (6)	0.073 (3)	0.288 (5)
H33	0.444982	0.283589	0.462618	0.109*	0.288 (5)
O34	0.520 (4)	0.4560 (16)	0.394 (3)	0.047 (7)	0.288 (5)
H34	0.566633	0.491504	0.401487	0.071*	0.288 (5)
C257	0.5002 (17)	0.1990 (13)	0.3359 (17)	0.047 (4)	0.288 (5)
H25A	0.539593	0.194773	0.381792	0.057*	0.288 (5)
H25B	0.446003	0.156670	0.329074	0.057*	0.288 (5)
C258	0.4702 (9)	0.2961 (8)	0.3609 (8)	0.053 (3)	0.288 (5)
H258	0.419912	0.296873	0.321042	0.064*	0.288 (5)
C259	0.5441 (10)	0.3636 (10)	0.3599 (11)	0.056 (3)	0.288 (5)
H25C	0.598067	0.355943	0.391634	0.067*	0.288 (5)
H25D	0.560826	0.350492	0.301653	0.067*	0.288 (5)

### (S)-1-O-t-butyldiphenylsilylpropane-2,3-diol (2) Atomic displacement parameters (Å^2^)

**Table:**

	*U*^11^	*U*^22^	*U*^33^	*U*^12^	*U*^13^	*U*^23^
Si1	0.0304 (4)	0.0278 (4)	0.0281 (4)	−0.0011 (3)	−0.0011 (3)	0.0113 (3)
O1	0.0352 (11)	0.0346 (11)	0.0369 (12)	−0.0016 (9)	−0.0064 (9)	0.0178 (10)
O2	0.0449 (13)	0.0543 (15)	0.0437 (14)	−0.0014 (11)	−0.0075 (11)	0.0276 (12)
O3	0.0475 (15)	0.0623 (17)	0.093 (2)	0.0166 (13)	0.0075 (16)	0.0512 (17)
C1	0.0363 (16)	0.0310 (15)	0.0320 (16)	−0.0047 (12)	−0.0005 (13)	0.0091 (13)
C2	0.064 (2)	0.0353 (18)	0.047 (2)	0.0072 (16)	0.0142 (18)	0.0159 (15)
C3	0.091 (3)	0.037 (2)	0.068 (3)	0.016 (2)	0.025 (2)	0.0125 (19)
C4	0.065 (3)	0.046 (2)	0.050 (2)	0.0039 (18)	0.0179 (19)	−0.0037 (18)
C5	0.046 (2)	0.053 (2)	0.0361 (18)	−0.0063 (16)	0.0053 (15)	0.0088 (16)
C6	0.0405 (17)	0.0421 (17)	0.0325 (16)	−0.0039 (14)	0.0006 (13)	0.0119 (14)
C7	0.0339 (15)	0.0303 (15)	0.0339 (16)	−0.0020 (12)	−0.0001 (12)	0.0113 (13)
C8	0.053 (2)	0.0330 (17)	0.0441 (19)	0.0014 (15)	0.0002 (16)	0.0070 (15)
C9	0.0434 (19)	0.0441 (19)	0.049 (2)	−0.0121 (15)	−0.0010 (16)	0.0169 (16)
C10	0.0394 (18)	0.050 (2)	0.0411 (19)	−0.0049 (15)	−0.0114 (15)	0.0196 (16)
C11	0.0313 (15)	0.0280 (14)	0.0339 (15)	0.0031 (12)	−0.0004 (12)	0.0142 (12)
C12	0.0450 (18)	0.0352 (17)	0.0354 (17)	−0.0049 (14)	−0.0009 (14)	0.0112 (14)
C13	0.0426 (18)	0.0385 (17)	0.054 (2)	−0.0095 (14)	−0.0021 (16)	0.0198 (16)
C14	0.0408 (18)	0.0468 (19)	0.054 (2)	−0.0024 (15)	0.0068 (16)	0.0288 (17)
C15	0.0440 (18)	0.048 (2)	0.0407 (18)	0.0031 (15)	0.0074 (15)	0.0213 (16)
C16	0.0365 (16)	0.0347 (16)	0.0343 (16)	0.0000 (13)	0.0004 (13)	0.0128 (13)
C17	0.0350 (16)	0.0395 (17)	0.0376 (17)	−0.0048 (13)	−0.0078 (13)	0.0165 (14)
C18	0.0322 (16)	0.0487 (19)	0.0462 (19)	−0.0017 (14)	−0.0062 (14)	0.0260 (16)
C19	0.050 (2)	0.051 (2)	0.064 (2)	0.0146 (17)	−0.0010 (18)	0.0312 (19)
Si2	0.0376 (5)	0.0293 (4)	0.0362 (5)	0.0013 (4)	−0.0064 (4)	0.0115 (4)
Si3	0.0362 (4)	0.0321 (4)	0.0310 (4)	−0.0016 (3)	−0.0018 (3)	0.0107 (3)
O4	0.0633 (17)	0.0463 (14)	0.0455 (14)	0.0178 (12)	−0.0205 (12)	0.0082 (12)
O5	0.0712 (19)	0.0585 (17)	0.0473 (15)	0.0053 (14)	−0.0138 (14)	0.0155 (13)
O6	0.0477 (14)	0.0405 (13)	0.0506 (14)	0.0138 (11)	−0.0018 (11)	0.0127 (11)
O7	0.0414 (13)	0.0448 (13)	0.0400 (13)	0.0023 (10)	−0.0096 (10)	0.0106 (11)
O8	0.0437 (13)	0.0617 (16)	0.0355 (13)	0.0139 (11)	0.0026 (10)	0.0146 (11)
O9	0.0458 (15)	0.084 (2)	0.077 (2)	0.0226 (15)	0.0080 (14)	0.0384 (17)
C21	0.0378 (17)	0.0323 (16)	0.0421 (18)	0.0020 (13)	−0.0110 (14)	0.0111 (14)
C22	0.066 (2)	0.0347 (16)	0.051 (2)	0.0034 (16)	−0.0124 (17)	0.0117 (15)
C23	0.073 (2)	0.0389 (17)	0.059 (2)	0.0109 (16)	−0.0165 (18)	0.0065 (15)
C24	0.064 (2)	0.0471 (18)	0.056 (2)	0.0217 (17)	−0.0205 (17)	−0.0059 (16)
C25	0.050 (2)	0.062 (2)	0.051 (2)	0.0089 (18)	−0.0044 (17)	0.0002 (18)
C26	0.046 (2)	0.0392 (18)	0.048 (2)	0.0006 (15)	−0.0048 (16)	0.0033 (16)
C27	0.053 (2)	0.047 (2)	0.048 (2)	−0.0079 (17)	0.0046 (17)	0.0180 (17)
C28	0.070 (3)	0.057 (3)	0.087 (3)	−0.024 (2)	0.017 (3)	0.010 (2)
C29	0.096 (4)	0.087 (3)	0.062 (3)	−0.005 (3)	−0.003 (3)	0.048 (3)
C30	0.060 (3)	0.064 (3)	0.061 (3)	−0.011 (2)	0.014 (2)	0.002 (2)
C31	0.0413 (17)	0.0296 (15)	0.0361 (17)	0.0013 (13)	−0.0022 (13)	0.0128 (13)
C32	0.0442 (19)	0.0345 (17)	0.059 (2)	0.0030 (15)	−0.0059 (17)	0.0113 (16)
C33	0.0396 (19)	0.052 (2)	0.060 (2)	−0.0009 (16)	−0.0083 (17)	0.0124 (18)
C34	0.055 (2)	0.0403 (19)	0.0412 (19)	−0.0135 (16)	−0.0012 (16)	0.0081 (15)
C35	0.067 (3)	0.0320 (18)	0.059 (2)	−0.0023 (17)	−0.0054 (19)	0.0167 (17)
C36	0.049 (2)	0.0316 (17)	0.056 (2)	0.0024 (15)	−0.0086 (17)	0.0154 (16)
C37	0.053 (2)	0.047 (2)	0.059 (2)	0.0014 (17)	−0.0222 (19)	0.0119 (18)
C38	0.0436 (19)	0.049 (2)	0.048 (2)	0.0044 (16)	−0.0099 (16)	0.0126 (16)
C39	0.0412 (19)	0.061 (2)	0.049 (2)	0.0146 (17)	0.0000 (16)	0.0224 (18)
C41	0.0424 (17)	0.0339 (16)	0.0342 (16)	0.0014 (13)	−0.0073 (13)	0.0104 (13)
C42	0.100 (3)	0.0367 (19)	0.045 (2)	0.005 (2)	0.004 (2)	0.0158 (17)
C43	0.133 (5)	0.035 (2)	0.053 (2)	0.022 (2)	0.005 (3)	0.0110 (18)
C44	0.080 (3)	0.062 (3)	0.039 (2)	0.028 (2)	−0.002 (2)	0.0034 (19)
C45	0.047 (2)	0.060 (2)	0.0411 (19)	0.0133 (17)	0.0010 (16)	0.0086 (17)
C46	0.0417 (18)	0.0419 (18)	0.0393 (18)	0.0044 (14)	−0.0012 (14)	0.0111 (15)
C47	0.054 (2)	0.050 (2)	0.044 (2)	−0.0105 (17)	0.0029 (16)	0.0173 (17)
C48	0.089 (3)	0.070 (3)	0.058 (3)	−0.002 (3)	0.036 (2)	0.014 (2)
C49	0.083 (3)	0.099 (4)	0.054 (2)	−0.008 (3)	−0.002 (2)	0.052 (3)
C50	0.067 (3)	0.072 (3)	0.076 (3)	−0.026 (2)	0.006 (2)	0.036 (3)
C51	0.0371 (16)	0.0315 (15)	0.0282 (15)	0.0029 (12)	0.0029 (12)	0.0123 (12)
C52	0.0393 (17)	0.0327 (16)	0.0414 (18)	0.0025 (13)	−0.0005 (14)	0.0127 (14)
C53	0.0393 (18)	0.046 (2)	0.0446 (19)	−0.0052 (15)	−0.0072 (15)	0.0138 (16)
C54	0.055 (2)	0.0401 (18)	0.0389 (18)	−0.0133 (16)	−0.0056 (16)	0.0136 (15)
C55	0.069 (2)	0.0262 (16)	0.0433 (19)	0.0003 (15)	−0.0044 (17)	0.0113 (14)
C56	0.0477 (19)	0.0342 (17)	0.0432 (19)	0.0069 (14)	−0.0048 (15)	0.0136 (15)
C57	0.044 (2)	0.064 (2)	0.050 (2)	−0.0108 (18)	−0.0145 (16)	0.0237 (19)
C58	0.0333 (17)	0.062 (2)	0.0442 (19)	−0.0041 (15)	−0.0071 (14)	0.0217 (17)
C59	0.044 (2)	0.082 (3)	0.050 (2)	0.0230 (19)	0.0156 (17)	0.032 (2)
Si4	0.0316 (4)	0.0309 (4)	0.0381 (5)	0.0047 (3)	0.0016 (3)	0.0132 (4)
O10	0.0388 (12)	0.0356 (12)	0.0558 (15)	0.0073 (10)	0.0077 (11)	0.0069 (11)
O11	0.0599 (16)	0.0583 (17)	0.0477 (15)	0.0121 (14)	−0.0105 (13)	0.0098 (13)
O12	0.0529 (16)	0.0500 (15)	0.073 (2)	0.0181 (13)	−0.0119 (14)	−0.0070 (14)
C61	0.0381 (17)	0.0475 (19)	0.0355 (17)	0.0020 (14)	−0.0019 (13)	0.0193 (15)
C62	0.050 (2)	0.086 (3)	0.054 (2)	0.0107 (19)	0.0161 (17)	0.029 (2)
C63	0.061 (2)	0.116 (3)	0.067 (2)	0.007 (2)	0.0193 (19)	0.039 (2)
C64	0.067 (2)	0.132 (3)	0.076 (2)	−0.005 (2)	0.012 (2)	0.065 (2)
C65	0.073 (3)	0.113 (3)	0.099 (3)	−0.013 (2)	−0.004 (2)	0.081 (3)
C66	0.050 (2)	0.066 (3)	0.079 (3)	−0.0024 (19)	−0.001 (2)	0.047 (2)
C67	0.0417 (18)	0.0335 (16)	0.0483 (19)	0.0027 (14)	−0.0080 (15)	0.0145 (15)
C68	0.069 (3)	0.041 (2)	0.070 (3)	0.0117 (19)	−0.030 (2)	−0.0054 (19)
C69	0.055 (2)	0.054 (2)	0.070 (3)	−0.0190 (19)	−0.012 (2)	0.031 (2)
C70	0.0419 (19)	0.052 (2)	0.050 (2)	0.0039 (16)	−0.0069 (16)	0.0219 (17)
C71	0.0327 (16)	0.0408 (17)	0.0412 (18)	0.0005 (13)	0.0031 (13)	0.0207 (15)
C72	0.062 (2)	0.0329 (17)	0.058 (2)	−0.0007 (16)	−0.0182 (19)	0.0137 (16)
C73	0.062 (2)	0.043 (2)	0.082 (3)	−0.0019 (18)	−0.027 (2)	0.030 (2)
C74	0.072 (2)	0.078 (3)	0.049 (2)	0.010 (2)	−0.0083 (18)	0.0362 (19)
C75	0.091 (3)	0.092 (3)	0.0409 (19)	0.032 (2)	−0.0026 (19)	0.0144 (19)
C76	0.081 (3)	0.077 (3)	0.0375 (19)	0.036 (2)	0.0052 (18)	0.0149 (18)
C77	0.0378 (18)	0.0453 (19)	0.054 (2)	0.0079 (15)	0.0076 (16)	0.0076 (17)
C78	0.0369 (15)	0.0429 (16)	0.0446 (17)	0.0095 (13)	−0.0004 (13)	0.0087 (14)
C79	0.0496 (19)	0.0527 (19)	0.074 (2)	0.0100 (16)	0.0034 (17)	−0.0001 (17)
Si5	0.0319 (4)	0.0450 (5)	0.0327 (4)	0.0115 (4)	0.0061 (3)	0.0145 (4)
O13	0.0377 (12)	0.0406 (12)	0.0333 (11)	0.0125 (10)	0.0084 (9)	0.0128 (10)
O14	0.0413 (13)	0.0647 (17)	0.0442 (14)	0.0197 (12)	−0.0055 (11)	−0.0007 (13)
O15	0.0347 (11)	0.0451 (13)	0.0423 (13)	0.0068 (10)	0.0026 (10)	0.0094 (11)
C81	0.0329 (16)	0.0496 (19)	0.0387 (18)	0.0104 (14)	0.0061 (13)	0.0189 (15)
C82	0.055 (2)	0.052 (2)	0.048 (2)	0.0070 (17)	−0.0104 (17)	0.0173 (17)
C83	0.066 (3)	0.064 (3)	0.069 (3)	0.008 (2)	−0.017 (2)	0.032 (2)
C84	0.053 (2)	0.075 (3)	0.055 (2)	−0.005 (2)	−0.0178 (19)	0.032 (2)
C85	0.053 (2)	0.061 (2)	0.047 (2)	−0.0098 (18)	−0.0101 (17)	0.0189 (19)
C86	0.046 (2)	0.053 (2)	0.049 (2)	0.0028 (16)	−0.0019 (16)	0.0213 (17)
C87	0.0439 (18)	0.088 (2)	0.0551 (19)	0.0142 (17)	0.0165 (15)	0.0434 (18)
C88	0.084 (3)	0.078 (3)	0.084 (3)	0.004 (2)	0.029 (2)	0.045 (2)
C89	0.056 (2)	0.105 (3)	0.077 (3)	0.019 (2)	0.032 (2)	0.039 (3)
C90	0.074 (3)	0.142 (4)	0.068 (3)	−0.010 (3)	−0.004 (2)	0.070 (3)
C91	0.0494 (19)	0.0458 (19)	0.0293 (16)	0.0176 (15)	0.0040 (14)	0.0116 (14)
C92	0.050 (2)	0.0404 (19)	0.052 (2)	0.0098 (16)	0.0071 (17)	0.0131 (16)
C93	0.066 (3)	0.051 (2)	0.071 (3)	−0.001 (2)	0.002 (2)	0.020 (2)
C94	0.102 (4)	0.040 (2)	0.049 (2)	0.009 (2)	−0.001 (2)	0.0127 (18)
C95	0.090 (3)	0.052 (2)	0.042 (2)	0.032 (2)	0.007 (2)	0.0093 (18)
C96	0.059 (2)	0.057 (2)	0.0393 (19)	0.0251 (19)	0.0073 (17)	0.0107 (17)
C97	0.0402 (17)	0.0435 (18)	0.0359 (17)	0.0083 (14)	0.0114 (14)	0.0124 (14)
C98	0.0314 (16)	0.0463 (18)	0.0356 (17)	0.0076 (13)	0.0008 (13)	0.0066 (14)
C99	0.0440 (18)	0.0410 (18)	0.0422 (19)	0.0129 (15)	0.0005 (15)	0.0080 (15)
Si6	0.0335 (5)	0.0557 (6)	0.0377 (5)	−0.0032 (4)	−0.0061 (4)	0.0270 (4)
C101	0.0387 (18)	0.059 (2)	0.0359 (18)	−0.0031 (16)	−0.0064 (14)	0.0197 (16)
C102	0.060 (2)	0.062 (2)	0.046 (2)	0.0007 (19)	−0.0009 (18)	0.0225 (19)
C103	0.076 (3)	0.063 (3)	0.066 (3)	0.009 (2)	0.000 (2)	0.026 (2)
C104	0.072 (3)	0.079 (3)	0.049 (2)	0.019 (2)	0.003 (2)	0.014 (2)
C105	0.058 (2)	0.097 (4)	0.039 (2)	0.014 (2)	0.0019 (18)	0.029 (2)
C106	0.048 (2)	0.069 (3)	0.0409 (19)	0.0046 (18)	−0.0012 (16)	0.0277 (18)
C107	0.0377 (18)	0.0468 (19)	0.050 (2)	−0.0047 (15)	−0.0065 (15)	0.0212 (17)
C108	0.050 (2)	0.078 (3)	0.077 (3)	−0.019 (2)	−0.007 (2)	0.039 (3)
C109	0.063 (3)	0.046 (2)	0.076 (3)	0.0002 (19)	−0.001 (2)	0.017 (2)
C110	0.044 (2)	0.064 (2)	0.045 (2)	−0.0019 (18)	−0.0136 (16)	0.0163 (19)
C111	0.0346 (16)	0.0455 (18)	0.0369 (17)	−0.0010 (14)	−0.0030 (13)	0.0185 (15)
C112	0.0446 (19)	0.065 (2)	0.0368 (18)	−0.0076 (17)	−0.0010 (15)	0.0192 (17)
C113	0.047 (2)	0.051 (2)	0.050 (2)	−0.0116 (17)	−0.0055 (17)	0.0157 (17)
C114	0.0410 (18)	0.0420 (19)	0.057 (2)	0.0015 (15)	0.0085 (16)	0.0227 (17)
C115	0.0457 (19)	0.047 (2)	0.0401 (18)	0.0096 (15)	0.0094 (15)	0.0163 (15)
C116	0.0380 (17)	0.0383 (17)	0.0377 (17)	0.0020 (13)	−0.0010 (13)	0.0129 (14)
O16	0.035 (2)	0.043 (4)	0.031 (3)	0.000 (3)	−0.007 (3)	0.018 (3)
O17	0.0316 (18)	0.0410 (19)	0.0323 (19)	−0.0054 (15)	−0.0038 (14)	0.0144 (16)
O18	0.0282 (17)	0.045 (3)	0.042 (2)	0.0015 (15)	0.0018 (14)	0.0227 (18)
C117	0.038 (3)	0.045 (3)	0.032 (3)	0.008 (2)	0.001 (2)	0.018 (2)
C118	0.027 (3)	0.039 (3)	0.034 (3)	0.004 (2)	0.002 (2)	0.016 (3)
C119	0.031 (3)	0.052 (3)	0.038 (3)	0.005 (2)	0.003 (2)	0.028 (3)
O216	0.050 (5)	0.030 (6)	0.031 (6)	−0.002 (5)	−0.010 (5)	0.007 (4)
O217	0.062 (5)	0.041 (4)	0.047 (4)	0.004 (3)	−0.022 (4)	0.011 (3)
O218	0.053 (4)	0.044 (5)	0.050 (4)	0.002 (3)	−0.004 (3)	0.030 (4)
C217	0.041 (5)	0.030 (5)	0.038 (6)	−0.003 (4)	−0.015 (4)	0.014 (5)
C218	0.042 (6)	0.032 (5)	0.039 (6)	−0.009 (5)	−0.010 (5)	0.019 (5)
C219	0.069 (7)	0.033 (5)	0.034 (5)	0.001 (4)	−0.008 (4)	0.013 (4)
Si7	0.0353 (5)	0.0287 (4)	0.0490 (5)	0.0049 (3)	0.0025 (4)	0.0167 (4)
O19	0.0446 (14)	0.0354 (13)	0.0820 (19)	0.0110 (11)	0.0234 (13)	0.0193 (13)
O20	0.0688 (19)	0.079 (2)	0.0510 (16)	0.0098 (17)	−0.0077 (14)	0.0232 (15)
O21	0.0489 (14)	0.0357 (12)	0.0565 (15)	0.0070 (10)	0.0114 (12)	0.0114 (11)
C127	0.0434 (19)	0.047 (2)	0.052 (2)	−0.0028 (15)	−0.0109 (16)	0.0245 (17)
C128	0.086 (3)	0.059 (3)	0.063 (3)	−0.007 (2)	−0.010 (2)	−0.002 (2)
C129	0.059 (3)	0.093 (4)	0.086 (3)	−0.020 (2)	−0.027 (2)	0.061 (3)
C130	0.057 (2)	0.051 (2)	0.073 (3)	−0.0178 (19)	−0.012 (2)	0.031 (2)
C131	0.0376 (17)	0.0351 (16)	0.0452 (18)	0.0050 (13)	0.0014 (14)	0.0154 (14)
C132	0.059 (2)	0.063 (3)	0.069 (3)	0.025 (2)	0.017 (2)	0.037 (2)
C133	0.057 (3)	0.085 (3)	0.076 (3)	0.021 (2)	0.025 (2)	0.037 (3)
C134	0.062 (3)	0.081 (3)	0.062 (3)	−0.012 (2)	0.014 (2)	0.033 (2)
C135	0.079 (3)	0.064 (3)	0.103 (4)	0.007 (2)	0.022 (3)	0.054 (3)
C136	0.054 (2)	0.047 (2)	0.094 (3)	0.0112 (18)	0.018 (2)	0.038 (2)
C137	0.0402 (18)	0.0434 (19)	0.053 (2)	−0.0020 (14)	0.0033 (15)	0.0209 (16)
C138	0.0409 (18)	0.0465 (19)	0.0446 (19)	0.0027 (15)	0.0028 (15)	0.0149 (16)
C139	0.060 (2)	0.0357 (18)	0.049 (2)	0.0110 (16)	0.0081 (17)	0.0133 (15)
C121	0.041 (2)	0.030 (4)	0.043 (2)	0.003 (3)	0.0003 (16)	0.015 (2)
C122	0.081 (6)	0.056 (5)	0.073 (6)	−0.016 (4)	−0.037 (5)	0.038 (4)
C123	0.100 (8)	0.087 (7)	0.084 (7)	−0.017 (6)	−0.036 (7)	0.062 (6)
C124	0.090 (9)	0.077 (13)	0.046 (6)	0.023 (10)	−0.010 (6)	0.025 (7)
C125	0.053 (4)	0.069 (5)	0.049 (4)	0.002 (4)	−0.016 (4)	−0.001 (4)
C126	0.055 (5)	0.048 (4)	0.060 (5)	0.004 (3)	−0.004 (4)	0.009 (3)
C161	0.041 (2)	0.030 (4)	0.043 (2)	0.003 (3)	0.0003 (16)	0.015 (2)
C162	0.056 (6)	0.034 (4)	0.048 (5)	0.017 (4)	−0.014 (5)	0.003 (4)
C163	0.064 (6)	0.022 (4)	0.070 (7)	0.009 (4)	−0.022 (5)	0.007 (4)
C164	0.052 (7)	0.034 (6)	0.061 (10)	0.006 (5)	−0.018 (7)	0.021 (6)
C165	0.102 (10)	0.044 (6)	0.037 (5)	0.013 (6)	−0.007 (5)	0.014 (4)
C166	0.064 (6)	0.027 (4)	0.032 (4)	0.019 (4)	0.001 (4)	0.003 (3)
Si8	0.0431 (5)	0.0319 (4)	0.0348 (4)	−0.0038 (4)	−0.0066 (4)	0.0132 (4)
C141	0.0411 (18)	0.0399 (17)	0.0361 (17)	−0.0007 (14)	−0.0020 (14)	0.0142 (14)
C142	0.071 (3)	0.0408 (19)	0.044 (2)	−0.0014 (17)	−0.0139 (18)	0.0174 (16)
C143	0.106 (4)	0.051 (2)	0.053 (2)	0.009 (2)	−0.017 (2)	0.026 (2)
C144	0.070 (3)	0.065 (3)	0.0380 (19)	0.019 (2)	−0.0094 (18)	0.0169 (18)
C145	0.055 (2)	0.069 (3)	0.044 (2)	−0.010 (2)	−0.0130 (18)	0.0182 (19)
C146	0.062 (2)	0.056 (2)	0.053 (2)	−0.0192 (19)	−0.0149 (19)	0.0300 (19)
C147	0.054 (2)	0.047 (2)	0.0427 (19)	−0.0132 (16)	−0.0107 (16)	0.0207 (16)
C148	0.101 (4)	0.071 (3)	0.068 (3)	−0.048 (3)	−0.010 (3)	0.032 (2)
C149	0.063 (3)	0.075 (3)	0.061 (3)	−0.004 (2)	0.014 (2)	0.028 (2)
C150	0.073 (3)	0.089 (3)	0.057 (3)	−0.009 (3)	−0.012 (2)	0.046 (3)
C151	0.0379 (16)	0.0331 (15)	0.0363 (16)	0.0002 (13)	−0.0016 (13)	0.0151 (13)
C152	0.0410 (19)	0.0419 (19)	0.065 (2)	0.0044 (15)	0.0030 (17)	0.0169 (18)
C153	0.0373 (19)	0.050 (2)	0.084 (3)	−0.0061 (16)	−0.0035 (19)	0.025 (2)
C154	0.059 (2)	0.0385 (19)	0.054 (2)	−0.0172 (16)	−0.0125 (18)	0.0178 (17)
C155	0.063 (2)	0.0289 (16)	0.0403 (18)	0.0031 (15)	−0.0037 (16)	0.0135 (14)
C156	0.0409 (17)	0.0369 (17)	0.0399 (17)	0.0046 (14)	−0.0047 (14)	0.0130 (14)
O22	0.047 (3)	0.029 (3)	0.035 (3)	−0.004 (2)	0.001 (2)	0.013 (2)
O23	0.0341 (16)	0.0443 (18)	0.0359 (17)	0.0018 (13)	−0.0033 (13)	0.0126 (14)
O24	0.040 (4)	0.039 (4)	0.061 (9)	0.006 (3)	−0.003 (5)	0.010 (4)
C157	0.036 (4)	0.038 (3)	0.048 (3)	0.004 (2)	0.008 (3)	0.012 (2)
C158	0.030 (2)	0.040 (2)	0.036 (2)	0.0078 (18)	0.0075 (17)	0.0104 (18)
C159	0.038 (3)	0.037 (2)	0.036 (3)	0.010 (2)	−0.005 (2)	0.006 (2)
O32	0.053 (8)	0.025 (5)	0.034 (5)	0.001 (5)	0.007 (6)	0.018 (4)
O33	0.076 (7)	0.079 (7)	0.058 (6)	0.029 (6)	0.021 (5)	0.014 (5)
O34	0.039 (9)	0.034 (8)	0.046 (12)	0.013 (7)	−0.010 (8)	−0.012 (7)
C257	0.052 (7)	0.044 (5)	0.040 (6)	0.009 (5)	0.006 (6)	0.007 (5)
C258	0.051 (5)	0.048 (5)	0.050 (5)	0.017 (4)	0.002 (5)	0.004 (4)
C259	0.045 (6)	0.050 (6)	0.055 (6)	0.017 (5)	−0.009 (5)	−0.003 (6)

### (S)-1-O-t-butyldiphenylsilylpropane-2,3-diol (2) Geometric parameters (Å, º)

**Table:**

Si1—O1	1.648 (2)	C86—H86	0.9500
Si1—C1	1.873 (3)	C87—C88	1.528 (7)
Si1—C7	1.886 (3)	C87—C89	1.518 (6)
Si1—C11	1.878 (3)	C87—C90	1.520 (6)
O1—C17	1.424 (4)	C88—H88A	0.9800
O2—H2	0.8400	C88—H88B	0.9800
O2—C18	1.428 (4)	C88—H88C	0.9800
O3—H3A	0.8400	C89—H89A	0.9800
O3—H3B	0.8400	C89—H89B	0.9800
O3—C19	1.420 (4)	C89—H89C	0.9800
C1—C2	1.390 (5)	C90—H90A	0.9800
C1—C6	1.396 (4)	C90—H90B	0.9800
C2—H2A	0.9500	C90—H90C	0.9800
C2—C3	1.387 (5)	C91—C92	1.386 (5)
C3—H3	0.9500	C91—C96	1.402 (5)
C3—C4	1.387 (6)	C92—H92	0.9500
C4—H4	0.9500	C92—C93	1.384 (6)
C4—C5	1.369 (6)	C93—H93	0.9500
C5—H5	0.9500	C93—C94	1.388 (6)
C5—C6	1.384 (5)	C94—H94	0.9500
C6—H6	0.9500	C94—C95	1.356 (7)
C7—C8	1.532 (5)	C95—H95	0.9500
C7—C9	1.535 (5)	C95—C96	1.378 (6)
C7—C10	1.536 (4)	C96—H96	0.9500
C8—H8A	0.9800	C97—H97A	0.9900
C8—H8B	0.9800	C97—H97B	0.9900
C8—H8C	0.9800	C97—C98	1.505 (5)
C9—H9A	0.9800	C98—H98	1.0000
C9—H9B	0.9800	C98—C99	1.514 (5)
C9—H9C	0.9800	C99—H99A	0.9900
C10—H10G	0.9800	C99—H99B	0.9900
C10—H10H	0.9800	Si6—C101	1.868 (4)
C10—H10I	0.9800	Si6—C107	1.877 (4)
C11—C12	1.404 (4)	Si6—C111	1.878 (3)
C11—C16	1.392 (4)	Si6—O16	1.687 (7)
C12—H12	0.9500	Si6—O216	1.598 (14)
C12—C13	1.379 (5)	C101—C102	1.405 (6)
C13—H13	0.9500	C101—C106	1.404 (5)
C13—C14	1.382 (5)	C102—H102	0.9500
C14—H14	0.9500	C102—C103	1.371 (6)
C14—C15	1.379 (5)	C103—H103	0.9500
C15—H15	0.9500	C103—C104	1.381 (7)
C15—C16	1.380 (5)	C104—H104	0.9500
C16—H16	0.9500	C104—C105	1.369 (7)
C17—H17A	0.9900	C105—H105	0.9500
C17—H17B	0.9900	C105—C106	1.381 (6)
C17—C18	1.507 (4)	C106—H106	0.9500
C18—H18A	1.0000	C107—C108	1.539 (6)
C18—C19	1.513 (5)	C107—C109	1.542 (6)
C19—H19A	0.9900	C107—C110	1.531 (5)
C19—H19B	0.9900	C108—H10A	0.9800
Si2—O4	1.634 (2)	C108—H10B	0.9800
Si2—C21	1.869 (3)	C108—H10C	0.9800
Si2—C27	1.894 (4)	C109—H10D	0.9800
Si2—C31	1.874 (3)	C109—H10E	0.9800
Si3—O7	1.645 (2)	C109—H10F	0.9800
Si3—C41	1.878 (3)	C110—H11A	0.9800
Si3—C47	1.880 (4)	C110—H11B	0.9800
Si3—C51	1.869 (3)	C110—H11C	0.9800
O4—C37	1.416 (4)	C111—C112	1.402 (5)
O5—H5A	0.8400	C111—C116	1.392 (5)
O5—C38	1.430 (4)	C112—H112	0.9500
O6—H6A	0.8400	C112—C113	1.391 (5)
O6—C39	1.440 (5)	C113—H113	0.9500
O7—C57	1.422 (4)	C113—C114	1.366 (5)
O8—H8	0.8400	C114—H114	0.9500
O8—C58	1.441 (4)	C114—C115	1.377 (5)
O9—H9	0.8400	C115—H115	0.9500
O9—C59	1.423 (5)	C115—C116	1.381 (5)
C21—C22	1.412 (5)	C116—H116	0.9500
C21—C26	1.384 (5)	O16—C117	1.419 (9)
C22—H22	0.9500	O17—H17	0.8400
C22—C23	1.376 (6)	O17—C118	1.442 (6)
C23—H23A	0.9500	O18—H18	0.8400
C23—C24	1.350 (7)	O18—C119	1.425 (7)
C24—H24A	0.9500	C117—H11D	0.9900
C24—C25	1.400 (7)	C117—H11E	0.9900
C25—H25	0.9500	C117—C118	1.495 (7)
C25—C26	1.391 (5)	C118—H118	1.0000
C26—H26	0.9500	C118—C119	1.504 (8)
C27—C28	1.542 (6)	C119—H11F	0.9900
C27—C29	1.521 (6)	C119—H11G	0.9900
C27—C30	1.537 (6)	O216—C217	1.385 (16)
C28—H28A	0.9800	O217—H217	0.8400
C28—H28B	0.9800	O217—C218	1.394 (11)
C28—H28C	0.9800	O218—H218	0.8400
C29—H29A	0.9800	O218—C219	1.432 (10)
C29—H29B	0.9800	C217—H21A	0.9900
C29—H29C	0.9800	C217—H21B	0.9900
C30—H30A	0.9800	C217—C218	1.542 (15)
C30—H30B	0.9800	C218—H21C	1.0000
C30—H30C	0.9800	C218—C219	1.482 (13)
C31—C32	1.395 (5)	C219—H21D	0.9900
C31—C36	1.389 (4)	C219—H21E	0.9900
C32—H32	0.9500	Si7—O19	1.638 (2)
C32—C33	1.379 (5)	Si7—C127	1.881 (4)
C33—H33A	0.9500	Si7—C131	1.863 (4)
C33—C34	1.372 (6)	Si7—C121	1.834 (14)
C34—H34A	0.9500	Si7—C161	1.917 (19)
C34—C35	1.373 (6)	O19—C137	1.400 (4)
C35—H35	0.9500	O20—H20	0.8400
C35—C36	1.387 (5)	O20—C138	1.422 (4)
C36—H36	0.9500	O21—H21	0.8400
C37—H37A	0.9900	O21—C139	1.419 (4)
C37—H37B	0.9900	C127—C128	1.527 (6)
C37—C38	1.496 (6)	C127—C129	1.541 (5)
C38—H38	1.0000	C127—C130	1.536 (5)
C38—C39	1.512 (5)	C128—H12A	0.9800
C39—H39A	0.9900	C128—H12B	0.9800
C39—H39B	0.9900	C128—H12C	0.9800
C41—C42	1.394 (5)	C129—H12D	0.9800
C41—C46	1.398 (5)	C129—H12E	0.9800
C42—H42	0.9500	C129—H12F	0.9800
C42—C43	1.393 (6)	C130—H13A	0.9800
C43—H43	0.9500	C130—H13B	0.9800
C43—C44	1.367 (7)	C130—H13C	0.9800
C44—H44	0.9500	C131—C132	1.392 (5)
C44—C45	1.371 (6)	C131—C136	1.382 (5)
C45—H45	0.9500	C132—H132	0.9500
C45—C46	1.385 (5)	C132—C133	1.379 (6)
C46—H46	0.9500	C133—H133	0.9500
C47—C48	1.540 (6)	C133—C134	1.377 (7)
C47—C49	1.531 (6)	C134—H134	0.9500
C47—C50	1.544 (6)	C134—C135	1.361 (7)
C48—H48A	0.9800	C135—H135	0.9500
C48—H48B	0.9800	C135—C136	1.380 (6)
C48—H48C	0.9800	C136—H136	0.9500
C49—H49A	0.9800	C137—H13D	0.9900
C49—H49B	0.9800	C137—H13E	0.9900
C49—H49C	0.9800	C137—C138	1.513 (5)
C50—H50A	0.9800	C138—H138	1.0000
C50—H50B	0.9800	C138—C139	1.491 (5)
C50—H50C	0.9800	C139—H13F	0.9900
C51—C52	1.389 (4)	C139—H13G	0.9900
C51—C56	1.395 (4)	C121—C122	1.387 (16)
C52—H52	0.9500	C121—C126	1.379 (16)
C52—C53	1.392 (5)	C122—H122	0.9500
C53—H53	0.9500	C122—C123	1.390 (10)
C53—C54	1.369 (5)	C123—H123	0.9500
C54—H54	0.9500	C123—C124	1.330 (15)
C54—C55	1.368 (5)	C124—H124	0.9500
C55—H55	0.9500	C124—C125	1.344 (15)
C55—C56	1.390 (5)	C125—H125	0.9500
C56—H56	0.9500	C125—C126	1.414 (10)
C57—H57A	0.9900	C126—H126	0.9500
C57—H57B	0.9900	C161—C162	1.391 (18)
C57—C58	1.493 (5)	C161—C166	1.387 (17)
C58—H58	1.0000	C162—H162	0.9274
C58—C59	1.490 (6)	C162—C163	1.385 (11)
C59—H59A	0.9900	C163—H163	0.9267
C59—H59B	0.9900	C163—C164	1.348 (17)
Si4—O10	1.630 (2)	C164—H164	0.9500
Si4—C61	1.871 (3)	C164—C165	1.353 (15)
Si4—C67	1.882 (3)	C165—H165	0.9267
Si4—C71	1.871 (3)	C165—C166	1.403 (11)
O10—C77	1.401 (4)	C166—H166	0.9260
O11—H11	0.8400	Si8—C141	1.874 (3)
O11—C78	1.421 (4)	Si8—C147	1.890 (4)
O12—H12G	0.8400	Si8—C151	1.878 (3)
O12—C79	1.438 (5)	Si8—O22	1.618 (8)
C61—C62	1.396 (5)	Si8—O32	1.722 (16)
C61—C66	1.379 (5)	C141—C142	1.383 (5)
C62—H62	0.9500	C141—C146	1.397 (5)
C62—C63	1.376 (6)	C142—H142	0.9500
C63—H63	0.9500	C142—C143	1.382 (6)
C63—C64	1.376 (8)	C143—H143	0.9500
C64—H64	0.9500	C143—C144	1.360 (6)
C64—C65	1.343 (8)	C144—H144	0.9500
C65—H65	0.9500	C144—C145	1.357 (6)
C65—C66	1.416 (6)	C145—H145	0.9500
C66—H66	0.9500	C145—C146	1.379 (5)
C67—C68	1.531 (5)	C146—H146	0.9500
C67—C69	1.527 (6)	C147—C148	1.531 (5)
C67—C70	1.537 (5)	C147—C149	1.522 (6)
C68—H68A	0.9800	C147—C150	1.532 (5)
C68—H68B	0.9800	C148—H14A	0.9800
C68—H68C	0.9800	C148—H14B	0.9800
C69—H69A	0.9800	C148—H14C	0.9800
C69—H69B	0.9800	C149—H14D	0.9800
C69—H69C	0.9800	C149—H14E	0.9800
C70—H70A	0.9800	C149—H14F	0.9800
C70—H70B	0.9800	C150—H15A	0.9800
C70—H70C	0.9800	C150—H15B	0.9800
C71—C72	1.392 (5)	C150—H15C	0.9800
C71—C76	1.380 (5)	C151—C152	1.385 (5)
C72—H72	0.9500	C151—C156	1.405 (4)
C72—C73	1.378 (5)	C152—H152	0.9500
C73—H73	0.9500	C152—C153	1.395 (5)
C73—C74	1.378 (6)	C153—H153	0.9500
C74—H74	0.9500	C153—C154	1.355 (6)
C74—C75	1.357 (7)	C154—H154	0.9500
C75—H75	0.9500	C154—C155	1.374 (5)
C75—C76	1.377 (6)	C155—H155	0.9500
C76—H76	0.9500	C155—C156	1.389 (5)
C77—H77A	0.9900	C156—H156	0.9500
C77—H77B	0.9900	O22—C157	1.415 (9)
C77—C78	1.512 (5)	O23—H23	0.8400
C78—H78	1.0000	O23—C158	1.434 (5)
C78—C79	1.495 (5)	O24—H24	0.8400
C79—H79A	0.9900	O24—C159	1.404 (11)
C79—H79B	0.9900	C157—H15D	0.9900
Si5—O13	1.642 (2)	C157—H15E	0.9900
Si5—C81	1.870 (3)	C157—C158	1.499 (8)
Si5—C87	1.896 (4)	C158—H158	1.0000
Si5—C91	1.878 (4)	C158—C159	1.526 (7)
O13—C97	1.428 (4)	C159—H15F	0.9900
O14—H14G	0.8400	C159—H15G	0.9900
O14—C98	1.434 (4)	O32—C257	1.433 (19)
O15—H15H	0.8400	O33—H33	0.8400
O15—C99	1.418 (4)	O33—C258	1.420 (14)
C81—C82	1.395 (5)	O34—H34	0.8400
C81—C86	1.404 (5)	O34—C259	1.42 (2)
C82—H82	0.9500	C257—H25A	0.9900
C82—C83	1.395 (5)	C257—H25B	0.9900
C83—H83	0.9500	C257—C258	1.516 (19)
C83—C84	1.365 (6)	C258—H258	1.0000
C84—H84	0.9500	C258—C259	1.486 (17)
C84—C85	1.375 (6)	C259—H25C	0.9900
C85—H85	0.9500	C259—H25D	0.9900
C85—C86	1.388 (5)		
			
O1—Si1—C1	107.79 (13)	C87—C88—H88B	109.5
O1—Si1—C7	104.33 (13)	C87—C88—H88C	109.5
O1—Si1—C11	109.19 (12)	H88A—C88—H88B	109.5
C1—Si1—C7	110.89 (14)	H88A—C88—H88C	109.5
C1—Si1—C11	109.09 (13)	H88B—C88—H88C	109.5
C11—Si1—C7	115.21 (14)	C87—C89—H89A	109.5
C17—O1—Si1	122.53 (19)	C87—C89—H89B	109.5
C18—O2—H2	109.5	C87—C89—H89C	109.5
C19—O3—H3A	109.5	H89A—C89—H89B	109.5
C19—O3—H3B	109.5	H89A—C89—H89C	109.5
C2—C1—Si1	122.5 (3)	H89B—C89—H89C	109.5
C2—C1—C6	117.5 (3)	C87—C90—H90A	109.5
C6—C1—Si1	120.0 (2)	C87—C90—H90B	109.5
C1—C2—H2A	119.3	C87—C90—H90C	109.5
C3—C2—C1	121.4 (4)	H90A—C90—H90B	109.5
C3—C2—H2A	119.3	H90A—C90—H90C	109.5
C2—C3—H3	120.2	H90B—C90—H90C	109.5
C4—C3—C2	119.6 (4)	C92—C91—Si5	119.3 (3)
C4—C3—H3	120.2	C92—C91—C96	116.4 (3)
C3—C4—H4	120.1	C96—C91—Si5	124.3 (3)
C5—C4—C3	119.9 (4)	C91—C92—H92	118.7
C5—C4—H4	120.1	C93—C92—C91	122.6 (4)
C4—C5—H5	119.8	C93—C92—H92	118.7
C4—C5—C6	120.4 (4)	C92—C93—H93	120.6
C6—C5—H5	119.8	C92—C93—C94	118.7 (4)
C1—C6—H6	119.4	C94—C93—H93	120.6
C5—C6—C1	121.1 (3)	C93—C94—H94	119.9
C5—C6—H6	119.4	C95—C94—C93	120.2 (4)
C8—C7—Si1	111.6 (2)	C95—C94—H94	119.9
C8—C7—C9	108.3 (3)	C94—C95—H95	119.7
C8—C7—C10	109.9 (3)	C94—C95—C96	120.7 (4)
C9—C7—Si1	107.8 (2)	C96—C95—H95	119.7
C9—C7—C10	107.9 (3)	C91—C96—H96	119.3
C10—C7—Si1	111.3 (2)	C95—C96—C91	121.3 (4)
C7—C8—H8A	109.5	C95—C96—H96	119.3
C7—C8—H8B	109.5	O13—C97—H97A	109.9
C7—C8—H8C	109.5	O13—C97—H97B	109.9
H8A—C8—H8B	109.5	O13—C97—C98	108.8 (3)
H8A—C8—H8C	109.5	H97A—C97—H97B	108.3
H8B—C8—H8C	109.5	C98—C97—H97A	109.9
C7—C9—H9A	109.5	C98—C97—H97B	109.9
C7—C9—H9B	109.5	O14—C98—C97	110.8 (3)
C7—C9—H9C	109.5	O14—C98—H98	107.8
H9A—C9—H9B	109.5	O14—C98—C99	108.7 (3)
H9A—C9—H9C	109.5	C97—C98—H98	107.8
H9B—C9—H9C	109.5	C97—C98—C99	113.6 (3)
C7—C10—H10G	109.5	C99—C98—H98	107.8
C7—C10—H10H	109.5	O15—C99—C98	111.5 (3)
C7—C10—H10I	109.5	O15—C99—H99A	109.3
H10G—C10—H10H	109.5	O15—C99—H99B	109.3
H10G—C10—H10I	109.5	C98—C99—H99A	109.3
H10H—C10—H10I	109.5	C98—C99—H99B	109.3
C12—C11—Si1	118.8 (2)	H99A—C99—H99B	108.0
C16—C11—Si1	124.7 (2)	C101—Si6—C107	110.49 (16)
C16—C11—C12	116.4 (3)	C101—Si6—C111	107.11 (16)
C11—C12—H12	118.9	C107—Si6—C111	115.82 (16)
C13—C12—C11	122.3 (3)	O16—Si6—C101	114.4 (3)
C13—C12—H12	118.9	O16—Si6—C107	98.5 (3)
C12—C13—H13	120.2	O16—Si6—C111	110.6 (3)
C12—C13—C14	119.6 (3)	O216—Si6—C101	99.7 (5)
C14—C13—H13	120.2	O216—Si6—C107	113.3 (5)
C13—C14—H14	120.2	O216—Si6—C111	109.1 (7)
C15—C14—C13	119.5 (3)	C102—C101—Si6	122.1 (3)
C15—C14—H14	120.2	C106—C101—Si6	121.1 (3)
C14—C15—H15	119.7	C106—C101—C102	116.7 (4)
C14—C15—C16	120.5 (3)	C101—C102—H102	119.4
C16—C15—H15	119.7	C103—C102—C101	121.2 (4)
C11—C16—H16	119.2	C103—C102—H102	119.4
C15—C16—C11	121.7 (3)	C102—C103—H103	119.5
C15—C16—H16	119.2	C102—C103—C104	120.9 (4)
O1—C17—H17A	109.7	C104—C103—H103	119.5
O1—C17—H17B	109.7	C103—C104—H104	120.3
O1—C17—C18	109.9 (3)	C105—C104—C103	119.3 (4)
H17A—C17—H17B	108.2	C105—C104—H104	120.3
C18—C17—H17A	109.7	C104—C105—H105	119.7
C18—C17—H17B	109.7	C104—C105—C106	120.5 (4)
O2—C18—C17	108.6 (3)	C106—C105—H105	119.7
O2—C18—H18A	108.5	C101—C106—H106	119.3
O2—C18—C19	110.9 (3)	C105—C106—C101	121.4 (4)
C17—C18—H18A	108.5	C105—C106—H106	119.3
C17—C18—C19	111.6 (3)	C108—C107—Si6	108.2 (3)
C19—C18—H18A	108.5	C108—C107—C109	108.3 (3)
O3—C19—C18	112.6 (3)	C109—C107—Si6	110.9 (3)
O3—C19—H19A	109.1	C110—C107—Si6	111.4 (3)
O3—C19—H19B	109.1	C110—C107—C108	108.1 (3)
C18—C19—H19A	109.1	C110—C107—C109	109.8 (3)
C18—C19—H19B	109.1	C107—C108—H10A	109.5
H19A—C19—H19B	107.8	C107—C108—H10B	109.5
O4—Si2—C21	111.13 (14)	C107—C108—H10C	109.5
O4—Si2—C27	106.00 (18)	H10A—C108—H10B	109.5
O4—Si2—C31	105.16 (14)	H10A—C108—H10C	109.5
C21—Si2—C27	114.98 (17)	H10B—C108—H10C	109.5
C21—Si2—C31	109.21 (15)	C107—C109—H10D	109.5
C31—Si2—C27	109.87 (16)	C107—C109—H10E	109.5
O7—Si3—C41	109.92 (13)	C107—C109—H10F	109.5
O7—Si3—C47	110.83 (16)	H10D—C109—H10E	109.5
O7—Si3—C51	103.09 (13)	H10D—C109—H10F	109.5
C41—Si3—C47	114.58 (17)	H10E—C109—H10F	109.5
C51—Si3—C41	108.31 (14)	C107—C110—H11A	109.5
C51—Si3—C47	109.45 (15)	C107—C110—H11B	109.5
C37—O4—Si2	131.2 (2)	C107—C110—H11C	109.5
C38—O5—H5A	109.5	H11A—C110—H11B	109.5
C39—O6—H6A	109.5	H11A—C110—H11C	109.5
C57—O7—Si3	124.1 (2)	H11B—C110—H11C	109.5
C58—O8—H8	109.5	C112—C111—Si6	118.6 (3)
C59—O9—H9	109.5	C116—C111—Si6	125.0 (3)
C22—C21—Si2	124.4 (3)	C116—C111—C112	116.4 (3)
C26—C21—Si2	119.2 (3)	C111—C112—H112	118.9
C26—C21—C22	116.4 (3)	C113—C112—C111	122.1 (3)
C21—C22—H22	118.8	C113—C112—H112	118.9
C23—C22—C21	122.4 (4)	C112—C113—H113	120.3
C23—C22—H22	118.8	C114—C113—C112	119.3 (3)
C22—C23—H23A	120.3	C114—C113—H113	120.3
C24—C23—C22	119.5 (4)	C113—C114—H114	119.9
C24—C23—H23A	120.3	C113—C114—C115	120.3 (3)
C23—C24—H24A	119.5	C115—C114—H114	119.9
C23—C24—C25	121.0 (4)	C114—C115—H115	119.9
C25—C24—H24A	119.5	C114—C115—C116	120.3 (3)
C24—C25—H25	120.6	C116—C115—H115	119.9
C26—C25—C24	118.7 (4)	C111—C116—H116	119.2
C26—C25—H25	120.6	C115—C116—C111	121.7 (3)
C21—C26—C25	122.0 (4)	C115—C116—H116	119.2
C21—C26—H26	119.0	C117—O16—Si6	128.8 (6)
C25—C26—H26	119.0	C118—O17—H17	109.5
C28—C27—Si2	111.1 (3)	C119—O18—H18	109.5
C29—C27—Si2	111.0 (3)	O16—C117—H11D	109.4
C29—C27—C28	110.3 (4)	O16—C117—H11E	109.4
C29—C27—C30	109.4 (4)	O16—C117—C118	111.1 (5)
C30—C27—Si2	107.4 (3)	H11D—C117—H11E	108.0
C30—C27—C28	107.4 (4)	C118—C117—H11D	109.4
C27—C28—H28A	109.5	C118—C117—H11E	109.4
C27—C28—H28B	109.5	O17—C118—C117	109.2 (4)
C27—C28—H28C	109.5	O17—C118—H118	107.3
H28A—C28—H28B	109.5	O17—C118—C119	111.0 (5)
H28A—C28—H28C	109.5	C117—C118—H118	107.3
H28B—C28—H28C	109.5	C117—C118—C119	114.3 (5)
C27—C29—H29A	109.5	C119—C118—H118	107.3
C27—C29—H29B	109.5	O18—C119—C118	109.3 (5)
C27—C29—H29C	109.5	O18—C119—H11F	109.8
H29A—C29—H29B	109.5	O18—C119—H11G	109.8
H29A—C29—H29C	109.5	C118—C119—H11F	109.8
H29B—C29—H29C	109.5	C118—C119—H11G	109.8
C27—C30—H30A	109.5	H11F—C119—H11G	108.3
C27—C30—H30B	109.5	C217—O216—Si6	130.8 (10)
C27—C30—H30C	109.5	C218—O217—H217	109.5
H30A—C30—H30B	109.5	C219—O218—H218	109.5
H30A—C30—H30C	109.5	O216—C217—H21A	110.1
H30B—C30—H30C	109.5	O216—C217—H21B	110.1
C32—C31—Si2	123.2 (2)	O216—C217—C218	108.2 (9)
C36—C31—Si2	119.9 (3)	H21A—C217—H21B	108.4
C36—C31—C32	116.9 (3)	C218—C217—H21A	110.1
C31—C32—H32	119.4	C218—C217—H21B	110.1
C33—C32—C31	121.2 (3)	O217—C218—C217	110.7 (10)
C33—C32—H32	119.4	O217—C218—H21C	108.2
C32—C33—H33A	119.6	O217—C218—C219	112.2 (8)
C34—C33—C32	120.9 (4)	C217—C218—H21C	108.2
C34—C33—H33A	119.6	C219—C218—C217	109.3 (9)
C33—C34—H34A	120.3	C219—C218—H21C	108.2
C33—C34—C35	119.3 (3)	O218—C219—C218	113.8 (9)
C35—C34—H34A	120.3	O218—C219—H21D	108.8
C34—C35—H35	120.0	O218—C219—H21E	108.8
C34—C35—C36	120.0 (3)	C218—C219—H21D	108.8
C36—C35—H35	120.0	C218—C219—H21E	108.8
C31—C36—H36	119.1	H21D—C219—H21E	107.7
C35—C36—C31	121.8 (3)	O19—Si7—C127	112.58 (16)
C35—C36—H36	119.1	O19—Si7—C131	103.02 (14)
O4—C37—H37A	109.5	O19—Si7—C121	107.0 (5)
O4—C37—H37B	109.5	O19—Si7—C161	111.1 (6)
O4—C37—C38	110.9 (3)	C127—Si7—C161	110.1 (7)
H37A—C37—H37B	108.0	C131—Si7—C127	109.67 (16)
C38—C37—H37A	109.5	C131—Si7—C161	110.2 (10)
C38—C37—H37B	109.5	C121—Si7—C127	115.7 (5)
O5—C38—C37	107.0 (3)	C121—Si7—C131	108.0 (8)
O5—C38—H38	109.0	C137—O19—Si7	132.8 (2)
O5—C38—C39	111.8 (3)	C138—O20—H20	109.5
C37—C38—H38	109.0	C139—O21—H21	109.5
C37—C38—C39	111.0 (3)	C128—C127—Si7	111.3 (3)
C39—C38—H38	109.0	C128—C127—C129	108.6 (4)
O6—C39—C38	109.3 (3)	C128—C127—C130	108.4 (3)
O6—C39—H39A	109.8	C129—C127—Si7	107.4 (3)
O6—C39—H39B	109.8	C130—C127—Si7	112.8 (3)
C38—C39—H39A	109.8	C130—C127—C129	108.2 (3)
C38—C39—H39B	109.8	C127—C128—H12A	109.5
H39A—C39—H39B	108.3	C127—C128—H12B	109.5
C42—C41—Si3	126.1 (3)	C127—C128—H12C	109.5
C42—C41—C46	116.5 (3)	H12A—C128—H12B	109.5
C46—C41—Si3	117.5 (2)	H12A—C128—H12C	109.5
C41—C42—H42	119.6	H12B—C128—H12C	109.5
C43—C42—C41	120.8 (4)	C127—C129—H12D	109.5
C43—C42—H42	119.6	C127—C129—H12E	109.5
C42—C43—H43	119.4	C127—C129—H12F	109.5
C44—C43—C42	121.1 (4)	H12D—C129—H12E	109.5
C44—C43—H43	119.4	H12D—C129—H12F	109.5
C43—C44—H44	120.3	H12E—C129—H12F	109.5
C43—C44—C45	119.4 (4)	C127—C130—H13A	109.5
C45—C44—H44	120.3	C127—C130—H13B	109.5
C44—C45—H45	120.1	C127—C130—H13C	109.5
C44—C45—C46	119.9 (4)	H13A—C130—H13B	109.5
C46—C45—H45	120.1	H13A—C130—H13C	109.5
C41—C46—H46	118.9	H13B—C130—H13C	109.5
C45—C46—C41	122.3 (4)	C132—C131—Si7	121.7 (3)
C45—C46—H46	118.9	C136—C131—Si7	122.2 (3)
C48—C47—Si3	107.4 (3)	C136—C131—C132	116.1 (3)
C48—C47—C50	107.5 (4)	C131—C132—H132	118.8
C49—C47—Si3	109.5 (3)	C133—C132—C131	122.5 (4)
C49—C47—C48	109.8 (4)	C133—C132—H132	118.8
C49—C47—C50	109.3 (4)	C132—C133—H133	120.2
C50—C47—Si3	113.3 (3)	C134—C133—C132	119.6 (4)
C47—C48—H48A	109.5	C134—C133—H133	120.2
C47—C48—H48B	109.5	C133—C134—H134	120.4
C47—C48—H48C	109.5	C135—C134—C133	119.2 (4)
H48A—C48—H48B	109.5	C135—C134—H134	120.4
H48A—C48—H48C	109.5	C134—C135—H135	119.5
H48B—C48—H48C	109.5	C134—C135—C136	120.9 (4)
C47—C49—H49A	109.5	C136—C135—H135	119.5
C47—C49—H49B	109.5	C131—C136—H136	119.1
C47—C49—H49C	109.5	C135—C136—C131	121.7 (4)
H49A—C49—H49B	109.5	C135—C136—H136	119.1
H49A—C49—H49C	109.5	O19—C137—H13D	109.5
H49B—C49—H49C	109.5	O19—C137—H13E	109.5
C47—C50—H50A	109.5	O19—C137—C138	110.8 (3)
C47—C50—H50B	109.5	H13D—C137—H13E	108.1
C47—C50—H50C	109.5	C138—C137—H13D	109.5
H50A—C50—H50B	109.5	C138—C137—H13E	109.5
H50A—C50—H50C	109.5	O20—C138—C137	113.6 (3)
H50B—C50—H50C	109.5	O20—C138—H138	106.3
C52—C51—Si3	122.1 (2)	O20—C138—C139	113.3 (3)
C52—C51—C56	117.4 (3)	C137—C138—H138	106.3
C56—C51—Si3	120.5 (2)	C139—C138—C137	110.5 (3)
C51—C52—H52	119.4	C139—C138—H138	106.3
C51—C52—C53	121.2 (3)	O21—C139—C138	113.4 (3)
C53—C52—H52	119.4	O21—C139—H13F	108.9
C52—C53—H53	120.1	O21—C139—H13G	108.9
C54—C53—C52	119.8 (3)	C138—C139—H13F	108.9
C54—C53—H53	120.1	C138—C139—H13G	108.9
C53—C54—H54	119.8	H13F—C139—H13G	107.7
C53—C54—C55	120.4 (3)	C122—C121—Si7	125.1 (11)
C55—C54—H54	119.8	C126—C121—Si7	118.9 (11)
C54—C55—H55	120.1	C126—C121—C122	116.0 (11)
C54—C55—C56	119.9 (3)	C121—C122—H122	119.0
C56—C55—H55	120.1	C121—C122—C123	122.0 (9)
C51—C56—H56	119.4	C123—C122—H122	119.0
C55—C56—C51	121.2 (3)	C122—C123—H123	119.9
C55—C56—H56	119.4	C124—C123—C122	120.2 (9)
O7—C57—H57A	109.5	C124—C123—H123	119.9
O7—C57—H57B	109.5	C123—C124—H124	119.6
O7—C57—C58	110.7 (3)	C123—C124—C125	120.8 (12)
H57A—C57—H57B	108.1	C125—C124—H124	119.6
C58—C57—H57A	109.5	C124—C125—H125	120.1
C58—C57—H57B	109.5	C124—C125—C126	119.9 (9)
O8—C58—C57	108.7 (3)	C126—C125—H125	120.1
O8—C58—H58	108.3	C121—C126—C125	121.0 (9)
O8—C58—C59	111.2 (3)	C121—C126—H126	119.5
C57—C58—H58	108.3	C125—C126—H126	119.5
C59—C58—C57	112.0 (3)	C162—C161—Si7	122.9 (13)
C59—C58—H58	108.3	C166—C161—Si7	119.7 (11)
O9—C59—C58	110.7 (3)	C166—C161—C162	116.7 (14)
O9—C59—H59A	109.5	C161—C162—H162	119.9
O9—C59—H59B	109.5	C163—C162—C161	120.4 (11)
C58—C59—H59A	109.5	C163—C162—H162	119.6
C58—C59—H59B	109.5	C162—C163—H163	120.8
H59A—C59—H59B	108.1	C164—C163—C162	122.1 (10)
O10—Si4—C61	101.61 (15)	C164—C163—H163	117.0
O10—Si4—C67	111.15 (14)	C163—C164—H164	120.5
O10—Si4—C71	109.39 (15)	C163—C164—C165	118.9 (14)
C61—Si4—C67	112.05 (16)	C165—C164—H164	120.5
C61—Si4—C71	107.63 (14)	C164—C165—H165	121.0
C71—Si4—C67	114.22 (15)	C164—C165—C166	120.5 (11)
C77—O10—Si4	135.4 (2)	C166—C165—H165	118.5
C78—O11—H11	109.5	C161—C166—C165	121.1 (10)
C79—O12—H12G	109.5	C161—C166—H166	119.5
C62—C61—Si4	121.7 (3)	C165—C166—H166	119.1
C66—C61—Si4	120.4 (3)	C141—Si8—C147	115.33 (16)
C66—C61—C62	117.9 (4)	C141—Si8—C151	110.33 (14)
C61—C62—H62	119.1	C151—Si8—C147	108.96 (15)
C63—C62—C61	121.9 (5)	O22—Si8—C141	110.2 (4)
C63—C62—H62	119.1	O22—Si8—C147	100.8 (4)
C62—C63—H63	120.5	O22—Si8—C151	110.9 (3)
C62—C63—C64	119.0 (5)	O32—Si8—C141	108.1 (10)
C64—C63—H63	120.5	O32—Si8—C147	111.8 (8)
C63—C64—H64	119.5	O32—Si8—C151	101.4 (8)
C65—C64—C63	121.0 (5)	C142—C141—Si8	121.8 (3)
C65—C64—H64	119.5	C142—C141—C146	115.7 (3)
C64—C65—H65	119.8	C146—C141—Si8	122.3 (3)
C64—C65—C66	120.3 (5)	C141—C142—H142	119.2
C66—C65—H65	119.8	C143—C142—C141	121.5 (4)
C61—C66—C65	119.8 (5)	C143—C142—H142	119.2
C61—C66—H66	120.1	C142—C143—H143	119.5
C65—C66—H66	120.1	C144—C143—C142	121.1 (4)
C68—C67—Si4	111.9 (2)	C144—C143—H143	119.5
C68—C67—C70	108.0 (3)	C143—C144—H144	120.4
C69—C67—Si4	111.0 (3)	C145—C144—C143	119.2 (4)
C69—C67—C68	109.0 (3)	C145—C144—H144	120.4
C69—C67—C70	109.7 (3)	C144—C145—H145	120.0
C70—C67—Si4	107.2 (2)	C144—C145—C146	120.1 (4)
C67—C68—H68A	109.5	C146—C145—H145	120.0
C67—C68—H68B	109.5	C141—C146—H146	118.8
C67—C68—H68C	109.5	C145—C146—C141	122.4 (4)
H68A—C68—H68B	109.5	C145—C146—H146	118.8
H68A—C68—H68C	109.5	C148—C147—Si8	111.4 (3)
H68B—C68—H68C	109.5	C148—C147—C150	108.3 (4)
C67—C69—H69A	109.5	C149—C147—Si8	111.0 (3)
C67—C69—H69B	109.5	C149—C147—C148	109.4 (4)
C67—C69—H69C	109.5	C149—C147—C150	108.9 (3)
H69A—C69—H69B	109.5	C150—C147—Si8	107.8 (3)
H69A—C69—H69C	109.5	C147—C148—H14A	109.5
H69B—C69—H69C	109.5	C147—C148—H14B	109.5
C67—C70—H70A	109.5	C147—C148—H14C	109.5
C67—C70—H70B	109.5	H14A—C148—H14B	109.5
C67—C70—H70C	109.5	H14A—C148—H14C	109.5
H70A—C70—H70B	109.5	H14B—C148—H14C	109.5
H70A—C70—H70C	109.5	C147—C149—H14D	109.5
H70B—C70—H70C	109.5	C147—C149—H14E	109.5
C72—C71—Si4	124.3 (3)	C147—C149—H14F	109.5
C76—C71—Si4	119.3 (3)	H14D—C149—H14E	109.5
C76—C71—C72	116.1 (3)	H14D—C149—H14F	109.5
C71—C72—H72	118.7	H14E—C149—H14F	109.5
C73—C72—C71	122.5 (4)	C147—C150—H15A	109.5
C73—C72—H72	118.7	C147—C150—H15B	109.5
C72—C73—H73	120.3	C147—C150—H15C	109.5
C72—C73—C74	119.4 (4)	H15A—C150—H15B	109.5
C74—C73—H73	120.3	H15A—C150—H15C	109.5
C73—C74—H74	120.4	H15B—C150—H15C	109.5
C75—C74—C73	119.2 (4)	C152—C151—Si8	119.7 (3)
C75—C74—H74	120.4	C152—C151—C156	116.5 (3)
C74—C75—H75	119.4	C156—C151—Si8	123.5 (2)
C74—C75—C76	121.1 (4)	C151—C152—H152	119.2
C76—C75—H75	119.4	C151—C152—C153	121.7 (3)
C71—C76—H76	119.2	C153—C152—H152	119.2
C75—C76—C71	121.6 (4)	C152—C153—H153	119.9
C75—C76—H76	119.2	C154—C153—C152	120.2 (4)
O10—C77—H77A	110.3	C154—C153—H153	119.9
O10—C77—H77B	110.3	C153—C154—H154	119.8
O10—C77—C78	107.2 (3)	C153—C154—C155	120.3 (3)
H77A—C77—H77B	108.5	C155—C154—H154	119.8
C78—C77—H77A	110.3	C154—C155—H155	120.2
C78—C77—H77B	110.3	C154—C155—C156	119.7 (3)
O11—C78—C77	109.6 (3)	C156—C155—H155	120.2
O11—C78—H78	109.7	C151—C156—H156	119.2
O11—C78—C79	106.7 (3)	C155—C156—C151	121.5 (3)
C77—C78—H78	109.7	C155—C156—H156	119.2
C79—C78—C77	111.5 (3)	C157—O22—Si8	122.6 (7)
C79—C78—H78	109.7	C158—O23—H23	109.5
O12—C79—C78	110.1 (3)	C159—O24—H24	109.5
O12—C79—H79A	109.6	O22—C157—H15D	109.8
O12—C79—H79B	109.6	O22—C157—H15E	109.8
C78—C79—H79A	109.6	O22—C157—C158	109.3 (6)
C78—C79—H79B	109.6	H15D—C157—H15E	108.3
H79A—C79—H79B	108.2	C158—C157—H15D	109.8
O13—Si5—C81	109.99 (13)	C158—C157—H15E	109.8
O13—Si5—C87	104.68 (16)	O23—C158—C157	110.3 (4)
O13—Si5—C91	108.68 (14)	O23—C158—H158	108.9
C81—Si5—C87	112.48 (17)	O23—C158—C159	107.3 (4)
C81—Si5—C91	109.99 (16)	C157—C158—H158	108.9
C91—Si5—C87	110.83 (18)	C157—C158—C159	112.4 (6)
C97—O13—Si5	126.2 (2)	C159—C158—H158	108.9
C98—O14—H14G	109.5	O24—C159—C158	112.8 (11)
C99—O15—H15H	109.5	O24—C159—H15F	109.0
C82—C81—Si5	122.4 (3)	O24—C159—H15G	109.0
C82—C81—C86	116.9 (3)	C158—C159—H15F	109.0
C86—C81—Si5	120.7 (3)	C158—C159—H15G	109.0
C81—C82—H82	119.5	H15F—C159—H15G	107.8
C83—C82—C81	121.0 (4)	C257—O32—Si8	137.0 (19)
C83—C82—H82	119.5	C258—O33—H33	109.5
C82—C83—H83	119.7	C259—O34—H34	109.5
C84—C83—C82	120.5 (4)	O32—C257—H25A	108.6
C84—C83—H83	119.7	O32—C257—H25B	108.6
C83—C84—H84	120.0	O32—C257—C258	114.7 (18)
C83—C84—C85	120.1 (4)	H25A—C257—H25B	107.6
C85—C84—H84	119.9	C258—C257—H25A	108.6
C84—C85—H85	120.1	C258—C257—H25B	108.6
C84—C85—C86	119.8 (4)	O33—C258—C257	109.9 (14)
C86—C85—H85	120.1	O33—C258—H258	108.5
C81—C86—H86	119.2	O33—C258—C259	108.3 (12)
C85—C86—C81	121.6 (4)	C257—C258—H258	108.5
C85—C86—H86	119.2	C259—C258—C257	113.1 (13)
C88—C87—Si5	110.8 (3)	C259—C258—H258	108.5
C89—C87—Si5	110.2 (3)	O34—C259—C258	113 (3)
C89—C87—C88	107.2 (4)	O34—C259—H25C	108.9
C89—C87—C90	109.7 (4)	O34—C259—H25D	108.9
C90—C87—Si5	109.4 (3)	C258—C259—H25C	108.9
C90—C87—C88	109.6 (4)	C258—C259—H25D	108.9
C87—C88—H88A	109.5	H25C—C259—H25D	107.8
			
Si1—O1—C17—C18	166.7 (2)	C82—C81—C86—C85	−1.1 (5)
Si1—C1—C2—C3	177.6 (3)	C82—C83—C84—C85	−0.1 (7)
Si1—C1—C6—C5	−177.9 (3)	C83—C84—C85—C86	−0.3 (7)
Si1—C11—C12—C13	176.2 (3)	C84—C85—C86—C81	0.9 (6)
Si1—C11—C16—C15	−176.6 (3)	C86—C81—C82—C83	0.7 (6)
O1—Si1—C1—C2	157.0 (3)	C87—Si5—O13—C97	154.9 (3)
O1—Si1—C1—C6	−24.5 (3)	C87—Si5—C81—C82	117.6 (3)
O1—Si1—C7—C8	−50.5 (2)	C87—Si5—C81—C86	−62.2 (3)
O1—Si1—C7—C9	68.2 (2)	C87—Si5—C91—C92	114.2 (3)
O1—Si1—C7—C10	−173.7 (2)	C87—Si5—C91—C96	−63.9 (3)
O1—Si1—C11—C12	−74.8 (3)	C91—Si5—O13—C97	−86.6 (3)
O1—Si1—C11—C16	102.0 (3)	C91—Si5—C81—C82	−6.5 (3)
O1—C17—C18—O2	−67.1 (3)	C91—Si5—C81—C86	173.7 (3)
O1—C17—C18—C19	55.5 (4)	C91—Si5—C87—C88	−165.3 (3)
O2—C18—C19—O3	−57.8 (4)	C91—Si5—C87—C89	76.3 (4)
C1—Si1—O1—C17	−69.4 (3)	C91—Si5—C87—C90	−44.4 (4)
C1—Si1—C7—C8	−166.3 (2)	C91—C92—C93—C94	−0.1 (7)
C1—Si1—C7—C9	−47.6 (3)	C92—C91—C96—C95	−1.6 (5)
C1—Si1—C7—C10	70.5 (3)	C92—C93—C94—C95	−0.5 (7)
C1—Si1—C11—C12	42.8 (3)	C93—C94—C95—C96	0.0 (7)
C1—Si1—C11—C16	−140.4 (3)	C94—C95—C96—C91	1.1 (6)
C1—C2—C3—C4	0.7 (7)	C96—C91—C92—C93	1.1 (6)
C2—C1—C6—C5	0.7 (5)	C97—C98—C99—O15	62.6 (4)
C2—C3—C4—C5	0.1 (7)	Si6—C101—C102—C103	−177.4 (3)
C3—C4—C5—C6	−0.4 (6)	Si6—C101—C106—C105	177.1 (3)
C4—C5—C6—C1	0.0 (5)	Si6—C111—C112—C113	−178.7 (3)
C6—C1—C2—C3	−1.0 (6)	Si6—C111—C116—C115	179.3 (3)
C7—Si1—O1—C17	172.6 (2)	Si6—O16—C117—C118	−91.5 (8)
C7—Si1—C1—C2	−89.4 (3)	Si6—O216—C217—C218	−161.3 (13)
C7—Si1—C1—C6	89.1 (3)	C101—Si6—C107—C108	54.2 (3)
C7—Si1—C11—C12	168.3 (2)	C101—Si6—C107—C109	172.8 (3)
C7—Si1—C11—C16	−15.0 (3)	C101—Si6—C107—C110	−64.5 (3)
C11—Si1—O1—C17	49.0 (3)	C101—Si6—C111—C112	−47.0 (3)
C11—Si1—C1—C2	38.5 (3)	C101—Si6—C111—C116	133.9 (3)
C11—Si1—C1—C6	−142.9 (3)	C101—Si6—O16—C117	89.7 (7)
C11—Si1—C7—C8	69.2 (3)	C101—Si6—O216—C217	68.7 (16)
C11—Si1—C7—C9	−172.1 (2)	C101—C102—C103—C104	0.1 (7)
C11—Si1—C7—C10	−54.0 (3)	C102—C101—C106—C105	−0.5 (6)
C11—C12—C13—C14	1.4 (5)	C102—C103—C104—C105	−0.1 (7)
C12—C11—C16—C15	0.2 (5)	C103—C104—C105—C106	−0.3 (7)
C12—C13—C14—C15	−1.4 (5)	C104—C105—C106—C101	0.6 (6)
C13—C14—C15—C16	0.8 (5)	C106—C101—C102—C103	0.1 (6)
C14—C15—C16—C11	−0.2 (5)	C107—Si6—C101—C102	95.6 (3)
C16—C11—C12—C13	−0.8 (5)	C107—Si6—C101—C106	−81.9 (3)
C17—C18—C19—O3	−179.2 (3)	C107—Si6—C111—C112	−170.8 (3)
Si2—O4—C37—C38	−131.5 (3)	C107—Si6—C111—C116	10.2 (4)
Si2—C21—C22—C23	178.3 (3)	C107—Si6—O16—C117	−153.2 (6)
Si2—C21—C26—C25	−178.7 (3)	C107—Si6—O216—C217	−174.0 (13)
Si2—C31—C32—C33	−179.1 (3)	C111—Si6—C101—C102	−31.4 (3)
Si2—C31—C36—C35	179.0 (3)	C111—Si6—C101—C106	151.1 (3)
Si3—O7—C57—C58	156.1 (3)	C111—Si6—C107—C108	176.1 (3)
Si3—C41—C42—C43	−179.6 (4)	C111—Si6—C107—C109	−65.2 (3)
Si3—C41—C46—C45	−179.9 (3)	C111—Si6—C107—C110	57.5 (3)
Si3—C51—C52—C53	−179.8 (3)	C111—Si6—O16—C117	−31.4 (7)
Si3—C51—C56—C55	179.9 (3)	C111—Si6—O216—C217	−43.3 (16)
O4—Si2—C21—C22	−104.8 (3)	C111—C112—C113—C114	−0.9 (6)
O4—Si2—C21—C26	73.9 (3)	C112—C111—C116—C115	0.2 (5)
O4—Si2—C27—C28	56.4 (3)	C112—C113—C114—C115	0.8 (6)
O4—Si2—C27—C29	179.6 (3)	C113—C114—C115—C116	−0.1 (5)
O4—Si2—C27—C30	−60.9 (3)	C114—C115—C116—C111	−0.4 (5)
O4—Si2—C31—C32	−159.7 (3)	C116—C111—C112—C113	0.4 (5)
O4—Si2—C31—C36	21.7 (3)	O16—Si6—C101—C102	−154.4 (4)
O4—C37—C38—O5	179.3 (3)	O16—Si6—C101—C106	28.1 (4)
O4—C37—C38—C39	−58.4 (4)	O16—Si6—C107—C108	−65.9 (4)
O5—C38—C39—O6	−68.0 (4)	O16—Si6—C107—C109	52.8 (4)
O7—Si3—C41—C42	113.4 (3)	O16—Si6—C107—C110	175.4 (4)
O7—Si3—C41—C46	−65.7 (3)	O16—Si6—C111—C112	78.2 (4)
O7—Si3—C47—C48	57.6 (3)	O16—Si6—C111—C116	−100.8 (4)
O7—Si3—C47—C49	176.7 (3)	O16—C117—C118—O17	172.6 (5)
O7—Si3—C47—C50	−61.0 (4)	O16—C117—C118—C119	−62.4 (7)
O7—Si3—C51—C52	157.6 (3)	O17—C118—C119—O18	57.0 (6)
O7—Si3—C51—C56	−23.1 (3)	C117—C118—C119—O18	−67.1 (6)
O7—C57—C58—O8	−67.7 (4)	O216—Si6—C101—C102	−144.9 (7)
O7—C57—C58—C59	55.6 (4)	O216—Si6—C101—C106	37.6 (7)
O8—C58—C59—O9	−64.2 (4)	O216—Si6—C107—C108	−56.7 (7)
C21—Si2—O4—C37	9.5 (4)	O216—Si6—C107—C109	62.0 (7)
C21—Si2—C27—C28	−66.8 (4)	O216—Si6—C107—C110	−175.4 (7)
C21—Si2—C27—C29	56.4 (3)	O216—Si6—C111—C112	60.0 (5)
C21—Si2—C27—C30	176.0 (3)	O216—Si6—C111—C116	−119.1 (5)
C21—Si2—C31—C32	−40.4 (3)	O216—C217—C218—O217	−177.8 (10)
C21—Si2—C31—C36	141.1 (3)	O216—C217—C218—C219	−53.8 (13)
C21—C22—C23—C24	0.8 (6)	O217—C218—C219—O218	−51.5 (15)
C22—C21—C26—C25	0.1 (5)	C217—C218—C219—O218	−174.7 (8)
C22—C23—C24—C25	−0.9 (6)	Si7—O19—C137—C138	169.6 (3)
C23—C24—C25—C26	0.5 (6)	Si7—C131—C132—C133	179.6 (4)
C24—C25—C26—C21	−0.1 (6)	Si7—C131—C136—C135	−179.0 (4)
C26—C21—C22—C23	−0.4 (5)	Si7—C121—C122—C123	179.0 (12)
C27—Si2—O4—C37	−116.1 (4)	Si7—C121—C126—C125	−179.3 (9)
C27—Si2—C21—C22	15.6 (4)	Si7—C161—C162—C163	175.7 (15)
C27—Si2—C21—C26	−165.7 (3)	Si7—C161—C166—C165	−175.9 (14)
C27—Si2—C31—C32	86.6 (3)	O19—Si7—C127—C128	174.8 (3)
C27—Si2—C31—C36	−92.0 (3)	O19—Si7—C127—C129	56.1 (3)
C31—Si2—O4—C37	127.5 (4)	O19—Si7—C127—C130	−63.1 (3)
C31—Si2—C21—C22	139.6 (3)	O19—Si7—C131—C132	153.8 (3)
C31—Si2—C21—C26	−41.7 (3)	O19—Si7—C131—C136	−26.1 (4)
C31—Si2—C27—C28	169.5 (3)	O19—Si7—C121—C122	120.3 (15)
C31—Si2—C27—C29	−67.3 (3)	O19—Si7—C121—C126	−58.6 (17)
C31—Si2—C27—C30	52.3 (3)	O19—C137—C138—O20	−80.1 (4)
C31—C32—C33—C34	−0.3 (6)	O19—C137—C138—C139	48.4 (4)
C32—C31—C36—C35	0.3 (6)	O20—C138—C139—O21	−56.3 (4)
C32—C33—C34—C35	1.3 (6)	C127—Si7—O19—C137	45.0 (4)
C33—C34—C35—C36	−1.5 (6)	C127—Si7—C131—C132	−86.1 (4)
C34—C35—C36—C31	0.7 (6)	C127—Si7—C131—C136	94.0 (4)
C36—C31—C32—C33	−0.5 (6)	C127—Si7—C121—C122	−6 (2)
C37—C38—C39—O6	172.5 (3)	C127—Si7—C121—C126	175.1 (12)
C41—Si3—O7—C57	−54.6 (3)	C131—Si7—O19—C137	163.0 (3)
C41—Si3—C47—C48	−177.4 (3)	C131—Si7—C127—C128	60.7 (3)
C41—Si3—C47—C49	−58.2 (3)	C131—Si7—C127—C129	−58.0 (3)
C41—Si3—C47—C50	64.1 (4)	C131—Si7—C127—C130	−177.1 (3)
C41—Si3—C51—C52	41.1 (3)	C131—Si7—C121—C122	−129.4 (16)
C41—Si3—C51—C56	−139.5 (3)	C131—Si7—C121—C126	51.8 (15)
C41—C42—C43—C44	−0.1 (7)	C131—C132—C133—C134	−0.9 (8)
C42—C41—C46—C45	1.0 (5)	C132—C131—C136—C135	1.1 (7)
C42—C43—C44—C45	0.4 (7)	C132—C133—C134—C135	1.6 (8)
C43—C44—C45—C46	0.0 (6)	C133—C134—C135—C136	−1.0 (8)
C44—C45—C46—C41	−0.7 (6)	C134—C135—C136—C131	−0.4 (8)
C46—C41—C42—C43	−0.6 (6)	C136—C131—C132—C133	−0.5 (7)
C47—Si3—O7—C57	73.1 (3)	C137—C138—C139—O21	175.0 (3)
C47—Si3—C41—C42	−12.2 (4)	C121—Si7—O19—C137	−83.2 (8)
C47—Si3—C41—C46	168.8 (3)	C121—Si7—C127—C128	−61.8 (8)
C47—Si3—C51—C52	−84.4 (3)	C121—Si7—C127—C129	179.5 (8)
C47—Si3—C51—C56	94.9 (3)	C121—Si7—C127—C130	60.4 (8)
C51—Si3—O7—C57	−169.9 (3)	C121—Si7—C131—C132	40.9 (5)
C51—Si3—C41—C42	−134.7 (3)	C121—Si7—C131—C136	−139.1 (5)
C51—Si3—C41—C46	46.3 (3)	C121—C122—C123—C124	3 (2)
C51—Si3—C47—C48	−55.5 (3)	C122—C121—C126—C125	2 (2)
C51—Si3—C47—C49	63.7 (3)	C122—C123—C124—C125	−4 (3)
C51—Si3—C47—C50	−174.1 (3)	C123—C124—C125—C126	3 (3)
C51—C52—C53—C54	−0.3 (5)	C124—C125—C126—C121	−2.3 (19)
C52—C51—C56—C55	−0.8 (5)	C126—C121—C122—C123	−2 (2)
C52—C53—C54—C55	−0.2 (6)	C161—Si7—O19—C137	−79.1 (11)
C53—C54—C55—C56	0.3 (6)	C161—Si7—C127—C128	−60.7 (10)
C54—C55—C56—C51	0.3 (5)	C161—Si7—C127—C129	−179.4 (10)
C56—C51—C52—C53	0.8 (5)	C161—Si7—C127—C130	61.5 (10)
C57—C58—C59—O9	174.0 (3)	C161—Si7—C131—C132	35.3 (6)
Si4—O10—C77—C78	−164.8 (3)	C161—Si7—C131—C136	−144.6 (6)
Si4—C61—C62—C63	174.3 (4)	C161—C162—C163—C164	−4 (3)
Si4—C61—C66—C65	−174.8 (4)	C162—C161—C166—C165	−5 (3)
Si4—C71—C72—C73	−172.6 (3)	C162—C163—C164—C165	3 (3)
Si4—C71—C76—C75	172.5 (4)	C163—C164—C165—C166	−3 (3)
O10—Si4—C61—C62	−163.7 (3)	C164—C165—C166—C161	4 (3)
O10—Si4—C61—C66	12.1 (3)	C166—C161—C162—C163	5 (3)
O10—Si4—C67—C68	−171.4 (3)	Si8—C141—C142—C143	−175.7 (4)
O10—Si4—C67—C69	66.7 (3)	Si8—C141—C146—C145	174.8 (4)
O10—Si4—C67—C70	−53.1 (3)	Si8—C151—C152—C153	174.3 (3)
O10—Si4—C71—C72	−177.6 (3)	Si8—C151—C156—C155	−173.8 (3)
O10—Si4—C71—C76	8.9 (4)	Si8—O22—C157—C158	130.4 (8)
O10—C77—C78—O11	73.7 (4)	Si8—O32—C257—C258	151 (2)
O10—C77—C78—C79	−168.4 (3)	C141—Si8—C147—C148	−51.9 (4)
O11—C78—C79—O12	−66.7 (4)	C141—Si8—C147—C149	70.3 (3)
C61—Si4—O10—C77	−164.3 (3)	C141—Si8—C147—C150	−170.5 (3)
C61—Si4—C67—C68	−58.5 (3)	C141—Si8—C151—C152	121.7 (3)
C61—Si4—C67—C69	179.6 (3)	C141—Si8—C151—C156	−63.6 (3)
C61—Si4—C67—C70	59.8 (3)	C141—Si8—O22—C157	−41.2 (7)
C61—Si4—C71—C72	72.8 (3)	C141—Si8—O32—C257	−28 (3)
C61—Si4—C71—C76	−100.7 (3)	C141—C142—C143—C144	0.8 (7)
C61—C62—C63—C64	0.1 (7)	C142—C141—C146—C145	−1.4 (6)
C62—C61—C66—C65	1.2 (6)	C142—C143—C144—C145	−1.3 (7)
C62—C63—C64—C65	1.8 (8)	C143—C144—C145—C146	0.4 (7)
C63—C64—C65—C66	−2.2 (9)	C144—C145—C146—C141	1.0 (7)
C64—C65—C66—C61	0.7 (8)	C146—C141—C142—C143	0.5 (6)
C66—C61—C62—C63	−1.6 (6)	C147—Si8—C141—C142	−128.6 (3)
C67—Si4—O10—C77	−44.9 (4)	C147—Si8—C141—C146	55.4 (4)
C67—Si4—C61—C62	77.6 (3)	C147—Si8—C151—C152	−110.7 (3)
C67—Si4—C61—C66	−106.6 (3)	C147—Si8—C151—C156	64.0 (3)
C67—Si4—C71—C72	−52.3 (4)	C147—Si8—O22—C157	−163.5 (6)
C67—Si4—C71—C76	134.2 (3)	C147—Si8—O32—C257	−156 (2)
C71—Si4—O10—C77	82.1 (4)	C151—Si8—C141—C142	−4.6 (4)
C71—Si4—C61—C62	−48.8 (3)	C151—Si8—C141—C146	179.4 (3)
C71—Si4—C61—C66	127.0 (3)	C151—Si8—C147—C148	−176.6 (3)
C71—Si4—C67—C68	64.2 (3)	C151—Si8—C147—C149	−54.4 (3)
C71—Si4—C67—C69	−57.7 (3)	C151—Si8—C147—C150	64.8 (3)
C71—Si4—C67—C70	−177.5 (2)	C151—Si8—O22—C157	81.2 (7)
C71—C72—C73—C74	−0.8 (7)	C151—Si8—O32—C257	88 (3)
C72—C71—C76—C75	−1.6 (7)	C151—C152—C153—C154	−0.1 (6)
C72—C73—C74—C75	1.0 (7)	C152—C151—C156—C155	1.1 (5)
C73—C74—C75—C76	−1.6 (8)	C152—C153—C154—C155	0.9 (6)
C74—C75—C76—C71	1.9 (9)	C153—C154—C155—C156	−0.6 (6)
C76—C71—C72—C73	1.1 (6)	C154—C155—C156—C151	−0.4 (5)
C77—C78—C79—O12	173.7 (3)	C156—C151—C152—C153	−0.8 (6)
Si5—O13—C97—C98	−161.7 (2)	O22—Si8—C141—C142	118.2 (4)
Si5—C81—C82—C83	−179.1 (3)	O22—Si8—C141—C146	−57.8 (5)
Si5—C81—C86—C85	178.8 (3)	O22—Si8—C147—C148	66.7 (5)
Si5—C91—C92—C93	−177.1 (3)	O22—Si8—C147—C149	−171.1 (5)
Si5—C91—C96—C95	176.5 (3)	O22—Si8—C147—C150	−51.9 (5)
O13—Si5—C81—C82	−126.1 (3)	O22—Si8—C151—C152	−0.7 (5)
O13—Si5—C81—C86	54.0 (3)	O22—Si8—C151—C156	174.0 (5)
O13—Si5—C87—C88	−48.3 (3)	O22—C157—C158—O23	−63.1 (8)
O13—Si5—C87—C89	−166.7 (3)	O22—C157—C158—C159	56.6 (7)
O13—Si5—C87—C90	72.6 (4)	O23—C158—C159—O24	−65.2 (12)
O13—Si5—C91—C92	−0.4 (3)	C157—C158—C159—O24	173.4 (12)
O13—Si5—C91—C96	−178.4 (3)	O32—Si8—C141—C142	105.5 (8)
O13—C97—C98—O14	−62.3 (4)	O32—Si8—C141—C146	−70.5 (8)
O13—C97—C98—C99	60.4 (4)	O32—Si8—C147—C148	72.1 (11)
O14—C98—C99—O15	−173.5 (2)	O32—Si8—C147—C149	−165.7 (11)
C81—Si5—O13—C97	33.8 (3)	O32—Si8—C147—C150	−46.5 (11)
C81—Si5—C87—C88	71.1 (4)	O32—Si8—C151—C152	7.3 (10)
C81—Si5—C87—C89	−47.3 (4)	O32—Si8—C151—C156	−178.0 (10)
C81—Si5—C87—C90	−168.0 (3)	O32—C257—C258—O33	−170.4 (17)
C81—Si5—C91—C92	−120.8 (3)	O32—C257—C258—C259	−49 (2)
C81—Si5—C91—C96	61.1 (3)	O33—C258—C259—O34	−49 (3)
C81—C82—C83—C84	−0.1 (7)	C257—C258—C259—O34	−172 (3)

### (S)-1-O-t-butyldiphenylsilylpropane-2,3-diol (2) Hydrogen-bond geometry (Å, º)

**Table:**

*D*—H···*A*	*D*—H	H···*A*	*D*···*A*	*D*—H···*A*
O2—H2···O18	0.84	2.03	2.841 (4)	163
O2—H2···O218	0.84	2.11	2.828 (7)	143
O3—H3*A*···O23	0.84	1.93	2.752 (4)	165
O5—H5*A*···O9	0.84	1.81	2.651 (4)	176
O6—H6*A*···O14^i^	0.84	1.90	2.707 (3)	161
O8—H8···O6	0.84	2.06	2.814 (3)	149
O9—H9···O24	0.84	2.06	2.84 (2)	154
O11—H11···O6	0.84	1.95	2.782 (4)	174
O12—H12*G*···O24	0.84	2.33	3.00 (2)	137
O14—H14*G*···O13	0.84	2.44	2.839 (3)	110
O15—H15*H*···O21^ii^	0.84	1.85	2.676 (3)	169
O17—H17···O12	0.84	1.83	2.615 (4)	156
O18—H18···O15	0.84	1.93	2.745 (4)	164
C119—H11*G*···O3	0.99	2.39	3.222 (7)	141
O218—H218···O15	0.84	1.76	2.599 (8)	176
O20—H20···O5	0.84	2.00	2.794 (4)	157
O21—H21···O8	0.84	1.89	2.727 (4)	174
C138—H138···O3^i^	1.00	2.50	3.320 (5)	139
O23—H23···O17	0.84	1.87	2.697 (5)	169
O24—H24···O2	0.84	1.96	2.78 (2)	163

Symmetry codes: (i) *x*−1, *y*, *z*; (ii) *x*+1, *y*, *z*.
